# Shared structural mechanisms of alternating access between the secondary peptide transporter SbmA and ABC transporters

**DOI:** 10.1038/s41467-026-71633-3

**Published:** 2026-04-15

**Authors:** Thijs W. Ettema, Satomi Inaba-Inoue, Chancievan Thangaratnarajah, Leticia Alves da Silva, Nikas Senning, Amy Clarke, Piotr Stepien, Anokhi Shah, Yue Ma, Katie Hardman, Sophia David, Hassane El Mkami, Jonathan G. Heddle, Norimichi Nomura, Satoshi Ogasawara, So Iwata, Dmitry Ghilarov, Christos Pliotas, Thomas Stockner, Dirk J. Slotboom, Konstantinos Beis

**Affiliations:** 1 Membrane Enzymology Group, Groningen, Biomolecular Sciences and Biotechnology, Faculty of Science and Engineering, University of Groningen, Groningen, The Netherlands; 2 Department of Life Sciences, Imperial College London, London, UK; 3 Rutherford Appleton Laboratory, Research Complex at Harwell, Oxfordshire, UK; 4 Institute of Pharmacology, Medical University of Vienna, Vienna, Austria; 5 Malopolska Centre of Biotechnology, Jagiellonian University, Krakow, Poland; 6 BioEmPiRe Centre for Structural Biological EPR Spectroscopy, School of Biological Sciences, Faculty of Biology, Medicine and Health, University of Manchester, Manchester, UK; 7 Manchester Institute of Biotechnology, University of Manchester, Manchester, UK; 8 Astbury Centre for Structural Molecular Biology, School of Biomedical Sciences, University of Leeds, Leeds, UK; 9 Centre for Genomic Pathogen Surveillance, Pandemic Sciences Institute, University of Oxford, Oxford, UK; 10 School of Physics and Astronomy, University of St. Andrews, St. Andrews, UK; 11 Department of Cell Biology, Graduate School of Medicine, Kyoto University, Kyoto, Japan; 12 Present Address: Structural Biology Research Center, Institute of Materials Structure, Science, High Energy Accelerator Research Organization, Tsukuba, Japan; 13 Present Address: Steinmetz Building, Granta Park, Nxera Pharma, Cambridge, UK; 14 Present Address: Centre for Programmable Biological Matter, Durham University, Durham, UK; 15 Present Address: Department of Biochemistry, University of Oxford, Oxford, UK

**Keywords:** Membrane proteins, Cryoelectron microscopy, Structural biology, Biophysics

## Abstract

SbmA is a membrane transporter from *Escherichia coli* that imports antimicrobial peptides. SbmA belongs to the SbmA-like peptide transporter (SLiPT) family. Although the protein is a secondary active transporter that is energized by the proton gradient, it is structurally related to the transmembrane domain (TMD) of ATP-binding cassette (ABC) transporters. SbmA therefore bridges the structural divide between primary and secondary transporters. However, it remains unclear, if SbmA also shares the mechanism of alternating access with ABC transporters, because only a single (outward-open) state is resolved. Here, we show by sequence analysis that SbmA is likely evolved from the TMD of an early ancestor of the ABC transporter YddA. We determine the cryogenic electron microscopy structures of SbmA in occluded and inward-facing states. These conformations closely resemble equivalent states found in ABC transporters, indicating a shared structural mechanism of transport. In contrast to ABC transporters, where nucleotide binding, hydrolysis and release steer conformational changes necessary for substrate translocation, electron paramagnetic resonance (EPR) spectroscopy and molecular dynamics (MD) simulations reveal how pH changes induce conformational transitions in SbmA, consistent with a mechanism of substrate internalization that utilizes the transmembrane proton gradient.

## Abstract (web summary)

SbmA is a proton-driven transporter that imports antimicrobial peptides andstructurally resembles the transmembrane domain of ABC-transporters. Here, authors show through cryo-EM structures, EPR spectroscopy, and MD simulations that SbmA undergoes ABC-transporter-like conformational changes consistent with an alternating-access transport mechanism.

## Introduction

Under conditions of nutrient exhaustion, bacteria synthesize and release antimicrobial peptides (AMPs). These peptides exhibit potent antibacterial activity against closely related species, allowing the producing bacteria to secure more nutrients for survival^1,2^. While some AMPs target and damage the membrane, several members of non-ribosomally synthesized peptides (NRPs) and ribosomally-synthesized post-translationally modified peptides (RiPPs) inhibit key enzymes in the cytoplasm, thereby causing cell death. Uptake of NRPs and RiPPs into the target cell is facilitated by outer membrane siderophore receptors, such as FhuA, or porins, such as OmpF and OmpC, and the inner membrane transporter SbmA^3–5^.

SbmA is found in the inner membrane of *Escherichia coli*, and it has been implicated in the import of a wide variety of structurally diverse molecules (including peptides, peptide derivatives, and aminoglycosides)^4,6–10^ (for a review see: Slotboom et al.^11^). Initially, SbmA was shown to be involved in the import of the antimicrobial peptide microcin B17 (MccB17). Deletion or disruption of the *sbmA* gene increased resistance to MccB17, indicating its importance in internalizing antimicrobial agents ^4^. Beyond MccB17, SbmA is also essential for the internalization of other antimicrobial agents with intracellular targets. These include positively charged eukaryotic proline-rich peptide Bac7, various AMPs, aminoglycoside antibiotics, peptide nucleic acids, and peptide morpholino oligomers^6–10,12–14^. Despite its established role in antimicrobial agent transport, the precise physiological function of SbmA remains unclear, while its promiscuity suggests that AMPs exploit SbmA for internalization.

Closely related proteins, such as BacA from the leguminous plant symbiont *Sinorhizobium meliloti*, shed light on the function of SbmA^15^. BacA is essential for host colonization and chronic infection of root nodule cells^16^. It transports nodule-specific cysteine-rich peptides released by the host cell. BacA from *Brucella abortis* and the distantly related protein Rv1819c from *Mycobacterium tuberculosis* (BacA_Mt_) are required for chronic infection in mouse models^17,18^. Disruption of SbmA in avian pathogenic *E. coli* (APEC) reduces its virulence^17–19^.

Insights into the mechanism of AMP transport by SbmA and BacA have been provided by structural and functional studies. We previously determined the cryogenic electron microscopy (cryo-EM) structures of *E. coli* SbmA and *R. meliloti* BacA, revealing a different fold for secondary transporters termed the SbmA-like peptide transporter (SLiPT) fold^20^. Both SbmA and BacA are homodimers consisting of eight membrane-spanning helices (TMs) per protomer. The core transmembrane domain (TMD) comprises 12 TMs (six from each protomer, TMs 1–6), which in our previously determined structures adopt an outward-open conformation with the central aqueous cavity accessible to the periplasm. Two additional helices (TM0a and TM0b) flank the TMD, forming two peripheral domains (TM0). The overall structure of TMs 1–6 resembles that of type IV ABC transporters^21^, including Rv1819c (cobalamin transporter) from *M. tuberculosi*s^22^, which overlaps functionally with SbmA^17,23^. Unlike Rv1819c, which contains a nucleotide-binding domain (NBD), SLiPTs do not rely on ATP for transport. Instead, one or more protons are co-transported with the substrate^20,24,25^. The proton translocation pathway in SLiPTs involves a ‘glutamate ladder, formed by conserved glutamates from both protomers within the TMD^20^. Unlike Rv1819c or other type IV ABC transporters, the TMD of SLiPTs lacks the coupling helices required for association with NBDs^11,20^, as seen in full-length ABC transporters^11,22^. The previously determined cryo-EM structures of SbmA^20^ revealed structural similarity with type IV ABC transporters^21^, but the evolutionary origin remained elusive. It also remains unclear if the conformational transitions of type IV ABC transporters have also been propagated in SbmA, because only a single conformational state of SbmA was captured that revealed the outward-open state of SbmA in which the central aqueous cavity is exposed to the periplasm and inaccessible to the cytosol. In addition, dynamic information needed for a full mechanistic understanding of the SbmA transport cycle is lacking.

In this study, we combine bioinformatics analysis, electron paramagnetic resonance (EPR) spectroscopy and molecular dynamics (MD) simulations to probe the evolutionary origin and mechanism of SbmA. Our bioinformatics indicate that the SbmA-like transporters have evolved from an ancestor of the type IV ABC transporter YddA. We also determine a series of cryo-EM structures of SbmA with the central aqueous cavity sealed off from the periplasm by closure of the outer gate, and accessible to the cytosol with the cytoplasmic gate open to different extents (wide or narrow opening inward-facing state) or closed (occluded state). The structural states of SbmA closely resemble the conformations found in type IV ABC transporters, strongly suggesting a common origin of the mechanism leading to alternating access in primary (ATP-driven) ABC transporters and the secondary transporters of the SLiPT family. By combining EPR and MD simulations, we propose how protonation of the glutamate ladder affects the conformational ensemble and induces conformational changes along the TMD that may allow the substrate to move across the membrane, suggesting a mechanism for AMP internalization and proton translocation.

## Results

### Evolution of SLiPT transporters

We used bioinformatic approaches to investigate the evolutionary origin of the relationship between SLiPT transporters and the TMD of type IV ABC transporters. For the analysis, we extracted 3897 protein sequences (≥300 aa) from Gram-negative bacteria annotated as “SbmA” or “YddA” in UniProtKB. The YddA family comprises full-length ABC transporters (with attached NBD) of unknown function and contains a homolog in the *E. coli* strain K12 (Uniprot ID: P31826) that shares 29.1% amino acid similarity with SbmA (Uniprot ID: P0AFY6) over the TMD. We included a further 252 proteins from the so-called BclA (BacA-like) family of inner membrane peptide transporters that are functionally similar to SbmA/BacA proteins, and like YddA, they contain an NBD. We also included a further 273 protein sequences from a recent study that classified the different orthologues^26^ to ensure full representation of all proteins (≥300 aa) used in this separate study. Finally, we included representatives of four other full-length type IV ABC transporters from the *E. coli* K12 strain, MsbA (lipid A export), YojI (unknown function, although one report demonstrated its role in microcin MccJ25 export), MdlA and MdlB (both predicted to be involved in multidrug resistance). These share lower similarity with SbmA over the TMD (19.5%, 19.5%, 21.7%, and 20.0%, respectively).

**Fig. 1:**
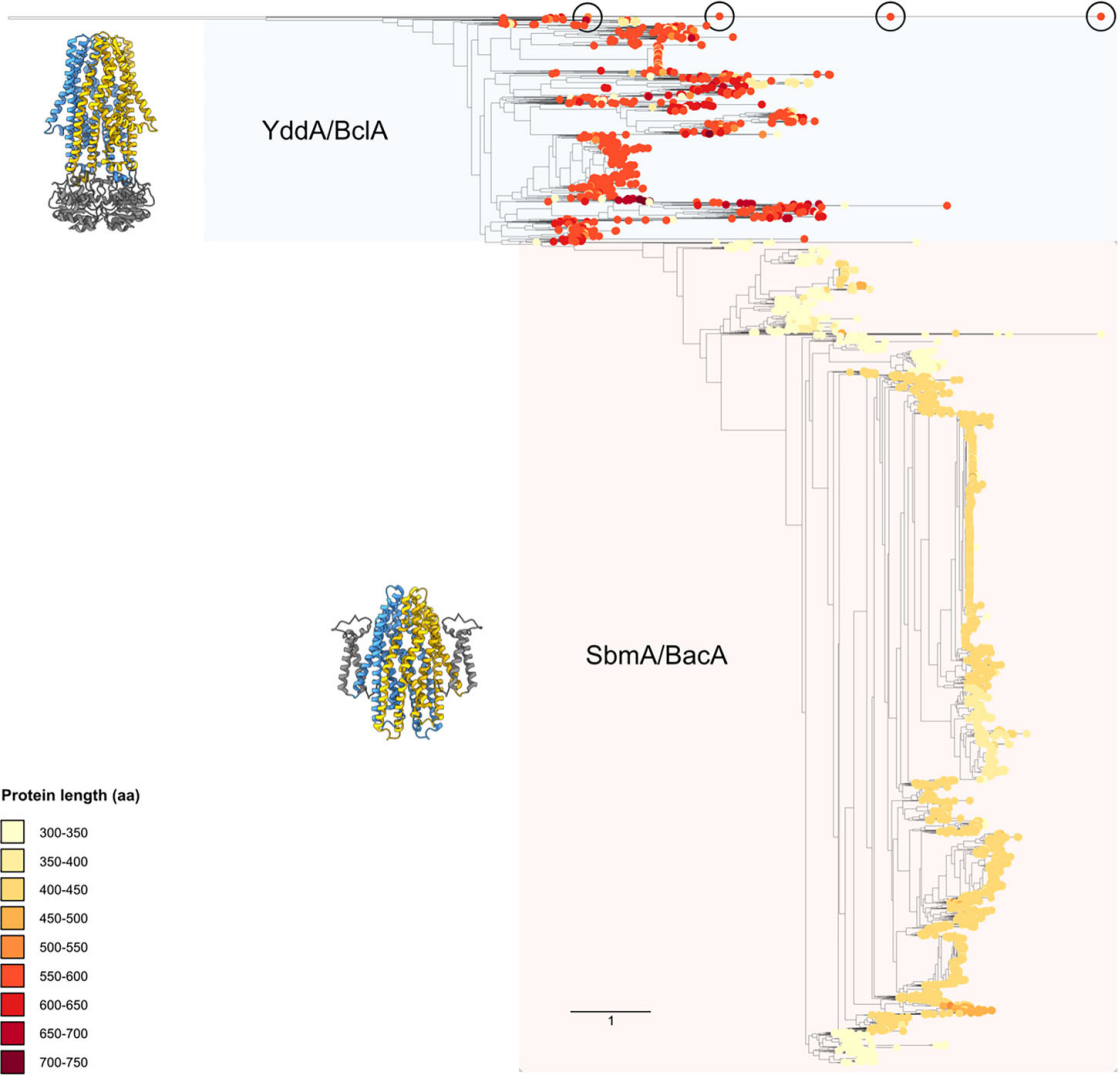
Phylogenetic tree of 4426 SbmA and related type IV ABC transporter proteins constructed using the transmembrane domain. The tree is rooted on a clade of proteins that are distantly related to SbmA, comprising YojI, MsbA, MdlB, and MdlA, highlighted by black rings (from left to right, respectively). Proteins from the YddA/BclA and SbmA/BacA families largely cluster separately in the tree. Tree nodes are colored by the length of the full-length proteins. The scale bar represents the number of substitutions per site. An interactive visualization of this tree with bootstrap values and more detailed metadata is available at https://microreact.org/project/sbma [https://microreact.org/project/sbma]. An AlphaFold3 model of YddA (AF-P31826-F1) and the cryo-EM structure of SbmA in the outward-facing state (PDB 7p34) are shown next to their corresponding cluster. The transmembrane domains in each protomer are colored in blue and yellow, respectively. The nucleotide-binding domains in YddA and the TM0 domains in SbmA are colored in gray.

We constructed a phylogenetic tree of the total 4426 protein sequences using the best-fitting evolutionary model (LG + G4) identified by ModelTest-NG^27^. The tree was rooted on an outgroup clade comprising the more distantly related ABC transporters, MsbA, YojI, MdlA, and MdlB. We found that most (97.9%; 3459/3532) of the proteins annotated as “SbmA” (or “SbmA/BacA”) in UniprotKB belonged to a single main clade in the tree (Fig. 1; see https://microreact.org/project/sbma, which contains bootstrap values). While there is some uncertainty in the tree topology, the branching suggests that this SbmA/BacA clade arose from an early YddA-like ancestor. The BclA proteins form a monophyletic cluster nested within the wider YddA clade. All proteins classified as “BacA” by Smith et al.^26^ were clustered in the SbmA clade, while those classified as “BclA” spanned much of the diversity of the YddA/BclA clade in our analysis.

Analysis of the YddA clade sequences showed that this family mostly encodes full-length ABC transporters, but there are examples of YddA sequences where the TMDs and NBDs are encoded on different genes in the same operon, as in *Neisseria meningitidis* strain NCTC8554 and *Serratia rubidaea* strain NCTC10036. Interestingly, in the *Rhizobium tibeticum* isolate CCBAU85039 genome, there are 2 annotated genes, *yddA_2* and *yddA_3*; YddA_3 belongs to the TMD, but there is no NBD gene associated under the same operon; YddA_2 is the NBD, but it is found in a different operon. We therefore speculate that the SbmA protein family is derived from the TMD of a YddA-like protein that became separated from the NBD.

### Structure determination

We have previously determined the structure of SbmA at pH 7.5 in an outward-open conformation in lipid nanodiscs^20^, both in the presence and in the absence of the Fab fragment Fab S11-1 (Table 1, identifier A). We reasoned that the SbmA protein could adopt other conformations at different pH triggered by changes in the protonation state of, for instance, the glutamate ladder. Indeed, we were able to solve the structures of SbmA in an inward-facing conformation in lipid nanodiscs at pH 5.5. Analysis of the particles revealed conformational heterogeneity, which after extensive classification resulted in four classes, all with a single Fab bound per dimeric SbmA, but differing in the extent of the opening on the cytoplasmic side (see Table 1, Supplementary Figs. 1 and 2). We modeled two maps of the best quality judged by map completeness (Supplementary Fig. 1), resulting in two models which we termed “inward-facing-wide” (3.44 Å resolution) and “inward-facing-narrow” (3.58 Å resolution) (Table 1, identifiers C and G). There was also a small population of particles that maintained the outward-facing conformation (Supplementary Fig. 1).

**Table 1:** Overview of cryo-EM maps of SbmA from our previous (identifier A, PDB 7p34) and current study (identifiers B-G)

Structure and identifier	Conformation	Conditions	Periplasmic gate distance Cα S123-S123′ (Å) L349-L349′ (Å)	Central gate distance Cα Y368-Y368′ (Å)	Cytosolic gate distance Cα Y285-Y285′ (Å)
	Outward-open	pH 7.5 lipid nanodiscs 2 Fabs	24 18	8	8
	Inward-occluded	pH 6.5 lipid nanodiscs	7 10	15	18
	Inward-facing-narrow	pH 5.5 lipid nanodiscs 1 Fab	8 10	20	20
	Inward-facing-narrow	pH 7.4 lipid nanodiscs 2 Sb	8 10	20	19
	Inward-facing- wide	pH 8.0 detergent	7 10	25	20
	Inward-facing- wide	pH 7.4 detergent 2 Sb	7 10	25	21
	Inward-facing- wide	pH 5.5 lipid nanodiscs 1 Fab	7 10	27	21

The models are colored by chain in blue and yellow with fiducial markers (Fab/sybodies) shown in gray. Conformations are assigned based on structural differences, illustrated by the distances between the Cα’s of residues in the proposed gating residues. Relevant conditions for protein preparation are included in the 3rd column.

To explore if conditions other than a change in the pH could also change the conformational ensemble, we additionally determined structures of SbmA in the presence of a sybody (Sb2) in detergent solution at pH 7.4. Sybodies (16 kDa) are smaller than the Fab fragment^28^, which could allow SbmA to sample conformations that were previously inaccessible due to the presence of the Fab. Under these conditions, SbmA did not adopt the outward-open conformation, previously found at pH 7.5 in lipid nanodiscs, but instead closely resembled the Fab-bound structures at pH 5.5, with two main populations: wide and narrow inward-facing conformations, obtained at resolutions of 3.14 and 3.24 Å, respectively (Table 1, identifiers D and F; Supplementary Figs. 3 and 4). Similar to the Fab-bound structures, the sybody is bound on the periplasmic side of SbmA, but in contrast to the Fab-bound structures, each SbmA protomer bound one sybody. We also found a less abundant third class of the SbmA dimer with a single bound sybody (Supplementary Figs. 3 and 5), which adopts an identical conformation to the inward-facing-wide structure observed in the presence of two sybodies (3.96 Å resolution). Regardless of the experimental conditions used, all the inward-facing-wide structures are very similar and can be superimposed with RMSD values of 0.53 to 2.16 Å. Similarly, the inward-facing-narrow structures can be superimposed with an RMSD of 1.33 Å.

We also determined the structure of SbmA in the absence of Fab or sybody at pH 8.0 in detergent solution (Supplementary Fig. 6) and at pH 6.5 in lipid nanodiscs containing *E. coli* polar lipids (Supplementary Fig. 7), instead of the synthetic lipids previously used in the case of the outward-open structure^20^. At pH 8.0, SbmA again adopted an inward-facing-wide conformation (Table 1, identifier E), rather than the outward-open conformation previously determined in nanodiscs at pH 7.5. Apparently, the use of detergent instead of lipid nanodiscs affected the structural ensemble. At pH 6.5, SbmA was in an inward-occluded conformation (Table 1, identifier B and Supplementary Fig. 8), in which the cytoplasmic opening was too small (~8 Å) for substrates like MccJ25 and Bac7(1–16) to pass. Notably, although the Bac7(1–16) peptide was added to the protein preparations used for these two structure determinations, we were unable to resolve any density for it. Similarly, in the previously determined structures of SbmA in the outward-open conformation, we were unable to resolve densities for MccB17 or bleomycin, even though these compounds were present at saturating concentrations. Apparently, none of the compounds adopt a well-defined binding pose.

### Overall structure

**Fig. 2:**
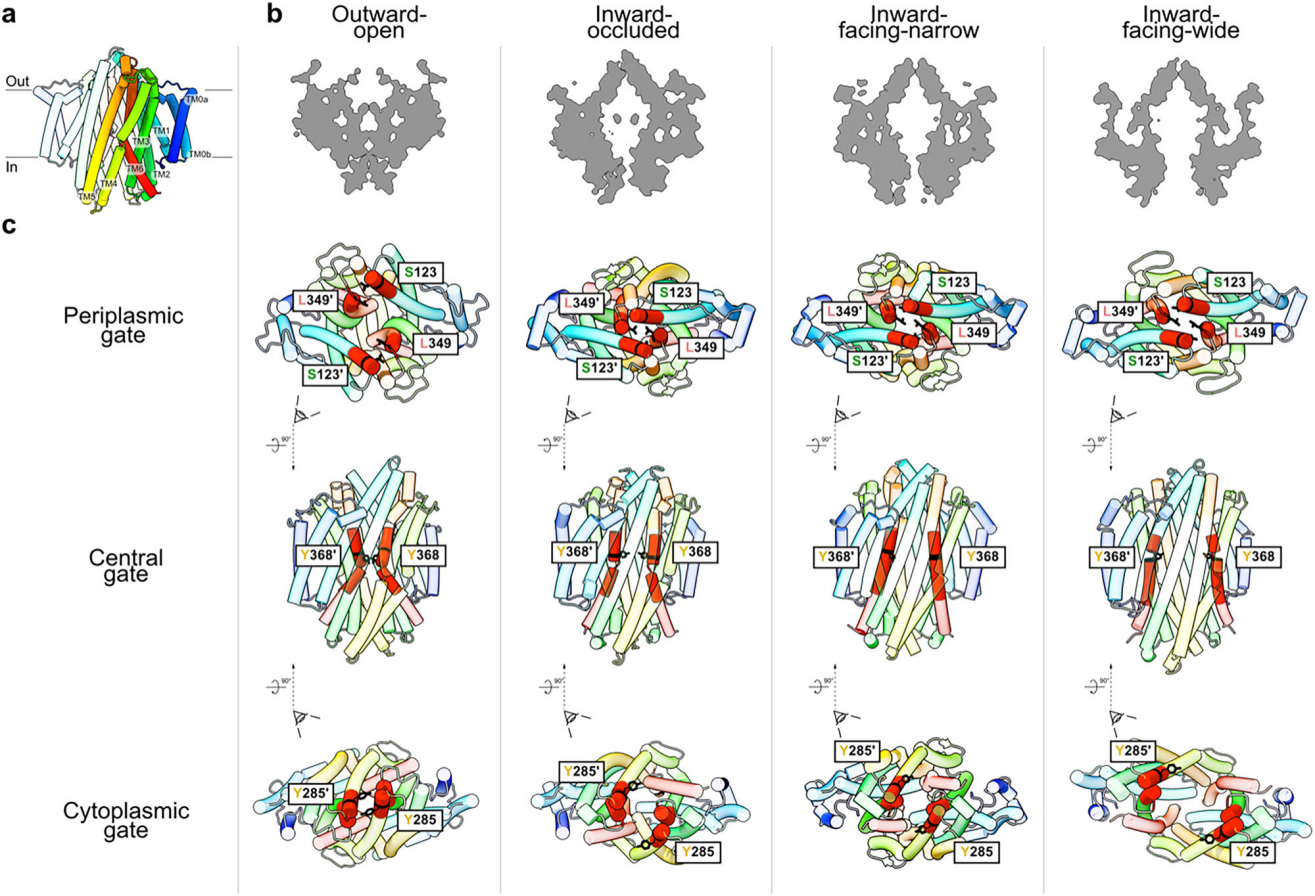
Comparison of outward-open and inward-facing structures of SbmA. **a** Overview of SbmA (inward-occluded) with helices represented as tubes, and one protomer colored from blue (N-terminus) to red (C-terminus). TMs mentioned in the text are annotated. **b** Shape of the central cavity illustrated by cross-sections of SbmA from surface maps in the outward-open (Table 1, identifier A), inward-occluded (Table 1, identifier B), inward-facing-narrow (Table 1, identifier C) and inward-facing-wide (Table 1, identifier G) conformation. The proteins are in the same orientation as in (**a**). **c** Views of SbmA from the periplasm (top panels), the membrane plane (middle) and the cytoplasm (bottom). The outward-open (Table 1, identifier A), inward-occluded (Table 1, identifier B), and inward-facing-wide (Table 1, identifier G) structures are depicted with helices represented as tubes, and both protomers colored from pale blue (N-terminus) to pale red (C-terminus). In each view, the segments involved in gating are highlighted in bright red, and prominent gating residues are represented as black sticks.

The inward-facing conformations (inward-facing-wide, inward-facing-narrow and inward-occluded) are distinctly different from the outward-open conformation (Fig. 2). Transition from the outward-open to the inward-facing conformations results in large structural rearrangements within the TMD and in changes of the central cavity (Fig. 2b). In the inward-facing conformations, the periplasmic gate has closed due to the movement of TMs 1–2 towards 5′–6′, and the cytoplasmic gate has opened because TMs 3–4 and TMs 3′–4′ separated. These global changes are consistent, regardless of the extent of opening on the cytoplasmic side (wide, narrow or occluded). We will describe the conformational differences between the outward-facing structure and the inward-facing structures, focusing on the two most extreme states of the identified classes: inward-facing-wide (identifier G in Table 1) and inward-occluded (identifier B in Table 1) states. It is noteworthy that TM0a and TM0b display minimal changes between the inward- and outward-facing states.

Cytoplasmic gate—the cytoplasmic gate is formed by TMs 2–3, 4–5 and the C-terminal end of TM 6b. Compared to the outward-open structure, all these elements have moved away from the 2-fold symmetry axis of the dimer, although to a lesser extent in the inward-occluded and inward-facing-narrow conformation than in the inward-facing-wide conformation (Fig. 2c and Supplementary Fig. 9). The opening of an access route on the cytoplasmic side is illustrated by the change in distance between Y285 and Y285′, which are proposed gating residues in TM 4 (Table 1). This distance increases from 7.8 Å in the outward-open conformation to 17.7 Å in the inward-occluded conformation and to 21.1 Å in the inward-facing-wide conformation.

A second cytoplasmic gate is located further towards the cytosol in the central cavity and is formed by residues R280 and Q189 from both protomers. In the inward-occluded structure (Table 1, identifier B), these four residues form hydrogen bonds across the two-fold symmetry axis, effectively closing off the access from the cytosol to the central cavity. However, small fenestrations (~8 Å in diameter) on either side of the two-fold axis are still present in each protomer, leaving the central cavity accessible from the cytoplasm for water and ions, but not to large substrates, such as MccJ25 (Supplementary Fig. 8). This second internal gate is open in the inward-facing-narrow and -wide conformations, therefore it appears that this is the last remaining point of contact before the cytoplasmic gate opens (Supplementary Fig. 8).

Central gate—within the bilayer region, the outward- and inward-facing conformations exhibit substantial structural differences. In all inward-facing structures, the TMs are located closer to the two-fold axis of the dimer on the periplasmic side and are further away from the symmetry axis at the cytoplasmic side. These conformational differences are enabled by flexible regions in TM 3–5 and 6a/b, allowing the periplasmic and cytoplasmic halves of the helices to move in opposite directions. As a result of this movement, the central cavity undergoes a transition from an hourglass shape in the outward-open conformation to a cone shape in the inward-facing-wide conformation (Fig. 2b, c).

The broken helix TM 6a/b has been previously proposed to play an important role in the opening and closing of the central aqueous cavity^20^. In the outward-open structure, a pronounced kink in the middle of TM 6 allows the side chains of Y368 and Y368′ to come into close proximity (3.8 Å), forming an internal gate that separates the glutamate ladder (Supplementary Fig. 10) on the cytoplasmic side of the gate from the aqueous cavity on the periplasmic side where the peptide substrate is expected to bind (Fig. 2c). In the inward-occluded state, the kink in TM 6 is still present, but less pronounced, as the side chains of the two tyrosine residues are further apart (8.9 Å), resulting in a modest opening of this central gate and movement of the glutamate ladder (Supplementary Fig. 10a). Additionally, since the cytoplasmic gate of SbmA is slightly open in this state via the two fenestrations, a connected cavity extends from the cytoplasm to the closed gate on the periplasmic end of SbmA (Fig. 2b). In the inward-facing-wide state, the kink in TM 6 has completely disappeared, and the helix is fully straightened. The distance between the central gate residues (Y368 and Y368′) has increased to 27.4 Å. Furthermore, the other helices that line most of the aqueous cavity (TMs 3, 4, and 5) are now located toward the periphery of SbmA, resulting in a further enlarged central cavity that is fully accessible from the cytoplasm (Fig. 2b, c).

Periplasmic gate—the protruding region on the periplasmic side of SbmA consists of the periplasmic ends of TMs 1–6 and the connecting loops. In the outward-open conformation, these regions are split into two domain-swapped groups, consisting of TMs 1 and 2 from one protomer, and TMs 3′–6′ from the other protomer (Fig. 2c and Supplementary Fig. 9), resulting in exposure of the central aqueous cavity to the periplasmic side of the membrane. Compared to the outward-open structure, the structural elements of the periplasmic gate have moved toward the central two-fold axis in the inward-facing structures. Between the different inward-facing states (wide, narrow, occluded), the structural differences in this gate region are minimal, and therefore, they will be jointly described. The gate closure in the inward-facing conformation is illustrated by S123 and S123′ that approach each other to a distance of ~ 7.5 Å, while they are separated by 24.5 Å in the outward-open state (Table 1). Viewed from the periplasmic side, the gating movement of SbmA is mainly caused by the regions connecting TM 1–2 and 5–6 from both protomers. The region connecting TM 3–4 remains on the periphery, parallel to the loop of TM 5–6. The periplasmic ends of TM 2 and TM 5 are oriented to the periphery of the protein, while TM 1 and 6 are oriented toward the center of SbmA (Fig. 2c). The ends of TMs 1 and 6 from both protomers create a cap that shields the cavity from the periplasm in the inward-facing structures. The interface of the periplasmic gate is mostly lined by hydrophobic residues that form van der Waals interactions. At the center of the enclosure are L349 and L349′, whose side chains are in close proximity to each other (3.5 Å), and form the gate that closes off the central cavity.

**Fig. 3:**
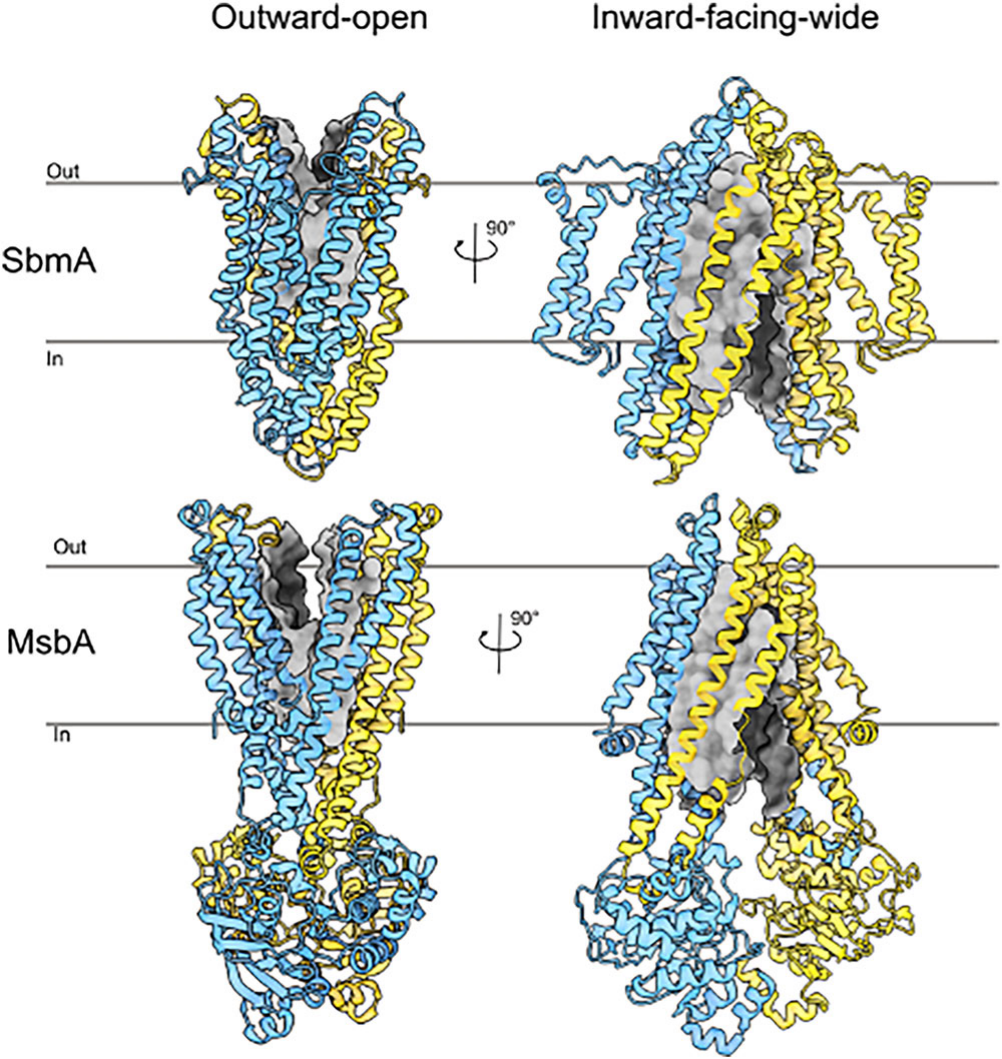
Structural similarities between SbmA and the full-length ABC-transporter MsbA. Models are shown as cartoon representations colored by chain, with the surface of the internal cavity highlighted in gray. SbmA in the outward- and inward-facing states correspond to structures in Table 1 identifiers A and G, respectively. MsbA in the outward- and inward-facing states correspond to PDB 8tso and PDB 7mew, respectively. The TMDs of SbmA and MsbA show close structural similarity in both inward-facing and outward-facing conformations, indicating a conserved structural mechanism for alternately exposing the internal cavity to either side of the membrane.

We previously noted the close structural similarity between the TMDs of SLiPT and type IV ABC transporters in their outward-open conformations^20^. The inward-facing structures presented here are also remarkably similar to the inward-facing conformations of ABC transporters (Fig. 3). The structural mechanism of alternately exposing the central cavity to either side of the membrane appears conserved between the SLiPT transporter SbmA and type IV ABC transporters. However, ABC transporters use nucleotide-binding domains (NBDs) that bind and hydrolyze ATP, releasing inorganic phosphate and ADP, while SLiPT transporters lack NBDs. Instead, SLiPT transporters may utilize the proton gradient across the membrane to drive conformational changes.

We have previously shown proton flux concomitant with substrate transport by SbmA, suggesting a role for protonatable residues in the transport mechanism^20^. The central cavity of SbmA is lined by several protonatable glutamate residues (E193, E203, E269, E276, E287, and E378) that form the glutamate ladder. Our previous mutagenesis work revealed that loss of any of the conserved glutamates rendered the transporter inactive or severely impaired of transport activity^20^. To gain insight in the residues responsible for the pH sensitivity, we carried out MD simulations where we individually protonated glutamate residues on both protomers of the glutamate ladder (Supplementary Figs. 11–13 and Supplementary Table 1). Simulations indicated that protonation of E203, E276, and E378 might contribute to stabilize the outward-open state of SbmA, while protonation of E193 triggeded a transition towards an inward-facing conformation, suggesting a role in of this residue in protonation- dependent transport.

### Conformational ensemble of SbmA in solution probed by EPR spectroscopy

**Fig. 4:**
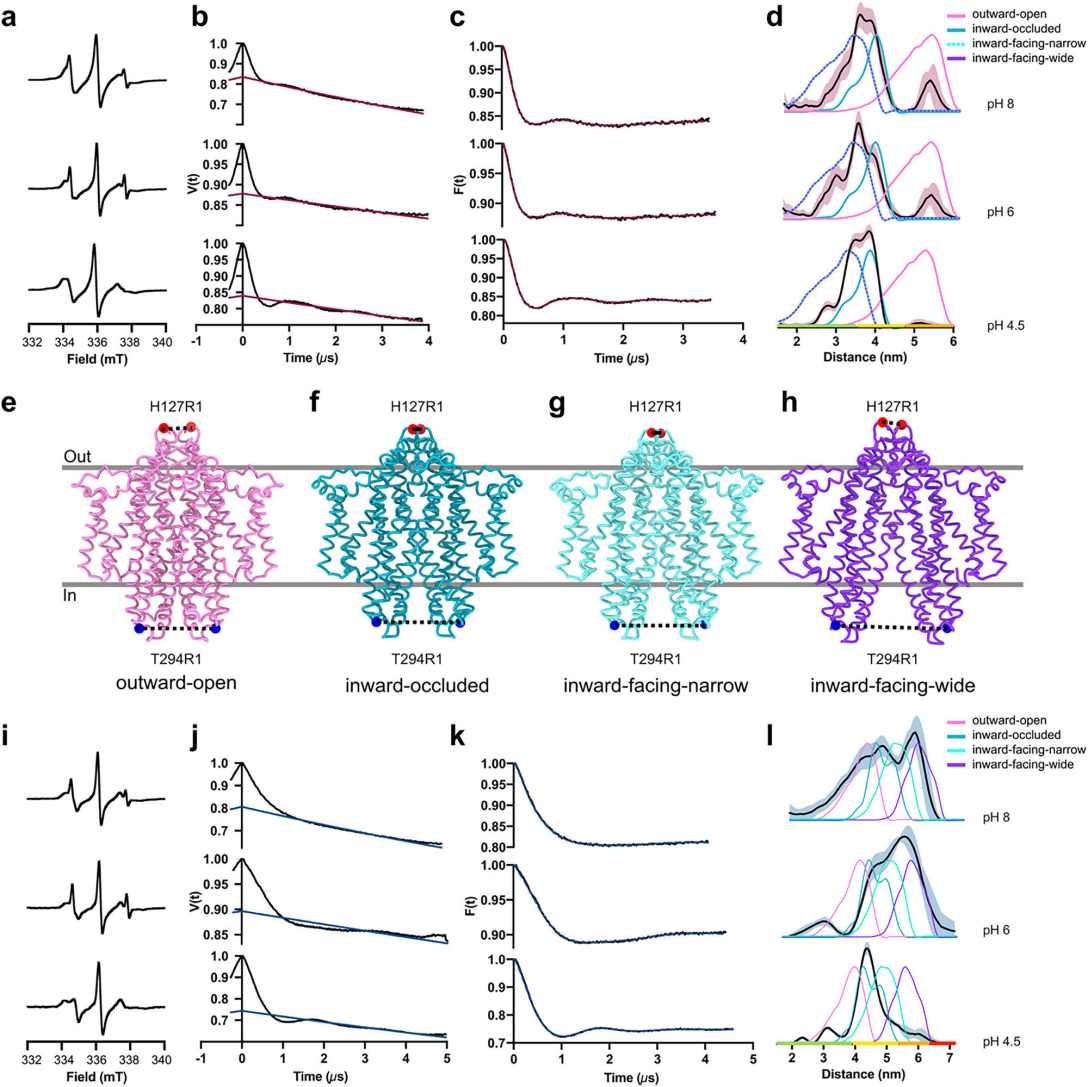
Monitoring the entire conformational ensemble of SbmA in solution by EPR spectroscopy. Top panel (**a**–**d**): EPR data for SbmA H127R1 at pH 8 (top), 6 (middle), and 4.5 (bottom). **a** CW EPR spectra showing a substantial MTSSL immobilization increase at pH 4.5, indicating stabilization of the H127 periplasmic region site. **b** PELDOR raw time-domain traces, with background shown in red. Modulations in signal amplitude enable accurate distribution and assignment of SbmA conformational states. **c** Background-corrected PELDOR time-domain traces. **d** Overlay of predicted (using MMM) distance distributions from cryo-EM structures of the outward-open (**e**, pink), inward-occluded (**f**, blue), inward-facing-narrow (**g**, cyan) and inward-facing-wide (**h**, purple) states, and experimental distance distributions from DeerAnalysis (black). Pink-shaded regions reflect distribution uncertainty from the validation analysis, with the “traffic light” system assessing distance range reliability. Middle panel (**e**–**h**): structures of the SbmA dimer in the outward-open (PDB 7p34), inward-occluded (**f**), inward-facing-narrow (**g**) and inward-facing-wide (**h**) conformations. The sites modified with MTSSL are shown in blue spheres for T294R1(cytoplasmic) and red spheres for H127R1 (periplasmic) side of the membrane. Bottom panel (**i**–**l**) EPR data for SbmA T294R1 at pH 8 (top), 6 (middle), and 4.5 (bottom). **i** CW EPR spectra showing a substantial MTSSL immobilization increase at pH 4.5, suggesting stabilization of the T294R1cytoplasmic region site. **j** PELDOR raw time-domain with background in red. Modulations enable reliable distributions and state assignment. **k** Background-corrected DPELDOR time-domain traces. **l** Overlay of predicted (using MMM) distance distributions from cryo-EM structures of the outward-open (**e**, pink), inward-occluded (**f**, blue), inward-facing-narrow (**g**, cyan) and inward-facing-wide (**h**, purple) states, and experimental distance distributions from DeerAnalysis (black). Gray-shaded regions reflect distribution uncertainty from the validation analysis, with the “traffic light” system assessing distance range reliability. Additional PELDOR data analysis using DeeLab, and yielding consistent distributions, is also presented in Supplementary Fig. 14. Source data are provided as a Source data file.

The cryo-EM reconstructions not only show considerable heterogeneity in the SbmA conformation, but also sensitivity of the conformational ensemble to the experimental conditions. To monitor the conformational ensemble of SbmA, we conducted pulsed electron double resonance experiments (PELDOR, also known as double electron-electron resonance (DEER) spectroscopy) in detergent solution (Fig. 4). We introduced a methanethiosulfonate spin label (MTSSL) by covalent linkage to an engineered single cysteine residue in SbmA to allow for the measurement of the inter-spin distance between the labeled residues in the two protomers of the homodimeric protein. Based on the structural models, we constructed single cysteine mutants that are suitable to report on distance changes associated with gate opening and closure on the cytoplasmic and periplasmic sides. In the periplasmic gate, we replaced the wild-type histidine at position 127 with an MTSSL-modified cysteine (the resulting modified mutant is termed H127R1). Similarly, to probe the state of the cytoplasmic gate, we replaced threonine at position 294 by an MTSSL-modified cysteine (T294R1).

In-silico tools consistently predicted distinct distance distributions for the inward-facing-narrow, inward-facing-wide, inward-occluded and outward-open conformations (Fig. 4e–h). For the periplasmic H127R1, the predicted distances between the spins for the inward-facing-wide and inward-facing-narrow states are identical (centered around a mean distance of ~33 Å), while for the inward-occluded state, the prediction shows a distinct distance distribution centered ~40 Å and for the outward-open state ~50 Å. For the spin label pair on the SbmA cytoplasmic side (T294R1), the predicted mean distances of the distributions are ~40, 43, 50, and 56 Å for the outward-open, inward-occluded, inward-facing-narrow and inward-facing-wide states, respectively (Fig. 4d, l). The well-separated distance predictions allowed for experimental assessment of the conformational ensemble in different conditions in solution.

We collected PELDOR data at three pH values (pH 8, pH 6, and pH 4.5). In all PELDOR experiments, we observed strong visual oscillations in the raw (background uncorrected) data traces, indicating high reliability of the resulting distance distributions (Fig. 4b, c, j, k, and Supplementary Fig. 14a, c). In addition to the DeerAnalysis results presented in Fig. 4, we also performed analysis using DeerLab, with the corresponding results presented in Supplementary Fig. 14. Analyses with both methods yielded consistent results, confirming the reliability of the PELDOR distance distributions. The main distance components obtained for H127R1 are consistent with the in-silico predictions for the three inward-facing states, which together constitute the main species present in the SbmA ensemble at all three pH values tested (Fig. 4d and Supplementary Fig. 14b). At pH 6, and even more at pH 8, main distance components consistent with the outward open SbmA state are also present, although the PELDOR data suggest that these species may only constitute a minor population within the SbmA ensemble (Fig. 4d). The presence of a major population of inward-facing and a minor population of outward-open conformations informed by PELDOR in solution is consistent with the cryo-EM data (Table 1 and Fig. 2).

With the spin labels on the cytoplasmic side (T294R1) at pH 8, the ensemble consists of all SbmA states, where the distance distribution is broad with peak shoulders relating to the predicted distances for both inward-facing-wide and inward-occluded (Fig. 4l and Supplementary Fig. 14d). In the same distribution, there are also shorter distance components present, which are consistent with the outward-open state. At pH 6, there is a slightly lower probability for the shorter distances than at pH 8, suggesting the outward-open population decreases with the drop in the pH (Fig. 4l). At pH 4.5, the PELDOR data for T294R1 are distinctly different compared to the higher pH data (Fig. 4l). At pH 4.5, the narrow distance distribution is most consistent with the inward-occluded state (Fig. 4l). These species dominates the ensemble, to which the inward-facing-wide state seems to have fully converted (Fig. 4l).

To assess the local environment and mobility of H127R1 and T294R1, located on either side of the membrane, we also recorded continuous wave (CW) EPR spectra at room temperature at different pH values (Fig. 4a, i). The CW EPR spectral lines showed large differences when recorded at different pH values, indicating that both H127R1 and T294R1 become highly rigid at pH 4.5 compared to pH 8 and 6. Collectively, the CW EPR and PELDOR data suggests that acidic pH rigidifies both peri- and cytoplasmic gate regions of SbmA and pushes the SbmA conformational ensemble towards the inward-occluded state.

## Discussion

We have previously reported the close structural similarity between SLiPT and type IV ABC transporters^20^, based on their outward-open conformations. In this study, we show that the conformational changes, outward-open to inward-open state, of SLiPT transporters like SbmA are also similar to those in the membrane domains of type IV ABC transporters (Fig. 3). The membrane domains of the type IV ABC transporter MsbA (lipid A flippase) and SbmA in the inward-facing conformation can be superimposed with an RMSD of 1.34 Å (TMD residues, Fig. 3). Despite the two proteins having very low sequence similarity (less than 20%), structurally they are very similar. Additionally, the structural heterogeneity of the inward-open state in SbmA (Table 1 and Fig. 2) is consistent with the different extents of opening on the cytoplasmic side of ABC transporters^29,30^. The phylogenetic analyses suggest that SLiPTs have evolved from an early ancestor of the YddA family of ABC transporters (of unknown function) that are conserved amongst Gram-negative bacteria and mycobacteria. YddA-like proteins are evolutionary distant from the other type IV ABC transporters (MsbA, YojI, and MdlA/B). The closest structural homolog of SLiPTs and YddA is the Rv1819c (cobalamin transporter) from *M. tuberculosi*s that is a full-length ABC transporter. We therefore propose that, despite being secondary transporters, SLiPTs have originated from the full-length ABC transporters that have lost the NBDs and the coupling helices that connect the TMDs to the NBDs in ABC transporters. Instead, SLiPTs contain a glutamate ladder, which allows for conformational changes between inward and outward-facing states by protonation and deprotonation. Interestingly, the YddA sequence contains some of the conserved glutamate residues that are found in the SbmA glutamate ladder. The presence of this incomplete glutamate ladder in YddA, without the equivalents of E203 and E287, further supports our theory on the evolutionary origin of SLiPTs (Supplementary Fig. 10b). The additional TM0 domain that SLiPTs have acquired does not undergo any conformational changes when the conformation switches between out- and inward-facing states. It may act as a scaffold for stabilizing the TMD within the inner membrane, which may be needed because SLiPTs do not contain the characteristic elbow helices of ABC transporters.

Along the transport cycle, the global conformational changes in SbmA are very similar to those in the type IV ABC transporters but facilitated by protonation and deprotonation rather than by ATP binding, hydrolysis, and release of ADP and phosphate. Cryo-EM structures, EPR, and MD simulations data show how protonation shifts the conformational equilibrium. In line with the role of SbmA in importing peptides, the protein samples the outward-facing conformation sufficiently frequently so that transported substrates can access the cavity for internalization. Upon proton binding and movement along the glutamate ladder, the cytoplasmic gate breaks and destabilizes the outward-facing conformation. A periplasmic gate forms, mostly driven by van-der-Waals interactions, and subsequently the cavity opens towards the cytoplasmic side of the inner membrane. EPR, cryo-EM, and MD simulations data also show intermediate states, suggesting conformational plasticity in SbmA. The plasticity is further evidenced from the sensitivity of the conformational ensemble to slight differences in the experimental conditions of the cryo-EM experiments, including pH, bilayer replacing system (detergent micelle or lipid nanodiscs) or the lipid composition of the nanodiscs. Although these data describe the global changes upon protonation, they do not directly address the mechanics by which protonation of the glutamate ladder shifts the transporter from an outward-facing to an inward-facing conformation. We have previously shown that the loss of a single glutamate residue from the ladder can either render the transporter inactive or result in slower transport kinetics dependent on the substrate^20^. The work presented here can partially but not fully explain these observations. The glutamate ladder is separated by the substrate binding cavity by the central gate formed by Y368/Y368′. E203 and E378 are the first two glutamates after the internal gate, and MD simulations suggests that their protonation stabilizes the outward-open conformation rather than converting the structural ensemble towards inward-facing states. It is very likely that the role of these glutamates is to either prime/stabilize the TMD for substrate binding or facilitate proton movement without causing structural changes. This hypothesis is supported by the EPR data that show plasticity within the TMD that is becoming more rigid upon protonation. E269 does not impact the TMD, and it may act as a relay for proton movement along the ladder. On the other hand, the protonated E193 seems to acts as the switch to trigger the transition of the TMD towards an inward-facing conformation. In the outward-open conformation, E193 is located within the interface of the cytoplasmic gate, and it is forming a hydrogen bond with R180′ and E193′. It is possible that protonation results in the destabilization of these interactions and transition toward the inward-facing state. In the inward-facing structures, E193 is separated from E193′ and R180′ because of the movement of TM3 away from TM3′. This is also apparent in the EPR data that shows stabilization of the inward-facing conformation upon protonation of SbmA. The role of a glutamate to facilitate conformational changes within the cytoplasmic gate has also been reported for the microcin export ABC transporter McjD. In McjD, the cytoplasmic gate is formed by E309 and R141, forming a salt bridge, and upon its disruption, there is significant loss of transport activity. Therefore, SLiPTs and ABC transporters appear to have several mechanistic commonalities.

Based on the structures, EPR data and MD simulations, we propose a mechanism for the internalization of peptides across the inner membrane by SLiPTs. In the absence of a substrate, SbmA probes both inward- and outward-open conformations. Based on the MD simulations, we propose that a proton binds first at E203, and it is very likely to move along E203, E269, E276, and E378 to reduce the structural plasticity of the TMD and facilitate substrate binding. Substrate binding results in the movement of TMs 1/2 towards TMs 1′/2′ and the formation of a closed periplasmic gate. This rearrangement, together with the movement of the proton at E193, results in the disruption of the cytoplasmic gate, movement of TMs 4–5 away from TMs 3′/6′, disruption of the glutamate ladder and release of the proton to the cytosol. TM6a/b also adopts a straighter conformation to facilitate the release of the substrate from the cavity. Due to the plasticity of the TMD and the weak nature of the periplasmic gate, the transporter reverts to sample an outward-open conformation again.

## Methods

### Evolutionary analysis of SbmA

We extracted protein sequences of ≥300aa from Gram-negative bacteria from UniprotKB that were annotated as “SbmA” or “YddA”. A further 252 proteins from the BclA (BacA-like) family were obtained from the Uniref cluster uniref_cluster_50:UniRef50_K8P3L1. We also ensured that all 366 protein sequences from a recent study^26^ that classified SbmA and closely-related proteins were also included (except three with ≤300aa), requiring the addition of a further 273 sequences. The MsbA, YojI, MdlA and MdlB protein sequences from the *E. coli* strain K12 were also included in the analysis.

We used the tool, DeepTMHMM v1.0^31^ to infer the location of the TM domain in each protein sequence, using the coordinates of the first and last predicted TM helices. The predicted TM domains were extracted from all protein sequences and aligned using Clustal-Omega v1.2.4^32^. ModelTest-NG v0.2.0^27^ was used to determine the best-fitting evolutionary model for the alignment. A maximum-likelihood phylogenetic tree was generated using RAxML-NG v1.2.0^33^ using the LG + G4 model with 100 bootstrap replicates. The tree was visualized together with metadata and associated bootstrap values using Microreact^34^.

### Strains and culture conditions

SbmA was cloned into the pBXC3H vector using the FX cloning method^35^ from genomic DNA and expressed in the *E. coli* MC1061 strain. The bacterial culture was grown in Luria-Bertani (LB) broth containing 100 μg ml^−1^ ampicillin at 37 °C with continuous shaking at 190 rpm. When an optical density of 0.7 was reached, the culture was induced by adding 10^−4^% (w/v) L-arabinose. After 2 h, cells were harvested by centrifugation (20 min, 6000 × *g*, 4 °C) and washed twice with ice-cold TBS buffer (20 mM Tris-HCl, pH 7.4, 120 mM NaCl). The cells were resuspended in TBS containing 2 mM MgSO_4_ and 4 µg ml^−1^ DNAse I before storage at −70 °C.

### Purification of SbmA

Purification of SbmA for the SbmA-Fab complex and EPR experiments was described before^20^. To purify SbmA for the 200 kV structures described in this paper, cells were lysed by three passages through a high-pressure homogenizer HPL6 system (Maximator) at 30 kpsi. Cell debris was removed by subjecting the broken cells to centrifugation (20 min, 30,000 × *g*, 4 °C), after which the crude membrane fraction was separated by ultracentrifugation (90 min, 194,000 × *g*, 4 °C). The membranes were homogenized in TBS buffer and stored at −70 °C after flash freezing in liquid nitrogen. After thawing, homogenized membranes were solubilized in solubilization buffer (TBS, 1% (w/v) DDM) for 1 h at 4 °C with constant agitation. The non-solubilized fraction was separated by ultracentrifugation (25 min, 444,000 × *g*, 4 °C). The supernatant was transferred to a polypropylene column (Biorad) filled with 0.5 ml equilibrated Nickel Sepharose 6 Fast Flow beads (Cytiva) and incubated for 1 h at 4 °C with constant agitation. The unbound protein was allowed to run through, and the column was washed with 20 CV wash buffer (TBS, 0.05% (v/w) DDM, 60 mM imidazole, pH 7.4). Bound protein was eluted in three fractions (350, 850, and 500 µl) with elution buffer (TBS, 0.05% (w/v) DDM, 500 mM imidazole, pH 7.4). The main fraction was subjected to further purification by size-exclusion chromatography (SEC) using Superdex 200 Increase 10/300 GL column (Cytiva). Depending on the conditions, the following SEC buffer compositions were used: for the sybody-bound and nanodiscs structures (Table 1 C, D, and F), TBS at pH 7.4 and 0.05% (w/v) DDM, and for the detergent structure (Table 1 E), TBS at pH 8.0 and 0.05% (w/v) DDM. Peak fractions were pooled together, concentrated to approximately 6 mg ml^−1^ using a 100-kDa molecular weight cut-off (MWCO) centrifugal concentrator (Millipore), and used immediately for grid preparation. In the case of the nanodisc structure, the pooled peak fraction was immediately used for nanodisc reconstitution.

### Sybody generation and complexation

Sybodies were generated as described by Zimmermann et al.^28^ and raised against biotinylated SbmA_T403C_. From the resulting sybodies, Sybody2 (SB2) was selected, expressed and purified according to established protocols^28^. Purified SbmA was incubated with SB2 in a 1:2 molar ratio for 30 min at 4 °C with gentle mixing. The SbmA-SB2 complex was further purified by SEC with TBS at pH 7.4 and 0.05% (w/v) DDM using a Superdex 200 Increase 10/300 GL column. Peak fractions were pooled together, concentrated to approximately 6 mg ml^−1^ using a 100-kDa MWCO centrifugal concentrator and used immediately for grid preparation.

### Nanodisc reconstitution

SbmA-Fab complex—the plasmid for the expression MSP1E3D1 was a gift from *S. Sligar* (Addgene plasmid #20066)^36^. The reconstitution of SbmA in complex with Fab S11-1 into lipid nanodiscs was described previously^20^, with several adjustments. The DOPG in the lipid mix was replaced with dioleoylphosphocholine DOPC (final composition DOPC: POPG: POPO-cardiolipin 13:4:1), as the DOPG-based nanodiscs were not stable in low pH. Additionally, the sample was further purified by SEC with a low pH buffer containing 20 mM MES, pH 5.5 and 300 KCl using a Superdex 200 Increase 10/300 GL column. Peak fractions were pooled together, concentrated to approximately 5 mg ml^−1^ using a 100-kDa MWCO centrifugal concentrator and used immediately for grid preparation.

SbmA alone—a lipid mixture containing *E. coli* polar lipid extract (Avanti Polar Lipids) and egg phosphatidylcholine (Avanti Polar Lipids) was prepared at a 3:1 ratio in 50 mM KPi, pH 7.5 and solubilized at total lipid concentration of 10 mg ml^−1^ in 1.75% (w/v) DDM. Purified SbmA was mixed with purified MSP1E3D1 and solubilized lipids at a 1:5:500 molar ratio, and incubated on ice for 30 min. Detergent was removed by adding 200 mg ml^−1^ bio-beads SM-2 (Biorad) and incubating for 30 min at 4 °C. The sample was incubated overnight with a second addition of 200 mg ml^−1^ bio-beads at 4 °C with constant agitation. Bio-Beads were separated from the protein sample, with the latter incubated with 0.5 ml Ni-Sepharose beads for 1 h at 4 °C with constant agitation. The column was washed with 20 CVs TBS buffer, pH 7.4 to remove unbound protein and empty nanodiscs. Bound proteins were eluted in 3 fractions (350, 850, and 500 µl) using TBS buffer, pH 7.4, containing 500 mM imidazole, pH 7.4. The main fraction was subjected to further purification by SEC using Superdex 200 Increase 10/300 GL column with 20 mM MES, pH 6.5 and 120 mM NaCl as buffer. Peak fractions were pooled together, concentrated to approximately 6.5 mg ml^−1^ using a 100-kDa MWCO centrifugal concentrator and used immediately for grid preparation.

### Cryo-EM sample vitrification and data acquisition

For the inward-facing-narrow and inward-facing-wide conformations of SbmA-Fab in lipid nanodiscs at low pH, 4 μl of reconstituted complexes were applied to glow-discharged (Leica, 60 s/8 mA) Quantifoil holey carbon grids (R1.2/1.3, 300 copper mesh). After 30 s of incubation with 95% chamber humidity at 10 °C, the grids were blotted for 6 s and plunge-frozen in liquid ethane using a Vitrobot Mark IV (FEI). CryoEM data were collected at the Polish National cryo-EM facility, SOLARIS, on Titan Krios G3i microscope (Thermo Fisher Scientific) operated at 300 kV and nominal magnification of ×105,000. Movie frames were collected at the calibrated physical pixel size of 0.86 Å per pixel with a defocus range of −2.7 to −0.9 μm. Movies were recorded in counting mode on a Gatan K3 direct electron detector equipped with GIF BIO Quantum energy filter operated with a slit width of 20-eV, and saved as gain-corrected TIFF files using EPU version 2.10.0.5 REL. Dose rate over vacuum was 17.05 e^−^ px^−1^ s^−1^, and exposure time was set to generate a total dose of 42.39 electrons per Å^2^. For all other structures, 2.7 µL of samples were applied onto glow-discharged (Edwards Scancoat 6, 45 s at 5 mA) Quantifoil holey carbon grids Au R1.2/1.3 300-mesh), blotted for 4 s using a Vitrobot Mark IV (Thermo Fisher Scientific) at 15 °C and 100% humidity, and subsequently plunge frozen in a liquid ethane-propane mixture and stored in liquid nitrogen until use. The remaining datasets were collected at the in-house cryo-EM facility of the University of Groningen on a Talos Arctica cryo-TEM (Thermo Fisher Scientific) operating at 200 keV with a BioQuantum post-column energy filter (Gatan) in zero-loss mode and a 20-eV slit width, and a 100 µm objective aperture on a K2 Summit direct electron detector (Gatan) in counting mode. Movies were recorded in an automated fashion with SerialEM version 3.9.0 beta using a 3 × 3 multi-shot array^37,38^, at a physical pixel size of 1.022 Å (calibrated magnification of ×48,924, nominal magnification ×130,000), a defocus range from −0.8 to −1.8 μm, an exposure time of 9 s with a subframe exposure time of 150 ms (60 frames), and a total electron exposure on the specimen level of 50.1 electrons per Å^2^. Optimal regions for data acquisition on the grid were screened and selected through an in-house written ice thickness measurement script implemented in SerialEM^39^, which was set between 30 and 50 nm as described previously^40^. The data quality was monitored on-the-fly using the software FOCUS version 1.1.0^41^.

### Cryo-EM image processing

For the 200-kV cryo-EM datasets, all movies from each dataset were subjected to motion correction and dose weighting of frames in MotionCor2 version 1.4.0^42^. The CTF parameters were estimated on the movie frames with CTFFIND4.1.14^43^. Low-quality images were removed on the basis of visual inspection of the images showing contamination and poor CTF estimation, and duplicate exposures were removed using an in-house written script. The remaining images were used for further processing. Motioncor2 and CTFFIND4 were executed within the FOCUS software. Particles were picked with crYOLO version 1.7.6^44,45^ using the PhosaurusNet architecture with anchors set to 140. Particles were picked on all micrographs using the general network model with a relaxed threshold of 0.2, extracted with imported particle coordinates from crYOLO in RELION version 3.1.3^46^, and subsequently imported into cryoSPARC version 3.3.2 for further processing^47^. Bayesian polishing^48^ was performed in RELION. The UCSF PyEM collection of Python scripts^49^ was used to convert cryoSPARC output files to STAR files for import into RELION (csparc2star.py) and to create Euler angle distribution plots (star2bild.py). All reported resolutions were estimated with the 0.143 cut-off criterion^50^ with gold-standard Fourier shell correlation (GS-FSC) between two independently refined half maps^51^. During sharpening, high-resolution noise substitution was used to correct for convolution effects of real-space masking on the FSC curve^52^. The directional resolution anisotropy of density maps was quantitatively evaluated using the remote 3DFSC processing server (accessed at https://3dfsc.salk.edu)^53^.

Inward-facing-narrow and inward-facing-wide conformations of SbmA-Fab in lipid nanodiscs at low pH—all processing was done in cryoSPARC version 4.2.1. A total of 6069 movies were motion- and CTF-corrected in patch mode, and 5901 micrographs were selected for further analysis. Particles were picked with the cryoSPARC template picker, resulting in 1,588,637 picked particles. 2 × 2 binned particles were subjected to two rounds of 2D classification (50 iterations, 20 full iterations, batch size 200, uncertainty factor 5). Cleaned particles showing protein secondary structure elements (390,618) underwent 3D classification with three classes (ab initio). This resulted in the separation of the low-resolution class corresponding to the minor population of outward-open particles (56,954, ~15%) and two classes with high-resolution features corresponding to the “wide” (176,498) and “narrow” (157,166) inward-open conformations of SbmA that were separately refined. Particles corresponding to each dataset were re-extracted unbinned at 0.86 Å pix^−1^ and refined, accounting for local defocus refinement, resulting in 3.18 and 3.38 Å resolution maps, respectively. These reconstructions that still displayed heterogeneity, particularly TM8, were further analyzed by means of 3D classification without alignment (3 classes, batch size 10 000, PCA mode). This resulted in four final maps representing the “inward-facing-wide” (3.25 Å resolution, 63,225 particles), “inward-facing-intermediate” (3.45 Å resolution, 56,582 particles), “inward-facing-intermediate” (3.49 Å resolution, 54,872) and “inward-facing-narrow” conformations (3.6 Å resolution, 50,775). The final cryo-EM data processing workflow and validation are summarized in Supplementary Figs. 1 and 2, and the data collection and processing parameters are summarized in Supplementary Table 2.

Inward-facing-narrow and inward-facing-wide conformations of SbmA-Sybody in detergent solution at high pH—a total of 1676 movies were recorded with SerialEM from a single grid. After manual curation, 1585 movies remained for further processing. Particles were picked, and a total of 750,685 extracted particles with a box size of 256 pixels were subjected to a single round of 2D classification. A total of 333,854 particles underwent a heterogenous refinement step with three ab initio reconstructions as input without imposed symmetry. The best class containing 231,986 particles was subjected to a round of nonuniform refinement with imposed C2 symmetry, resulting in a 3.3 Å resolution reconstruction. The map quality was improved by local and global CTF refinement steps on particle defocus and optics group, respectively. An improvement was obtained by a Bayesian polishing step, resulting in a reconstruction at 3.2 Å resolution. A single heterogenous refinement step with five Ab Initio reconstructions as input without imposed symmetry was performed, which resulted in three distinct classes containing 34,724, 71,122, and 114,603 particles. The two classes containing 71,122 and 114,603 particles represented inward-facing-narrow and inward-facing-wide conformations, respectively, with each SbmA protomer bound to a sybody. Both were subjected independently to a round of nonuniform refinement with imposed C2 symmetry and a global CTF refinement steps on the optics group, followed by a final round of nonuniform refinement with imposed C2 symmetry. A reconstruction of the sybody-bound inward-facing-narrow conformation was obtained at 3.24 Å resolution with a global B-factor of −104.6 Å^2^, and a sphericity value reported by 3DFSC of 0.988 out of 1. A reconstruction of the sybody-bound inward-facing-wide conformation was obtained at 3.14 Å resolution with a global B-factor of −107.4 Å^2^, and a sphericity value reported by 3DFSC of 0.984 out of 1. In the class with 34,724 particles, SbmA was bound to only a single sybody representing an inward-facing-wide conformation and was subjected to a final round of nonuniform refinement without imposed symmetry, resulting in a reconstruction at 3.96 Å resolution with a global B-factor of −107.9 Å^2^, and a sphericity value reported by 3DFSC of 0.641 out of 1. The final cryo-EM data processing workflow and validation are summarized in Supplementary Figs. 3–5, and the data collection and processing parameters are summarized in Supplementary Table 3.

Inward-facing-wide conformation of SbmA-Bac7 in detergent solution at high pH—a total of 1620 movies were recorded with SerialEM from a single grid. After manual curation, 847 movies remained for further processing. Particles were picked, and a total of 357,486 extracted particles with a box size of 256 pixels were subjected to a single round of 2D classification. A total of 160,862 particles underwent a heterogenous refinement step with three ab initio reconstructions as input without imposed symmetry. The best class containing 99,732 particles was subjected to a round of nonuniform refinement with imposed C2 symmetry, resulting in a 3.5 Å resolution reconstruction. The map was improved to 3.4 Å resolution with a Bayesian polishing step. A final round of nonuniform refinement with imposed C2 symmetry after global CTF refinement based on optics group resulted in a reconstruction at 3.36 Å resolution from 99,732 particles with a global B-factor of −126.5 Å^2^, and a sphericity value reported by 3DFSC of 0.983 out of 1. The final cryo-EM data processing workflow is summarized in Supplementary Fig. 6, and the data collection and processing parameters are summarized in Supplementary Table 3.

Inward-facing-occluded conformation of SbmA in lipid nanodiscs at low pH—a total of 1933 movies were recorded with SerialEM from a single grid. After manual curation, 1295 movies remained for further processing. Particles were picked, and a total of 804,212 extracted particles with a box size of 256 pixels were subjected to a single round of 2D classification. A total of 503,455 particles underwent a heterogenous refinement step with three Ab Initio reconstructions as input without imposed symmetry. The best class containing 192,390 particles was subjected to a round of nonuniform refinement with imposed C2 symmetry, resulting in a 3.4 Å resolution reconstruction. The map was improved to 3.3 Å resolution by global CTF refinement based on the optics group. A further improvement was obtained by a Bayesian polishing step, resulting in a reconstruction at 3.2 Å resolution. A single heterogenous refinement step with three ab initio reconstructions as input without imposed symmetry was performed to remove bad particles. A final round of nonuniform refinement with imposed C2 symmetry resulted in a reconstruction at 3.07 Å resolution from 146,295 particles with a global B-factor of −117.4 Å^2^, and a sphericity value reported by 3DFSC of 0.983 out of 1. The final cryo-EM data processing workflow is summarized in Supplementary Fig. 7, and the data collection and processing parameters are summarized in Supplementary Table 3.

### Model building and validation

Initial models were built using ModelAngelo version 0.2^54^, and subsequently inspected and modified in COOT version 0.9.8.1^55^. All models were subjected to an iterative process of real-space refinement against the cryoSPARC-sharpened map in PHENIX version 1.20.1-4487^56,57^. All programs used for image processing of the 200-kV cryo-EM data were managed through the SBGrid software package tool^58^. The chemo-physical properties of the final models were validated with MolProbity^59^. Refinement parameters are summarized in Supplementary Tables 1 and 2.

### EPR spectroscopy

Sample preparation—SbmA was purified in either a low pH (100 mM citric acid, pH 4.5, 300 mM NaCl, 0.06% (w/v) DDM) or high pH (100 mM TES-NaOH, pH 8.0, 300 mM NaCl, 0.06% (w/v) DDM) buffer and labeled as previously described^20,60^. Samples for EPR were prepared as previously described^61,62^. Briefly, 13 µl of purified spin-labeled protein was diluted in 27 µl of the appropriate buffer. To prepare a sample at pH 6.0, 13 µl purified protein at pH 8.0 was added to 27 µl of lower pH buffer, until the desired pH 6.0 value was reached. The protein concentration before dilution ranged from 14.4 to 33.0 mg ml^−1^. All CW EPR samples were measured at room temperature. Samples for PELDOR spectroscopy were prepared as previously described^63^. In brief, 40% (v/v) ethylene glycol was added to the sample and mixed well by pipetting, then the sample was transferred to Quartz 3 mm EPR tubes and snap-frozen in liquid nitrogen. We utilized PELDOR to investigate conformational changes under various conditions. A nitroxide site-directed spin label, MTSSL, served as a paramagnetic center.

PELDOR (or DEER) set up and distance measurements—all pulsed and PELDOR distance measurements have been conducted on a BRUKER ELEXSYS E580 pulsed spectrometer operating at Q-band (34 GHZ) with a 150 W TWT Q-band amplifier and a probe head supporting a cylindrical resonator ER 5106QT-2w. A second BRUKER 400U microwave source was used for PELDOR measurements. The spectrometer is equipped with a cryogen-free variable temperature cryostat (CF-VTC) from Cryogenic Ltd. All measurements reported here were performed at 50 K, with typically a sample volume ranging from 60 to 100 μL. The resonator was systematically overcoupled during all the pulsed experiments, and the Q-factor was maintained approximately the same for all measurements. The PELDOR experiments were carried out using the standard dead-time free four-pulse sequence^64^:

*π*/2(probe)- *t*1-*π* (probe)-*t*-*π* (pump)- *t*1 + *t*2-*t*-*π* (probe)-*t*2-echo

where all the pulses are rectangular. The refocused echo intensity was monitored as a function of *t*. The pump pulse was applied at the maximum of the ED-FS spectrum, whereas the probe frequency was set at 80 MHz offset from the pump frequency. The probe *p*/2 and *p* pulse lengths were 16 and 32 ns, respectively. The *p* pump pulse length was 12 ns, short enough for maximizing the modulation depth and keeping both the probe and pump excitation bandwidths well separated. The interpulse *t*_1_ was set to 380 ns, whereas *t*_2_ was adjusted according to the strength of the refocused echo signal and to be long enough to cover the expected distances. The SRT was 3 ms for all PELDOR experiments, and the averaging times were typically between 2 up to 24 h until a sufficient signal-to-noise is achieved. A 2-step phase cycling was used on the first probe pulse to remove any receiver offsets that might be introduced during the signal acquisition. The interpulse *t*1 in the probe sequence was stepped sixteen times, starting from its initial value 380 ns by 8 ns and the corresponding time traces were added together to average out the ESEEM deuteron effects, which in some cases could be quite prominent in the time traces. These are typical values commonly used at Q-band for *t*1 averaging to remove deuterium modulation effects during the data acquisition.

PELDOR data analysis-DeerAnalysis: data were processed using DeerAnalysis2022^65^ according to PELDOR/DEER guidelines^66^. The data were loaded, the 2 + 1 artefact^67^ truncated from the dataset (−600 ns), then phase and background adjusted using the “!” automated adjustment. The background corrected traces were then transformed from the time domain to the distance domain using Tikhonov Regularization^68^, and the value given by the L-curve. The resultant background correction was then validated using a module for validation implemented in DA. The validation was carried out after initial Tikhonov regularization, varying the background start time from 5% to 80% of the respective time windows of the cut data for 16 trials. From this, the raw data were re-loaded and processed (Tikhonov regularization) with the cutoff and background start time as established from the first round of validation. The resulting data are taken for plotting figures showing raw data/background and form factor/fit. This is the starting point for a full validation, where the background start time was again varied from 5 to 80% of the time window for 16 trials, as well as some added “white noise” with a level of 1.50 for 50 trials. The result of validation was pruned and used for plotting the distance distribution and confidence interval. While DeerAnalysis provided the standard analysis framework, we additionally employed the Python-based DeerLab software^69,70^, which offers a more user-independent approach to data interpretation. This dual analysis allowed us to validate our results consistently and to accurately account for the potential contribution of the “2 + 1” signal component^67^.

In-silico modeling—the structures for the inward closed and open conformations were loaded to MMM 2021.1 (a Matlab plugin–Matlab R2023a was used)^71^. The H127 and T294 positions of monomer A and B were selected, and then these positions were site-scanned/selective residues. The rotamers produced were for MTSSL at ambient (298 K) temperature. This produces rotamers for the sites, which are then attached using the attach precomputed rotamers option before producing the PELDOR predictions.

 CW EPR spectroscopy—CW EPR experiments were performed on a Bruker Magnettech ESR5000 X-band. The spin-labeled sample was loaded into an EPR tube before the addition of ethylene glycol-d6 using a Gilson pipette. The samples were measured at room temperature. The measurement conditions used were: 330–345 mT magnetic field, 60 s sweep time, 0.1 mT modulation, 100 kHz frequency and 10 mW (10 dB) microwave power.

### Reporting summary

Further information on research design is available in the Nature Portfolio Reporting Summary linked to this article.

## Supplementary information

Supplementary Information

Reporting Summary

Transparent Peer Review File

## Source data

Source Data

## Data Availability

An interactive version of the phylogenetic tree of SbmA and related ABC transporter proteins is available via Microreact: https://microreact.org/project/sbma. Cryo-EM density maps, half maps, and masks have been deposited in the Electron Microscopy Data Bank (EMDB); SbmA-Fab inward-facing-wide EMD-51036, SbmA-Fab inward-facing-narrow EMD-51037, SbmA-Sb2 inward-facing-narrow with 2 Sb2 EMD-50994, SbmA-Sb2 inward-facing-wide with 1 Sb2 EMD-50995, SbmA-Sb2 inward-facing-wide with 2 Sb2 EMD-50996, SbmA inward-facing-wide EMD-50997, SbmA inward-facing-occluded EMD-50998. The atomic coordinates have been deposited in the Protein Data Bank (PDB) under accession codes 9G4E (SbmA-Fab, inward-facing-wide), 9G4F (SbmA-Fab, inward-facing-narrow), 9G3D (SbmA-Sb2, inward-facing-narrow with 2 Sb2), 9G3E (SbmA-Sb2, inward-facing-wide with 2 Sb2), 9G3F (SbmA, inward-facing-wide), and 9G3G (SbmA, inward-facing-occluded). Raw movies have been deposited in the Electron Microscopy Public Image Archive (EMPIAR) under accession numbers EMPIAR-12192 (SbmA-Fab, inward facing wide and inward-facing-narrow), EMPIAR-12588 (SbmA-Sb2), EMPIAR-12589 (SbmA, inward-facing-wide) and EMPIAR-12590 (inward-facing-occluded). The previously resolved structure of SbmA in the outward-open conformation and the structures of MsbA in the outward- and inward-facing conformation used in this study is available through the PDB under the accession codes 7P34, 8TSO, and 7MEW, respectively. The AlphaFold model of YddA used in this study is available through the AlphaFold Protein Structure Database under the accession code AF-P31826-F1. All starting systems used in MD simulations, the simulation parameters, trajectory data and analysis plots are available at Zenodo with ID 17544057). Source data are provided with this paper.
