# Supplementary material for: Shared structural mechanisms of alternating access between the secondary peptide transporter SbmA and ABC transporters
Source: Nat Commun. 2026 Apr 15;17:5619. doi: 10.1038/s41467-026-71633-3 (PMC13316013; doi:10.1038/s41467-026-71633-3)
Supplement: Supplementary file 1 — Supplementary Information [file 41467_2026_71633_MOESM1_ESM.pdf]

Supplementary information

Shared structural mechanisms of alternating access between the secondary peptide transporter SbmA and ABC transporters

Supplementary Materials and Methods

MD Simulations

To gain an equilibrated membrane around the protein, the cryo-EM structure of SbmA was simulated in a membrane containing 75% POPE, 20% POPG, and 5% cardiolipin (CDL2) in a coarse-grained representation for 1 µs. First, the dimeric cryo-EM structure of SbmA was converted to a coarse-grained representation of the Martini force field using the martinize.py script [1, 2]. The insane.py script was then used to randomly place PE, PG, and CDL2 around SbmA. For the outward-open conformation a box of size 11.0 x 11.0 x 11.0 nm was used. For the inward-open-wide system, a larger box size of 12.5 x 12.5 x 11.0 nm was used, due to the conformational change in SbmA of the inward conformation resulting in an increase in distance between the TM0b helices. Both systems were then solvated, neutralized, and NaCl was added to a final concentration of 150 mM. A total of 1 µs was simulated with a time step of 20 fs. Neighbor searching was performed every 20 steps using the Verlet cutoff scheme. The Reaction-Field algorithm [3] was used for electrostatic interactions with a cutoff of 1.1 nm. A single cut-off of 1.1 nm was used for van der Waals interactions. Temperature coupling was done with the V-rescale algorithm [4] and pressure coupling was done with the Parrinello-Rahman algorithm [5]. During the production run, position restraints were applied to backbone and side chain atoms with a force constant of 100 kJ mol^{-1} nm^{-2}. Reference coordinates for position restraints were scaled with the pressure coupling. Center of mass motion removal was off in order to prevent the induction of membrane curvature. Three independent systems were run.

The final frame of each 1 µs coarse-grained simulation (outward- and inward-facing) was backmapped using the backward.py script [2] to have an equilibrated membrane in all atom representation using the CHARMM36m force field [6]. The backmapped SbmA structure was then replaced with the SbmA structure to exclude infrequent protein distortions due to the double coordinate conversion. In addition, we adjusted some of the side chain torsion of the glutamate ladder to improve the hydrogen bonding pattern to increase the stability of the SmbA dimer starting structure. To investigate the effect of residue protonation, we created several systems, in which the protonation state of a specific glutamate residue (unprotonated, E193, E203, E269, E276, E378) was protonated on each protomer, each time using the equilibrate membranes in coarse-grained

representation, resulting in six system per protonation state in both the inward-facing-open and the outward-open state. For each system, we carried out six simulations (repeats). As replacing the backmapped structure with the hydrogen bonds optimized structure can introduce clashes between the protein and the lipids, the starting system was subject to 100 rounds of energy minimization using the steepest descent algorithm. Each energy minimization had 10 steps. The systems were then subject to a short, 1000-step equilibration at 1K, designed to remove any remaining clashes between the lipids and protein residues. After this, the system was equilibrated for 2 ns at 310 K. At first, position restraints were present on the heavy atoms of the protein with a force constant of 1000 kJ mol^{-1} nm^{-2}. After 0.5 ns, the force of the position restraints was decreased each time to 100, 10, and finally, 1 kJ mol^{-1} nm^{-2}.

The six systems per transporter conformation and protonation state were then simulated for 1 µs of production run using the CHARMM36m force field [6] and GROMACS version 2019.2 or 2024.5 [7]. No position restraints were present during the production run. Temperature coupling at 310 K was done with the V-rescale algorithm [4] and pressure coupling at 1 bar in a semi-isotropic manner used the Parrinello-Rahman algorithm [5]. Neighbor searching was performed every 50 steps with the Verlet cutoff scheme. The PME algorithm was used for electrostatic interactions with a cutoff of 1.2 nm. A single cutoff was used for the van der Waals interactions of 1.2 nm with a force-switch modifier beginning at 1.0 nm.

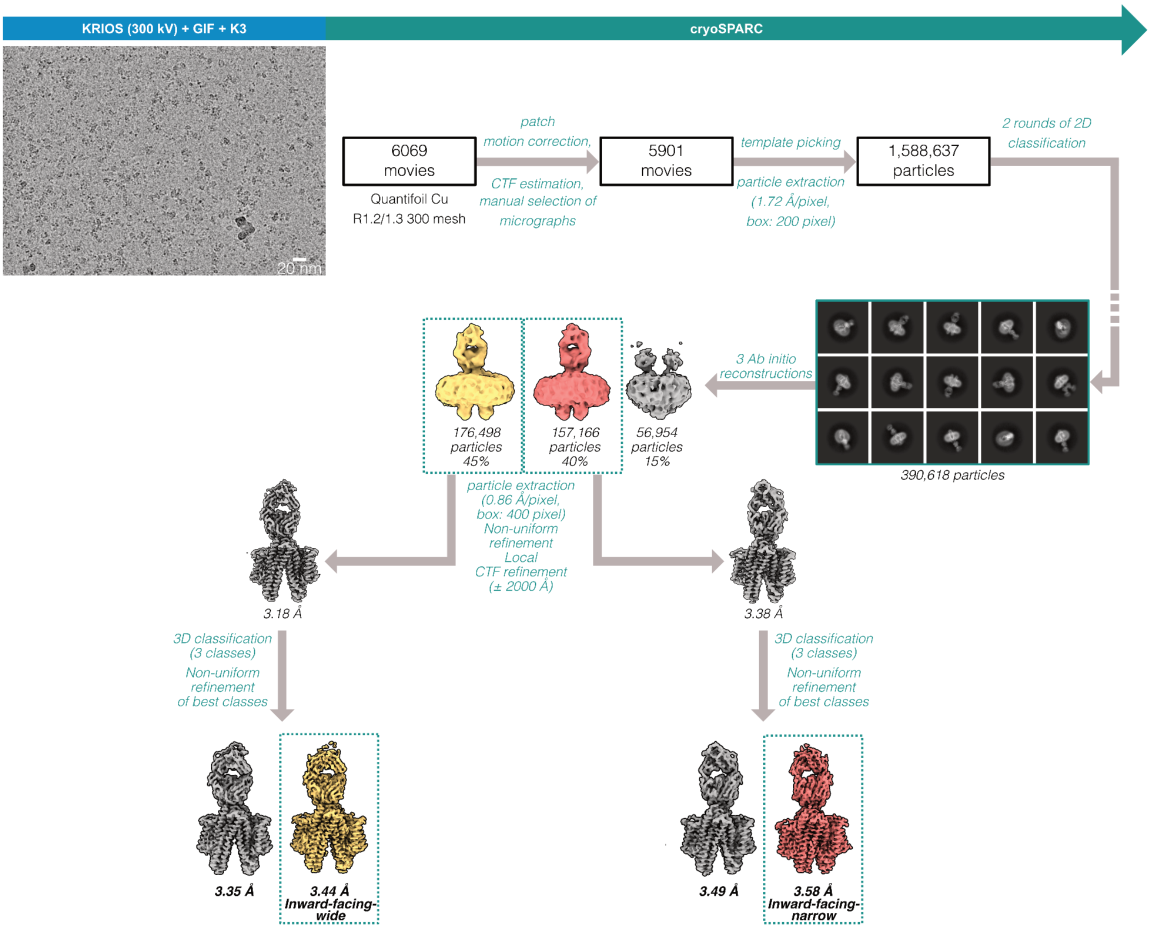

Supplementary Fig. 1. Cryo-EM reconstructions of SbmA in the inward-facing-narrow and -wide conformations bound to FabS11-1. Detailed image processing workflow including a representative micrograph showing the particle distribution on a Quantifoil Cu R1.2/1.3 300 mesh grid.

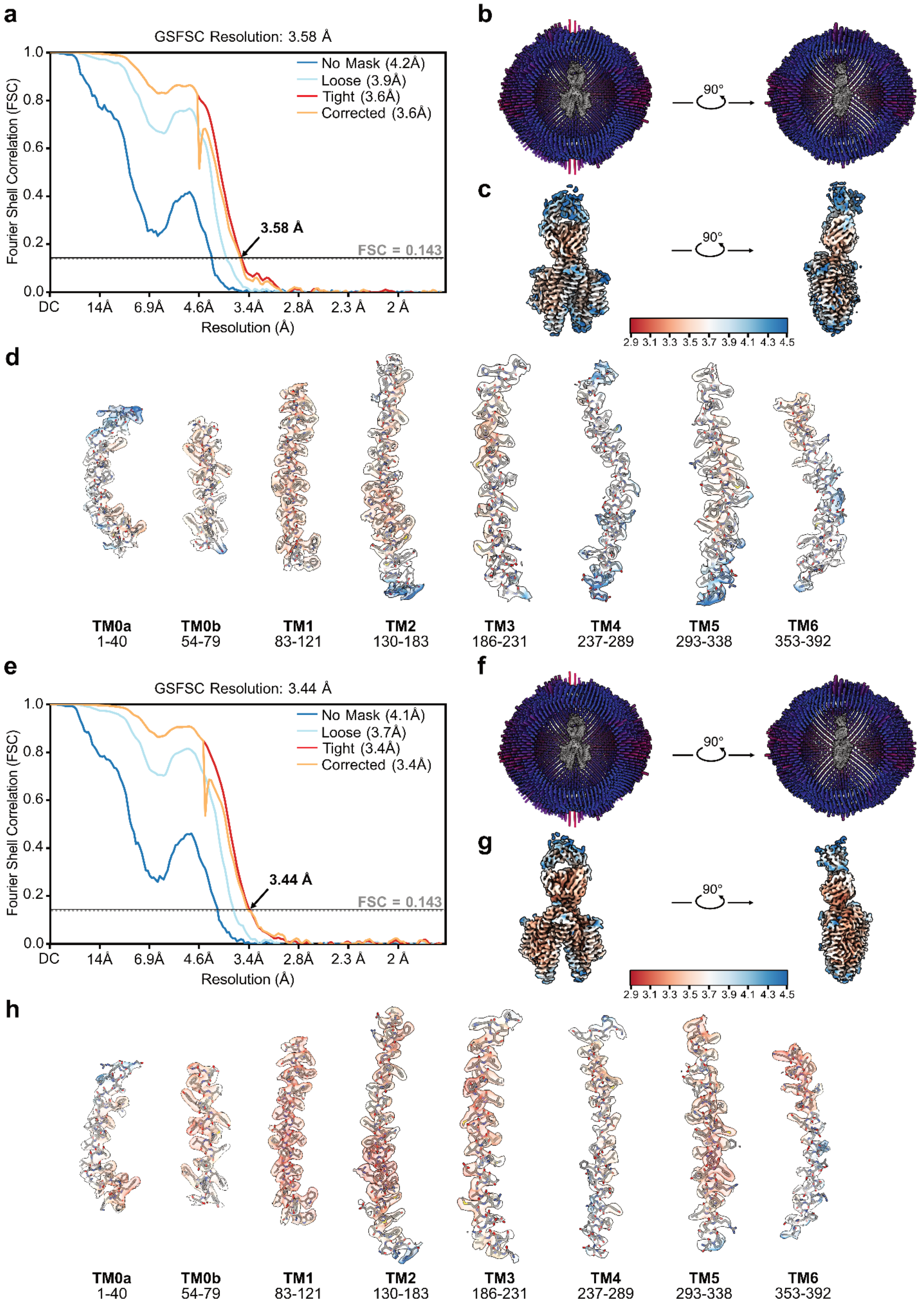
a
GSFSC Resolution: 3.58 Å
Fourier Shell Correlation (FSC)
No Mask (4.2Å)
Loose (3.9Å)
Tight (3.6Å)
Corrected (3.6Å)
3.58 Å
FSC = 0.143
DC 14Å 6.9Å 4.6Å 3.4Å 2.8Å 2.3 Å 2 Å
Resolution (Å)
b
90°
c
90°
2.9 3.1 3.3 3.5 3.7 3.9 4.1 4.3 4.5
d
TM0a 1-40
TM0b 54-79
TM1 83-121
TM2 130-183
TM3 186-231
TM4 237-289
TM5 293-338
TM6 353-392
e
GSFSC Resolution: 3.44 Å
Fourier Shell Correlation (FSC)
No Mask (4.1Å)
Loose (3.7Å)
Tight (3.4Å)
Corrected (3.4Å)
3.44 Å
FSC = 0.143
DC 14Å 6.9Å 4.6Å 3.4Å 2.8Å 2.3 Å 2 Å
Resolution (Å)
f
90°
g
90°
2.9 3.1 3.3 3.5 3.7 3.9 4.1 4.3 4.5
h
TM0a 1-40
TM0b 54-79
TM1 83-121
TM2 130-183
TM3 186-231
TM4 237-289
TM5 293-338
TM6 353-392

Supplementary Fig. 2. Validation of SbmA in the inward-facing-narrow and -wide conformations bound to FabS11-1. (a, e) FSC plot of the final reconstruction with the arrow indicating the global resolution based on the threshold of FSC = 0.143 for SbmA in the (a) inward-facing-narrow and (e) -wide conformations. (b, f) Angular distribution plot of particles included in the asymmetric 3D reconstruction. The number of particles with their respective orientation is represented by the length and color of the cylinders. (c, g) Final reconstructed map colored according to the local resolution estimated in cryoSPARC. (d, h) Densities (semi-transparent surface renderings) for indicated residue ranges are shown in relation to the fitted atomic model (shown in stick representation). The surface renderings of the selected regions are colored according to the estimated local resolution.

a

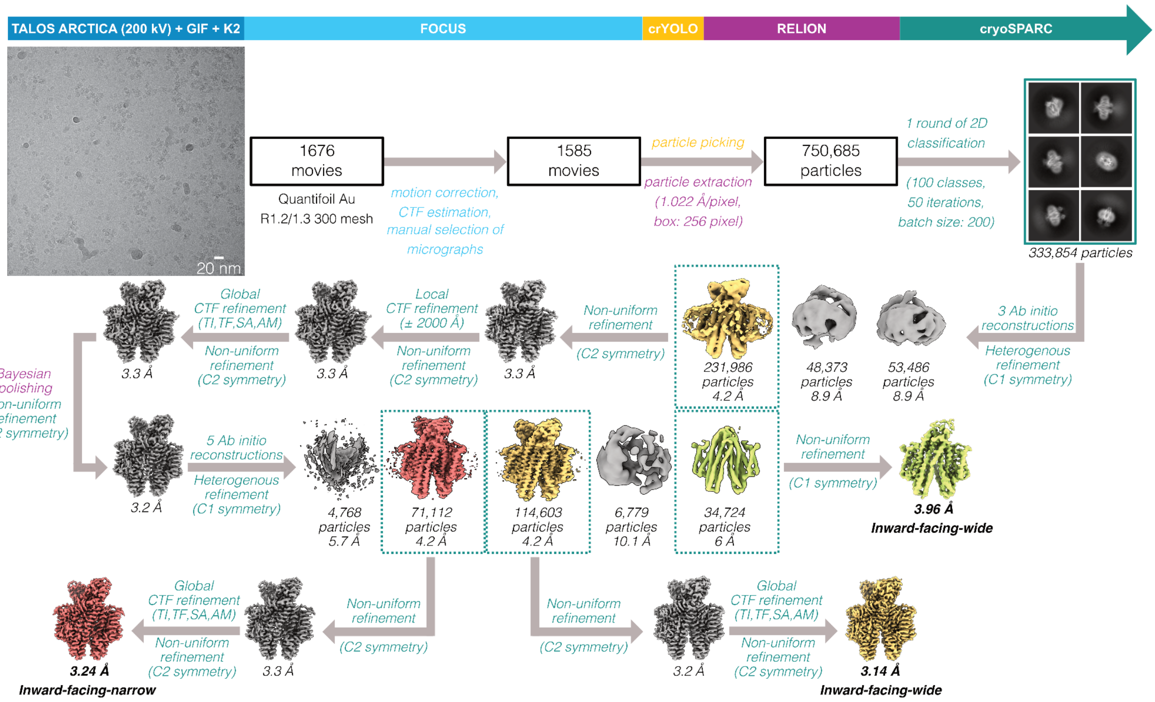

Supplementary Fig. 3. Cryo-EM reconstructions of SbmA in the inward-facing-narrow and -wide conformations bound to Sy2. Detailed image processing workflow including a representative micrograph at a defocus of volume of -1.4 μm showing the particle distribution on a Quantifoil Au R1.2/1.3 300 mesh grid. Abbreviations correspond to the following: TI = Tilt, TF = Trefoil, SA = Spherical Aberration, AM = Anisotropic magnification.

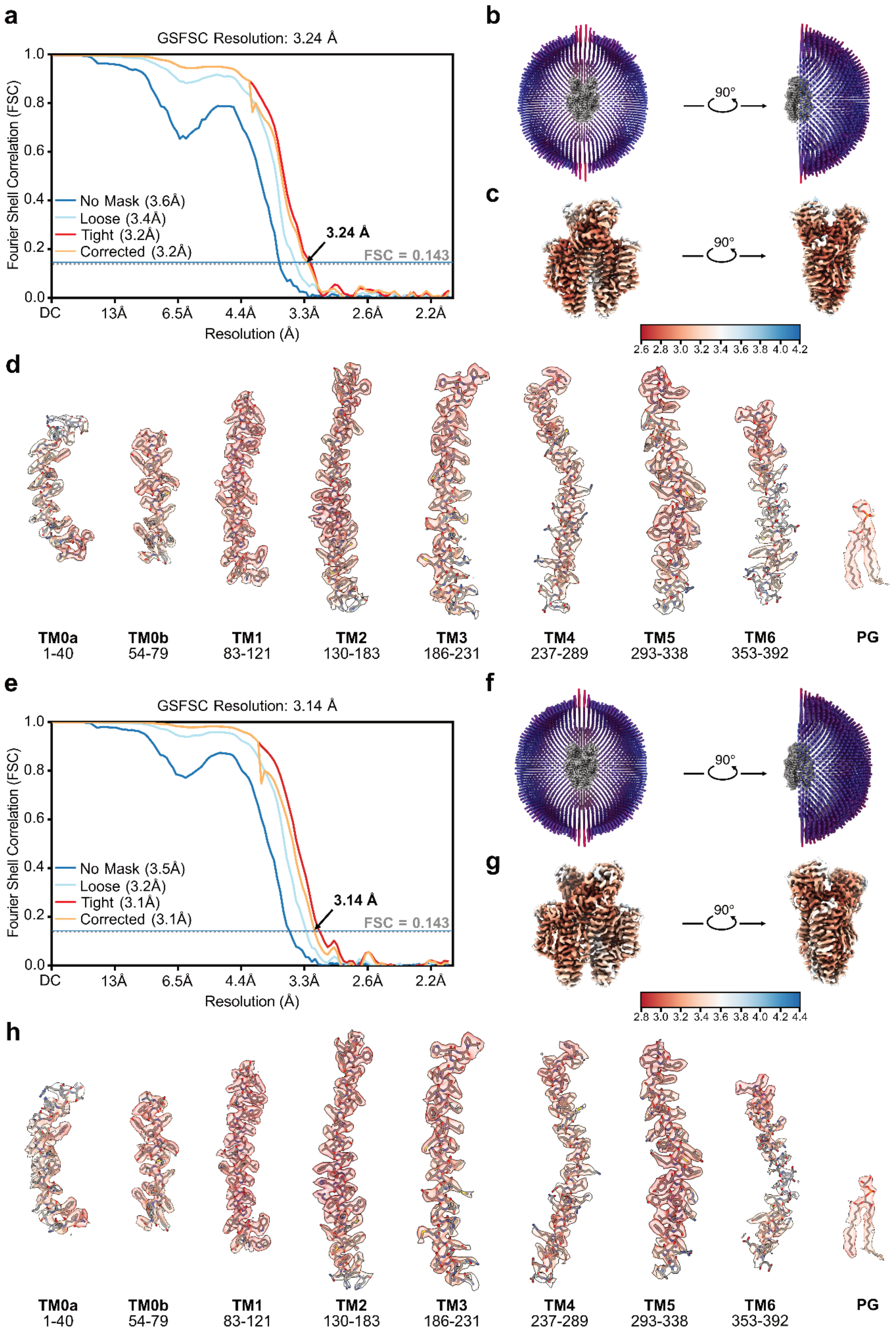
a
GSFSC Resolution: 3.24 Å
Fourier Shell Correlation (FSC)
No Mask (3.6Å)
Loose (3.4Å)
Tight (3.2Å)
Corrected (3.2Å)
3.24 Å
FSC = 0.143
DC
13Å
6.5Å
4.4Å
3.3Å
2.6Å
2.2Å
Resolution (Å)
b
90°
c
90°
2.6 2.8 3.0 3.2 3.4 3.6 3.8 4.0 4.2
d
TM0a 1-40
TM0b 54-79
TM1 83-121
TM2 130-183
TM3 186-231
TM4 237-289
TM5 293-338
TM6 353-392
PG
e
GSFSC Resolution: 3.14 Å
Fourier Shell Correlation (FSC)
No Mask (3.5Å)
Loose (3.2Å)
Tight (3.1Å)
Corrected (3.1Å)
3.14 Å
FSC = 0.143
DC
13Å
6.5Å
4.4Å
3.3Å
2.6Å
2.2Å
Resolution (Å)
f
90°
g
90°
2.8 3.0 3.2 3.4 3.6 3.8 4.0 4.2 4.4
h
TM0a 1-40
TM0b 54-79
TM1 83-121
TM2 130-183
TM3 186-231
TM4 237-289
TM5 293-338
TM6 353-392
PG

Supplementary Fig. 4. Validation of SbmA in the inward-facing-narrow and -wide conformations bound to two Sy2. (a, e) FSC plot of the final reconstruction with the arrow indicating the global resolution based on the threshold of FSC = 0.143 for SbmA in the (a) inward-facing-narrow and (e) -wide conformations. (b, f) Angular distribution plot of particles included in the symmetric 3D reconstruction. The number of particles with their respective orientation is represented by the length and color of the cylinders. (c, g) Final reconstructed map colored according to the local resolution estimated in cryoSPARC. (d, h) Densities (semi-transparent surface renderings) for indicated residue ranges are shown in relation to the fitted atomic model (shown in stick representation). The surface renderings of the selected regions are colored according to the estimated local resolution.

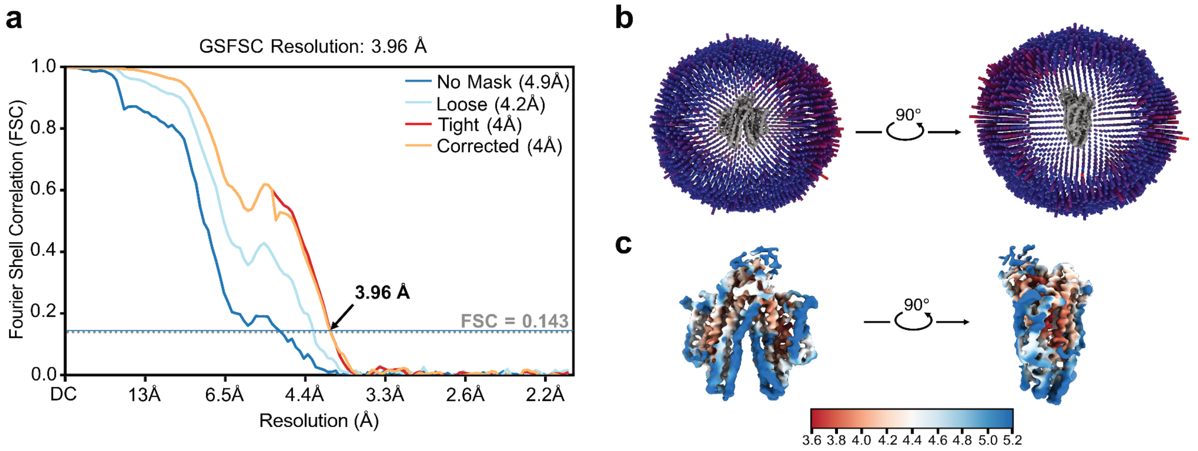

Supplementary Fig. 5. Validation of SbmA in the inward-facing-narrow and -wide conformations bound to a single Sy2. (a) FSC plot of the final reconstruction with the arrow indicating the global resolution based on the threshold of FSC = 0.143. (b) Angular distribution plot of particles included in the asymmetric 3D reconstruction. The number of particles with their respective orientation is represented by the length and color of the cylinders. (c) Final reconstructed map colored according to the local resolution estimated in cryoSPARC.

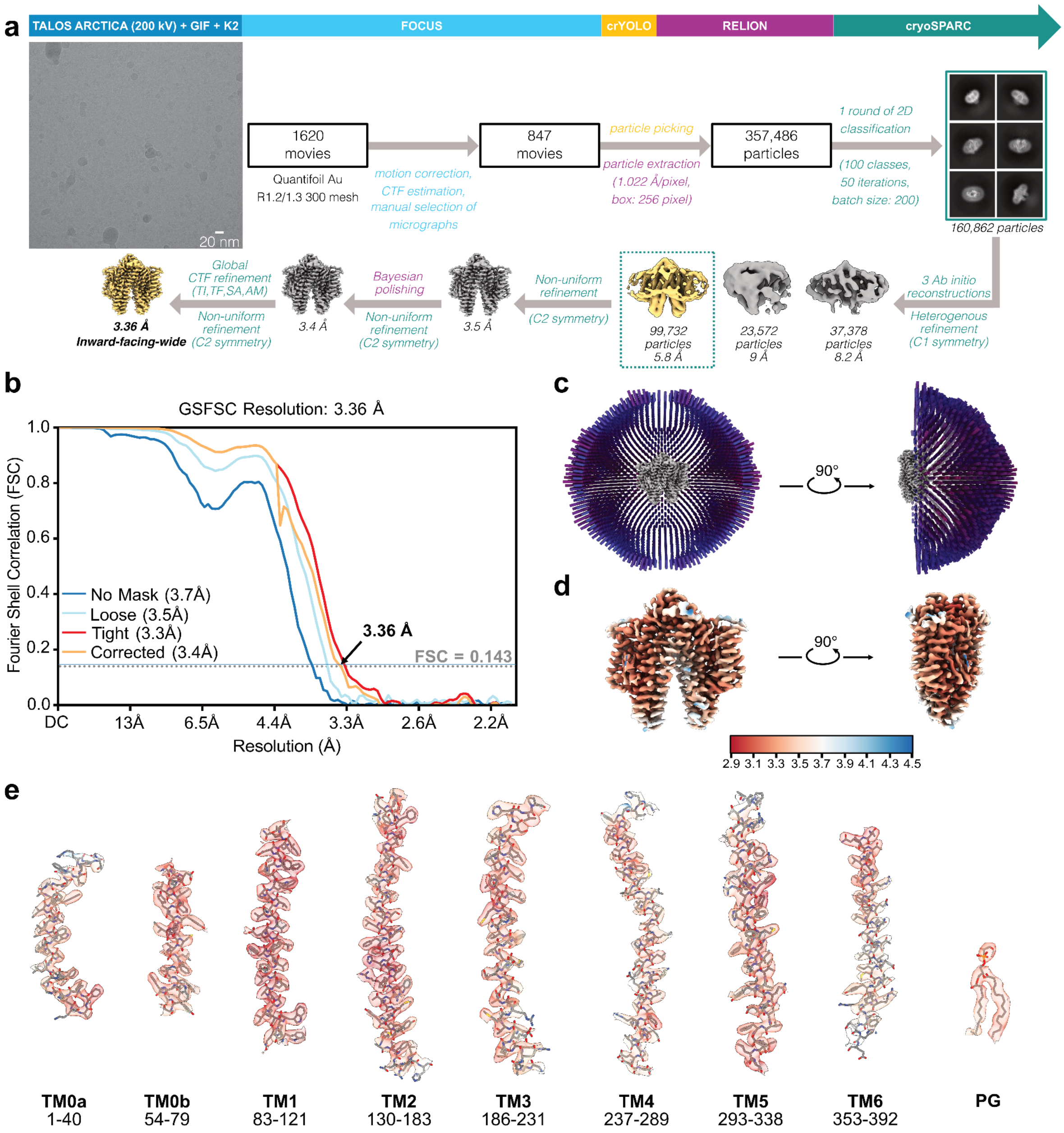

Supplementary Fig. 6. Cryo-EM reconstruction and validation of SbmA in the inward-facing-wide conformation in detergent solution at high pH. (a) Detailed image processing workflow including a representative micrograph at a defocus of volume of -1.4 μm showing the particle distribution on a Quantifoil Au R1.2/1.3 300 mesh grid. Abbreviations correspond to the following: TI = Tilt, TF = Trefoil, SA = Spherical Aberration, AM = Anisotropic magnification. (b) FSC plot of the final reconstruction with the arrow indicating the global resolution based on the threshold of FSC = 0.143. (c) Angular distribution plot of particles included in the symmetric 3D reconstruction. The number of particles with their respective orientation is represented by the length and

color of the cylinders. (d) Final reconstructed map colored according to the local resolution estimated in cryoSPARC. (e) Densities (semi-transparent surface renderings) for indicated residue ranges are shown in relation to the fitted atomic model (shown in stick representation). The surface renderings of the selected regions are colored according to the estimated local resolution.

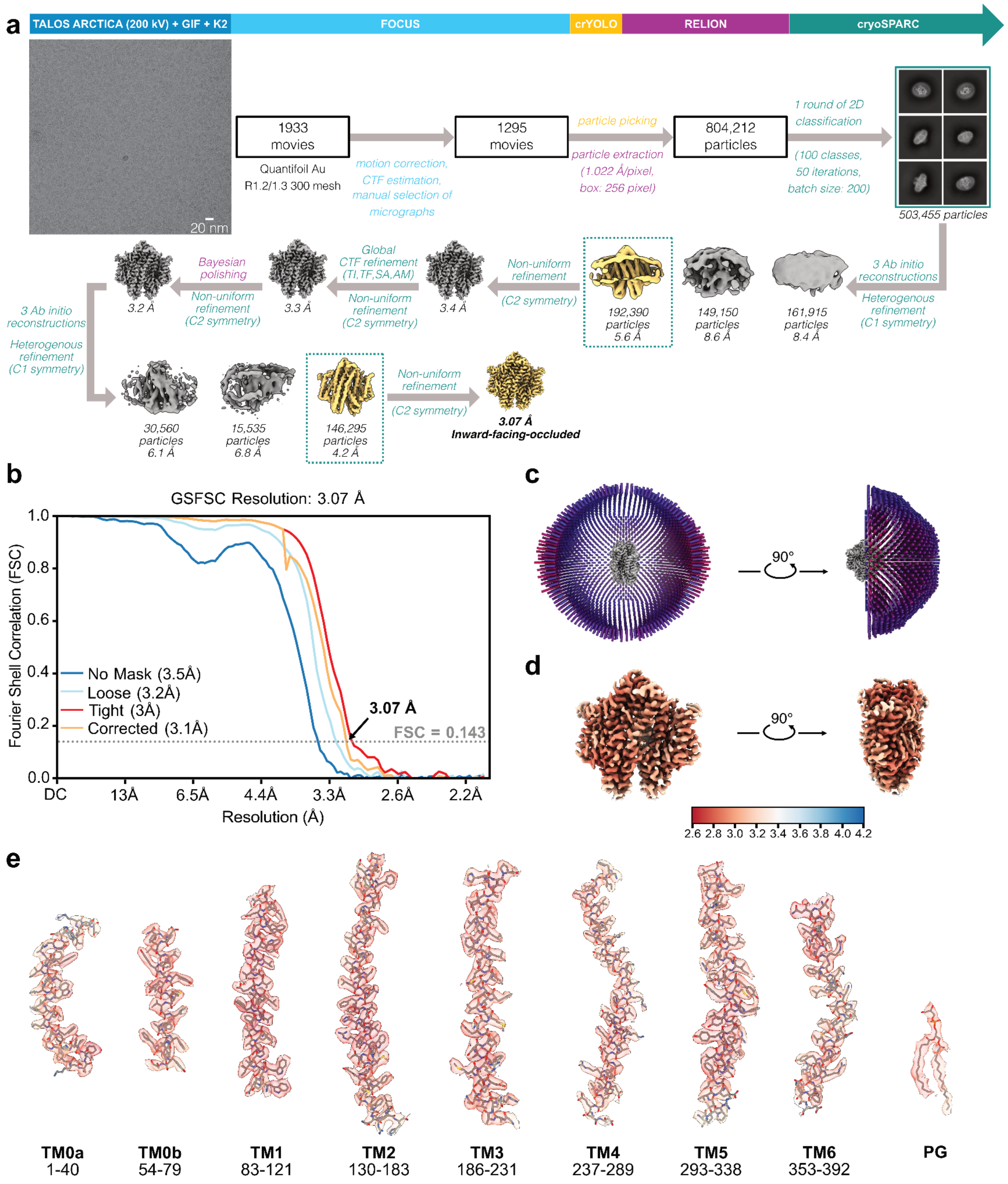

Supplementary Fig. 7. Cryo-EM reconstruction and validation of SbmA in the inward-facing-occluded conformation in lipid nanodiscs at low pH. (a) Detailed image processing workflow including a representative micrograph at a defocus of volume of -1.4 µm showing the particle distribution on a Quantifoil Au R1.2/1.3 300 mesh grid. (b) FSC plot of the final reconstruction with the arrow indicating the global resolution based on the threshold of FSC = 0.143. (c) Angular distribution plot of particles in-

cluded in the symmetric 3D reconstruction. The number of particles with their respective orientation is represented by the length and color of the cylinders. (d) Final reconstructed map colored according to the local resolution estimated in cryoSPARC. (e) Densities (semi-transparent surface renderings) for indicated residue ranges are shown in relation to the fitted atomic model (shown in stick representation). The surface renderings of the selected regions are colored according to the estimated local resolution.

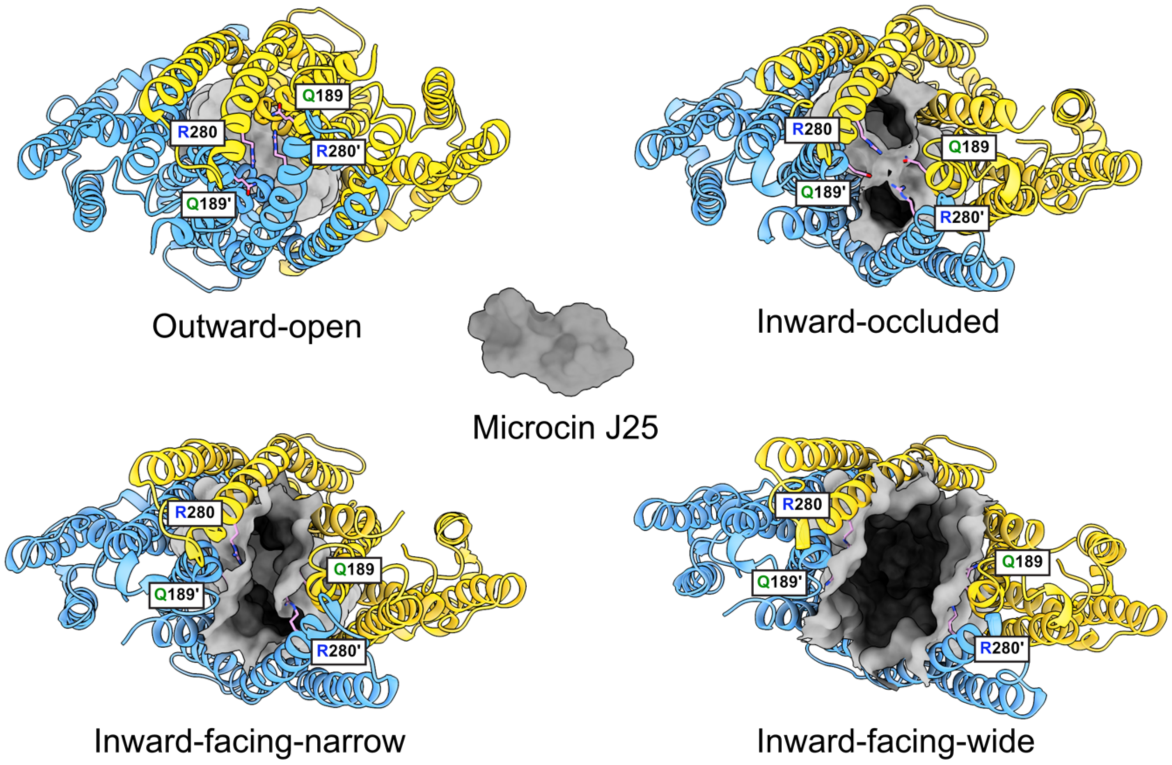

Supplementary Fig. 8. Comparison of the opening of the internal cavity viewed from the cytoplasm between the outward-open, inward-occluded, inward-facing-narrow and inward-facing-wide (Table 1, identifiers A, B, C and G). Models are shown in cartoon representation and colored by chain, internal cavity is shown as gray surface. To compare the size of the opening to the size of a substrate of SbmA, a surface model of Microcin J25 (PDB 1pp5) has been included. The side chains of Q189, Q189', R385 and R385' are colored by atom with carbons highlighted in pink to show the initial point of contact for closure of the second internal cytoplasmic gate, which can be observed in the outward-occluded conformation.

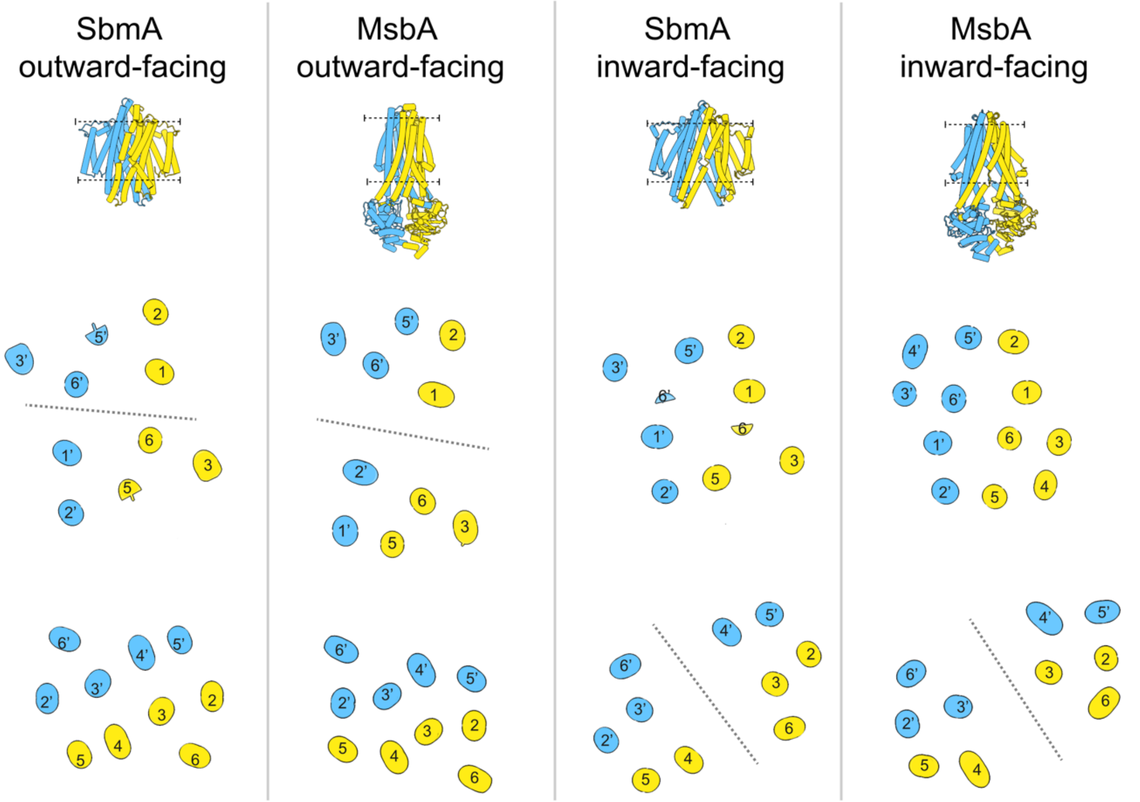

Supplementary Fig. 9. TM movement between the outward-facing and inward-facing conformations of SbmA compared to the full-length ABC transporter MsbA (outward-facing SbmA – Table 1 identifier A, outward-facing MsbA - PDB 8tso, inward-facing SbmA - Table 1 identifier G, inward-facing MsbA – PDB 7mew). The top tube models, colored by chain, provide a side view from the membrane plane and the black dotted lines indicate the position of the slice-through images shown below. The gray dotted lines are included to illustrate the separation into two distinct domains that create the opening of the cavity.

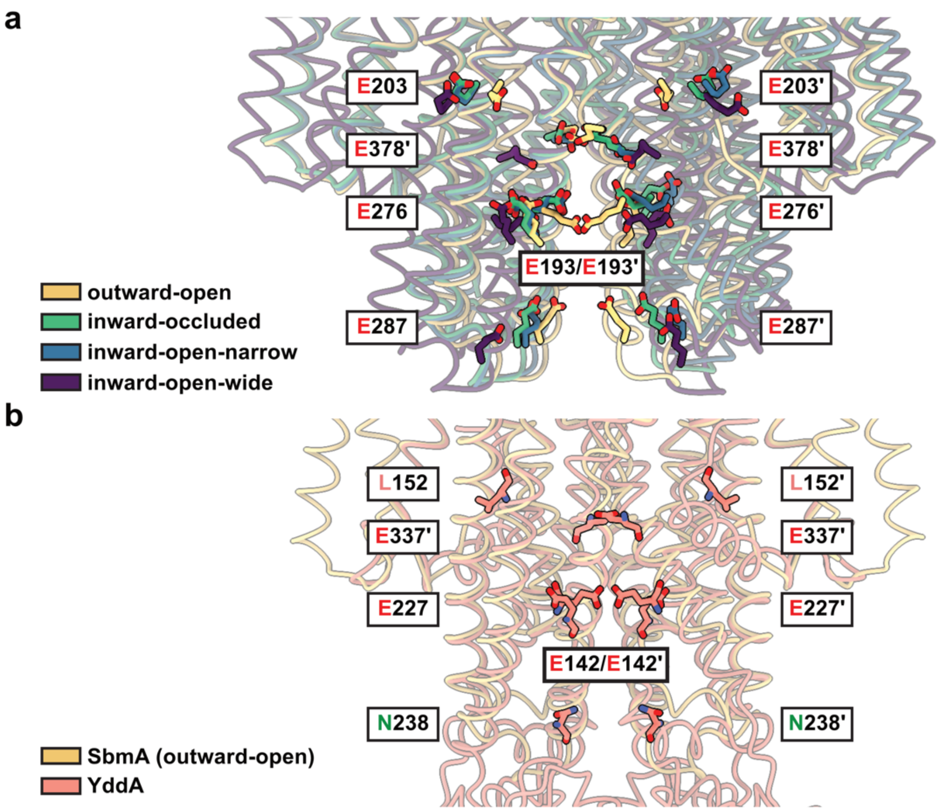

Supplementary Fig. 10. Movement and conservation of the glutamate ladder. (a) Movement of the glutamate ladder from the outward-open to the inward-open conformation. An overlay of SbmA in the different conformations (outward-open in gold, inward-occluded in green, inward-open-narrow in blue, inward-open-wide in purple) is shown in cartoon representation with the residues forming the glutamate ladder shown as sticks. (b) Presence of the SbmA glutamate ladder in the *E. coli* type IV ABC transporter YddA. An overlay of SbmA in the outward-open (gold) and YddA (salmon) is shown with the equivalent residues of the SbmA glutamate ladder in YddA shown as sticks.

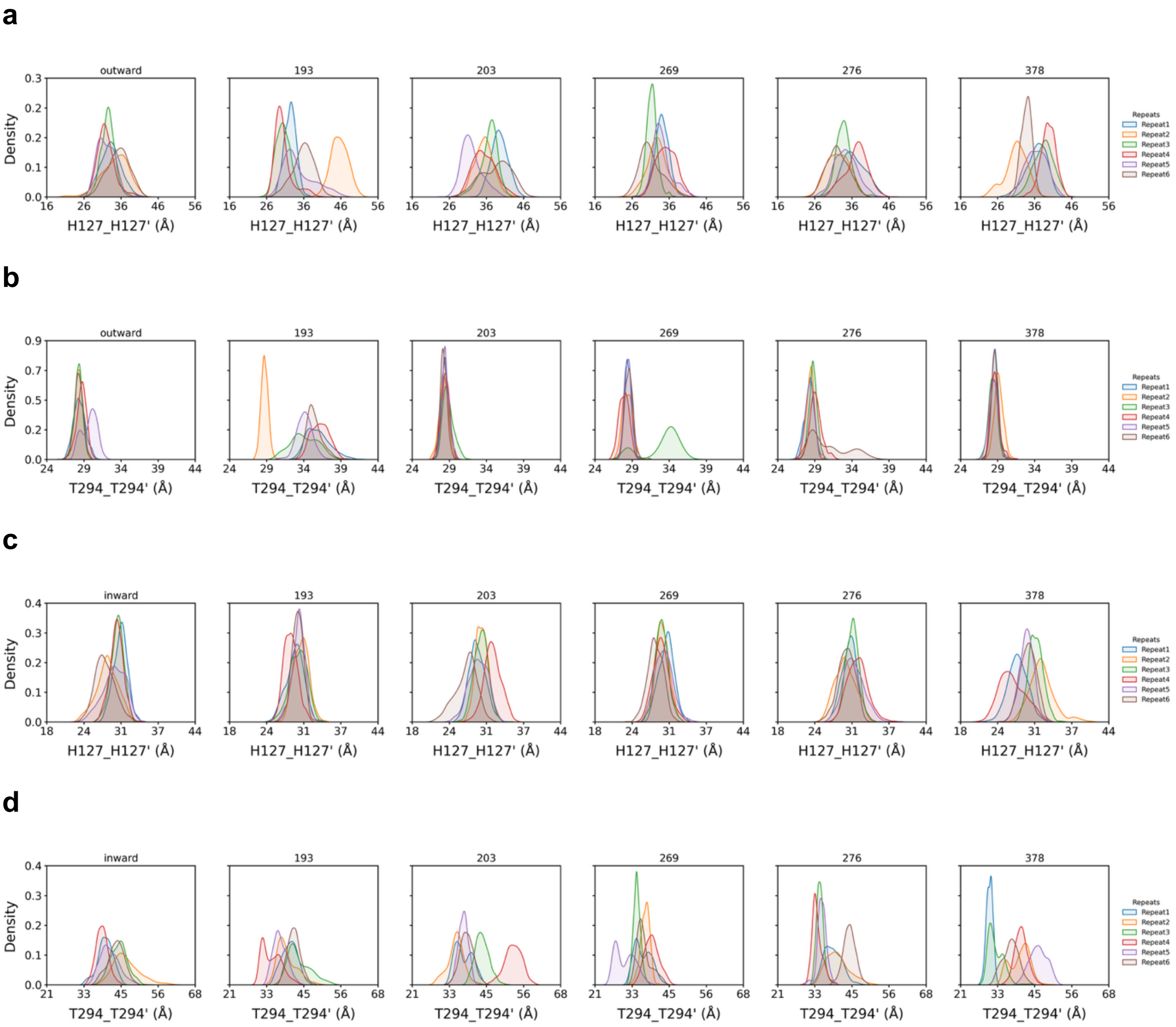

Supplementary Fig. 11. Distance distributions of the periplasmic residue (H127-H127’) and the cytoplasmic residue pairs (T294-T294’), also used in our DEER analysis, in different protonation states. MD simulations of outward-facing and inward-facing SbmA were carried out using standard protonation of the glutamates of the glutamate latter (left column or individual protonation of a glutamate of the glutamate ladder (E193, E203, E269, E276, E378). Six parallel simulations were carried out for each setup. (a, c) Distance distribution of (a) outward-facing and (c) inward-facing SbmA measured at the periplasmic side of SbmA of the Cα atoms of the residue pair (H127-H127’) in different protonation states. (b, d) Distance distribution of (b) outward-facing and d) inward-facing SbmA measured at the cytosolic side of SbmA of the Cα atoms of the residue pair (T294-T294’) for different protonation states.

Consistent with cryo-EM structures and EPR data, simulations of the outward-open SbmA (a) show broad peaks at the periplasmic side (H127-H127' distance) with most

interatomic distances ranging from 30 to 40 Å, and a sharp peak around 30 Å on the cytoplasmic side. This is expected for the outward-open conformation in which the outer gate is more mobile because of the separated domains, while the inner gate (b) is closed and stabilized by domain-domain interactions. Conversely, simulations of the inward-facing-wide structure revealed a sharper peak for the periplasmic gate (c), centered around 30 Å, than at the cytoplasmic gate (d) where the distance distribution is extremely broad. Protonation of E203, E276, and E378 (a) resulted in a spread of distances from below 30 Å to above 40 Å on the periplasmic side, while (b) at the intracellular gate a single sharp peak at 30 Å remained, suggesting that protonation of these glutamates might contribute to stabilizing the outward-open state of SbmA. The peak distribution observed for the simulations of protonated E269 (and possibly also E276) resulted in a similar distribution to distances (a, b), except for one simulation that showed an initial opening of the inner gate as evident by the smaller second peak of the inner gate distance, suggesting a reduced stability of the outward-open state. Protonation of E193 (a) resulted in two sharper peaks at low distance at the periplasmic side, and (b) a transition towards a bigger separation at the cytoplasmic gate. These differences are indicative of the beginning of a transition from an outward-open towards an inward-open state, as the outer gate moves toward closure, while the inner gate weakens and shows the first step towards opening. This data suggests that a protonation of E203 and E378 could stabilize an outward-open state, while protonation of E269, E276 or of E193 could trigger closure of the periplasmic gate.

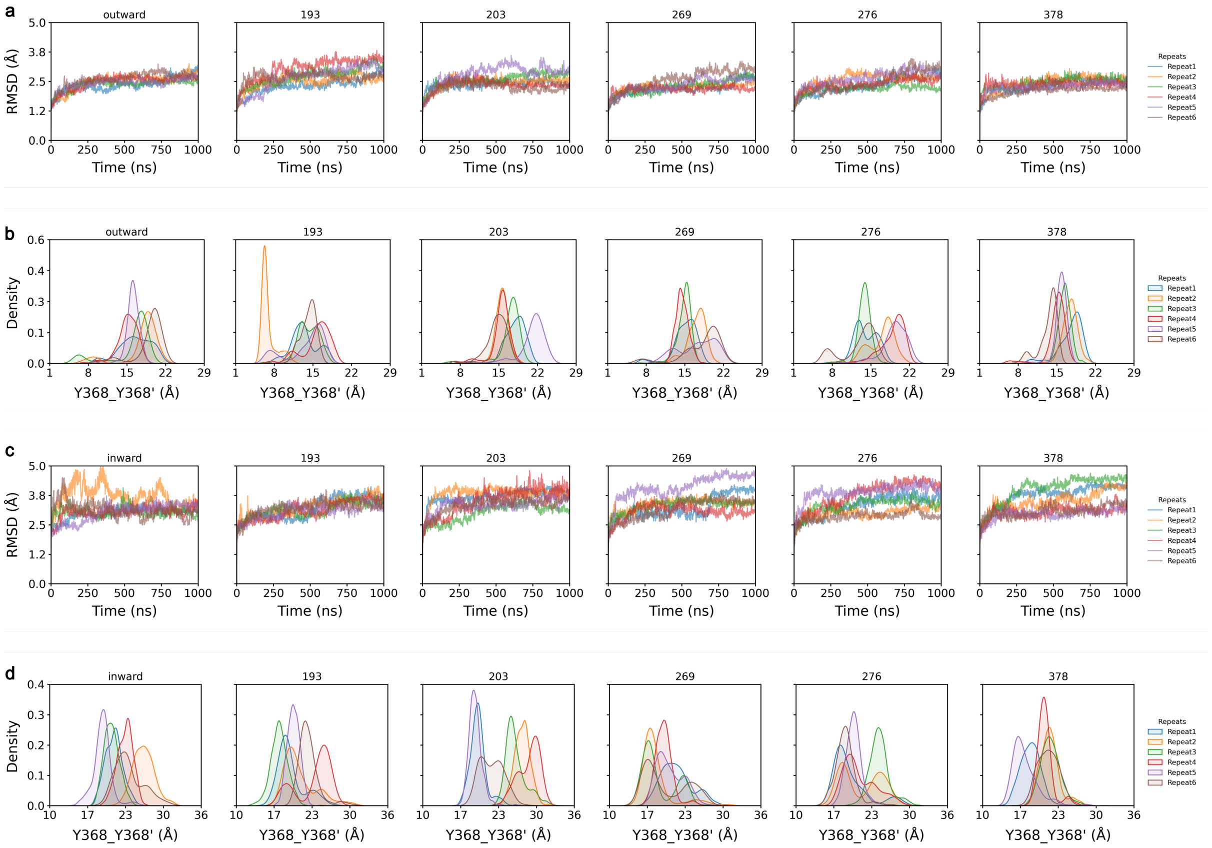

Supplementary Fig. 12. Root mean square deviation (RMSD), central gate distance distribution. (a) Root mean square deviation (RMSD) of the Cα atoms of out-ward-open SbmA fitted to Cα atoms of each repeat (six repeats per system) of un-protonated SbmA and of the five individually protonated residuesion states (193, 203, 269, 276, 378). (b) Distance distribution of the central gate of SbmA of the Cα atoms of the residue pair (Y368-Y368') for different protonation states. (c) RMSD of the inward-facing-open state of all six system. (d) Distance distribution of the central gate of SbmA of the Cα atoms of the residue pair (Y368-Y368') for all protonation states.

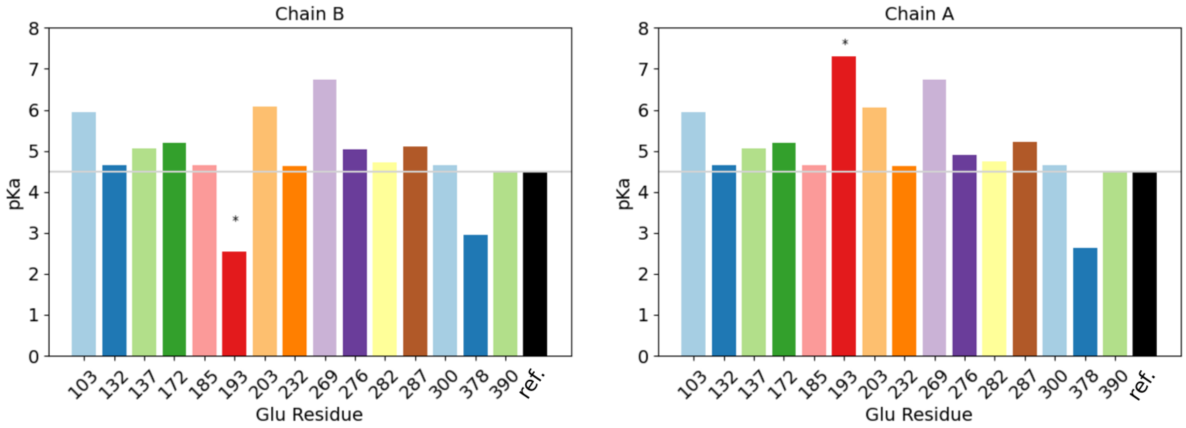

Supplementary Fig. 13. Protonation of residues of the glutamate ladder. pKa glutamate prediction. pKa values of the glutamate residues of each individual chain of SbmA (Chain A and Chain B) predicted using PROPKA version 3.4.0 [8]. The asterisk indicates that the glutamate residue at position 193 is coupled i.e. the protonation of one of them will affect the protonation state of the other.

To further investigate the stabilization/destabilization of SmbA by glutamate protonation, we carried out simulation using the classical all atom force field CHARMM36m [6], whereby each glutamate was simulated in the protonated and deprotonated form to assess the response of SbmA to individual glutamate protonations (see Supplementary Fig. 11 and 12).

The limitation of classical force field based MD simulations, i.e. that the protonation state is fixed at start, does not fully describe the chemistry of protonatable residues, as protons can dynamically bind and unbind. This limitation can be overcome by the recent developments in constant pH MD simulations [9, 10, 11] for special cases such as water-exposed protonatable groups that are not linked to a typically slow protonation-conformation coupling mechanism. The residues of the deeply buried glutamate ladder are central to protonation-conformation coupling of SbmA, as their protonation (especially E193) leads to the conformational transition from the outward-open to the inward-open state, precluding the use these advanced methods. Source data are provided as a Source Data file.

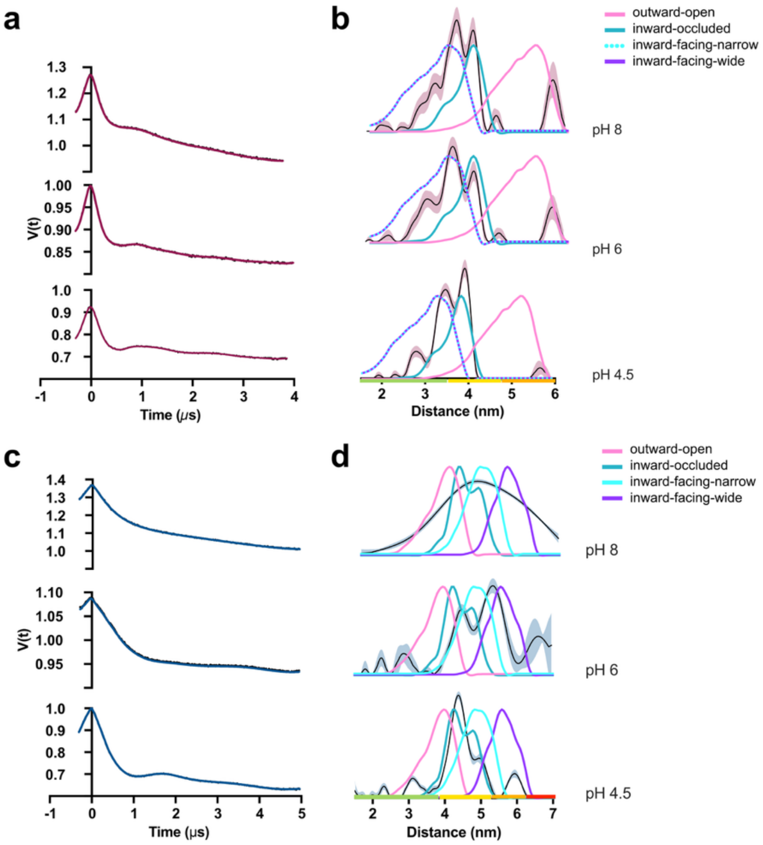

Supplementary Fig. 14. Monitoring the entire conformational ensemble of SbmA in solution by EPR spectroscopy – DeerLab analysis [12]. (a) PELDOR raw time-domain traces for H127R1 site fit with multiple dipolar pathways using DeerLab software package [13, 14] to account for the "2+1" effect contribution (red) [15]. (b) Overlay for H127R1 of predicted (using MMM) distance distributions from cryo-EM structures of the outward-open (pink), inward-occluded (blue), inward-facing-narrow (cyan) and inward-facing-wide (purple) states, and experimental distance distributions from DeerLab (black). Salmon-shaded regions correspond to the 95% confidence intervals of the mean distance, and the "traffic light" system assessing reliability taken from the DeerAnalysis analysis in Figure 4. (c) PELDOR raw time-domain traces for the T294R1 mutant fit with multiple dipolar pathways using DeerLab software package [13, 14] to account for the "2+1" effect contribution (blue), d, Overlay for T294R1 of predicted (using MMM) distance distributions from CryoEM structures of the outward-open (pink), inward-occluded (blue), inward-facing-narrow (cyan) and inward-facing-wide (purple) states, and experimental distance distributions from DeerLab (black). Grey-shaded regions correspond to the 95% confidence intervals of the mean distance, and the

“traffic light” system assessing reliability (taken from DeerAnalysis analysis in Figure 4). Source data are provided as a Source Data file.

Supplementary Table 1. Summary of the molecular dynamic simulations. Each sus-tem was simulated six time (n=6), and each trajectory was 1 µs long.

SbmA confor-mation	unproto-nated	E193+E193’	E203+E203’	E269+E269’	E276+E276’	E378+E378’
inward-facing	n=6, (1 µs)	n=6, (1 µs)	n=6, (1 µs)	n=6, (1 µs)	n=6, (1 µs)	n=6, (1 µs)
outward-facing	n=6, (1 µs)	n=6, (1 µs)	n=6, (1 µs)	n=6, (1 µs)	n=6, (1 µs)	n=6, (1 µs)

Supplementary Table 2 – Data collection, processing and refinement statistics of the 300 kV cryo-EM structures

	SbmA-Fab (1x) inward-facing-wide in lipid nanodiscs (EMD-51036, PDB 9g4e, EMPIAR 12192)	SbmA-Fab (1x) inward-facing-narrow in lipid nanodiscs (EMD-51037, PDB 9g4f, EMPIAR 12192)
**Data collection and processing**		
Microscope	Thermo Fisher Krios G3i	
Energy filter	Gatan GIF BioQuantum K3	
Detector	Gatan K3	
Camera mode	counting mode	
Voltage (kV)	300	
Nominal magnification	105,000x	
Pixel size (Å)	0.86	
Exposure navigation	Beam-image shift (AFIS)	
Number of frames	40	
Total electron exposure (e^-/Å^2)	42.39	
Defocus range (μm)	-0.9 to -2.7	
Data collection software	EPU 2.10.0.5REL	
Micrographs collected	6069	6069
Micrographs used	5901	5901
Total number of extracted particles	1,294,264	1,294,264
Final number of particles	56,582	50,775
Symmetry imposed	C1	C1
Global map resolution (Å)	3.44	3.58
Map resolution range (Å)	~3.0 – ~5.0	~3.2 – ~5.5
FSC threshold	0.143	0.143
**Refinement**		
Initial model used	*Ab-initio*	*Ab-initio*
Model resolution	3.66	3.79
FSC threshold	0.5	0.5
Map sharpening B-factor (Å^2)	-	-
Model composition		
Chains	4	4
Non-hydrogen atoms	9660	9660
Protein residues	1215	1215
Water	-	-
Ligands	-	-
B factors (Å^2)		
Protein (min/max/mean)	2.71/103.36/41.58	20.15/159.51/72.29
Ligand (min/max/mean)	-	-
R.m.s. deviation		
Bond lengths (Å)	0.007	0.012
Bond angles (°)	0.761	0.945
Validation		
Molprobity score	1.72	1.93
Clashscore	5.77	7.75
Ramachandran plot		
Favoured (%)	94.03	91.30
Allowed (%)	5.97	8.70
Outliers (%)	0	0
Rotamer outliers (%)	0	0
Rama-Z		
Whole	-0.27 (0.24) (N = 1207)	-1.02 (0.24) (N = 1207)
Helix	0.89 (0.20) (N = 658)	0.23 (0.20) (N = 635)
Sheet	-0.12 (0.36) (N = 221)	-0.36 (0.39) (N = 187)
Loop	-2.39 (0.30) (N = 328)	-2.31 (0.30) (N = 385)

Supplementary Table 3 – Data collection, processing and refinement statistics of the 200 kV cryo-EM structures

	**SbmA-Sb2 (2x) inward-facing-narrow** in detergent micelles **(EMD-50994, PDB 9g3d, EMPIAR 12888)**	**SbmA-Sb2 (2x) inward-facing-wide** in detergent micelles **(EMD-50996, PDB 9g3e, EMPIAR 12888)**	**SbmA-Sb2 (1x) inward-facing-wide** in detergent micelles **(EMD-50995, EMPIAR 12888)**	**SbmA inward-facing-wide** in detergent micelles **(EMD-50997, PDB 9g3f, EMPIAR 12889)**	**SbmA inward-facing-occluded** in lipid nanodiscs **(EMD-50998, PDB 9g3g, EMPIAR 12890)**
**Data collection and processing**					
Microscope			Thermo Fisher Talos Arctica		
Energy filter			Gatan GIF BioQuantum K2		
Detector			Gatan K2 Summit		
Camera mode			counting mode		
Voltage (kV)			200		
Nominal magnification			130,000x		
Pixel size (Å)			1.022		
Exposure navigation			Beam-image shift with a 3 x 3 pattern (Serial EM)		
Number of frames			60		
Total electron exposure ($e^-/Å^2$)			50.1		
Defocus range (µm)			-0.5 to -2.0		
Data collection software			Serial EM v3.9.0 beta		
Micrographs collected	1676	1676	1676	1620	1933
Micrographs used	1585	1585	1585	874	1295
Total number of extracted particles	750,685	750,685	750,685	357,486	804,212
Final number of particles	71,122	114,603	34,724	99,732	146,295
Symmetry imposed	C2	C2	C1	C2	C2
Global map resolution (Å)*	3.24	3.14	3.96	3.36	3.07
Map resolution range (Å)	~2.6 – ~4.2	~2.8 – ~4.4	~3.6 – ~5.2	~2.9 to ~3.5	~2.6 to ~4.2
FSC threshold	0.143	0.143	0.143	0.143	0.143
**Refinement**					
Initial model used	*Ab-initio* (Model Angelo)	*Ab-initio* (Model Angelo)	-	*Ab-initio* (Model Angelo)	*Ab-initio* (Model Angelo)
Model resolution	3.4	3.4	-	3.5	3.1
FSC threshold	0.5	0.5	-	0.5	0.5
Map sharpening B-factor ($Å^2$)	-104.6	-107.4	-	-117.4	-117.4
Model composition			-		
Chains	4	4	-	2	2
Non-hydrogen atoms	8210	8255	-	6419	6482
Protein residues	1010	1015	-	778	784
Water	-	-	-	-	-
Ligands					
PGT	2	2	-	2	2
B factors ($Å^2$)					
Protein (min/max/mean)	2.79/132.31/54.04	0.00/120.12/40.93	-	1.32/117.55/50.97	0.00/73.49/25.74
Ligand (min/max/mean)	44.48/72.00/52.13	23.76/76.60/39.52	-	42.97/79.05/53.18	8.63/37.59/24.13
R.m.s. deviation					
Bond lengths (Å)	0.003	0.003	-	0.003	0.002
Bond angles (°)	0.490	0.529	-	0.541	0.405
Validation					
Molprobity score	1.78	1.55	-	1.46	1.19
Clashscore	7.57	5.59	-	5.12	3.92
Ramachandran plot					
Favoured (%)	94.61	96.33	-	96.90	97.95
Allowed (%)	5.39	3.67	-	3.10	2.05
Outliers (%)	0	0	-	0	0
Rotamer outliers (%)	0	0	-	0	0
Rama-Z					
Whole	0.87 (0.27) (N = 1002)	1.19 (0.26) (N = 1007)	-	-1.83 (0.28) (N= 774)	1.58 (0.28) (N= 780)
Helix	1.50 (0.20 (N = 653)	1.80 (0.19) (N = 666)	-	-0.92 (0.19) (N = 626)	1.49 (0.19) (N = 651)
Sheet	-0.44 (0.52) (N = 98)	-0.53 (0.50) (N = 15)	-	-	-
Loop	-1.40 (0.39) (N = 251)	-1.51 (0.38) (N = 236)	-	-1.71 (0.48) (N = 148)	-1.07 (0.49) (N = 129)

Supplementary Table 4. Molecular Dynamics checklist.

**Reliability and reproducibility checklist for molecular dynamics simulations** ***All boxes must be marked YES by acceptance unless "Response not needed if No".**		**Yes**	**No**	**Response (Please state where this information can be found in the text)**
**1. Convergence of simulations and analysis**				
1a. Is an evaluation presented in the text to show that the property being measured has equilibrated in the simulations (*e.g.* time-course analysis)?		☒	☐	SI figure 12
1b. Then, is it described in the text how simulations are split into equilibration and production runs and how much data were analyzed from production runs?		☒	☐	Material and methods section in the SI
1c. Are there at least 3 simulations per simulation condition with statistical analysis?		☒	☐	6 simulations per condition.
1d. Is evidence provided in the text that the simulation results presented are independent of initial configuration?		☒	☐	SI figure 12
**2. Connection to experiments**				
2a. Are calculations provided that can connect to experiments (*e.g.* loss or gain in function from mutagenesis, binding assays, NMR chemical shifts, J-couplings, SAXS curves, interaction distances or FRET distances, structure factors, diffusion coefficients, bulk modulus and other mechanical properties, *etc.*)?		☒	☐	Distances compare to ERP, conformations compare to cryo-EM and response to protonation compares to cryo-EM and biochemical data presented in Ghilarov et al, DOI: 10.1126/sciadv.abj5363
**3. Method choice**				
3a. Do simulations contain membranes, membrane proteins, intrinsically disordered proteins, glycans, nucleic acids, polymers, or cryptic ligand binding?		☒	☐	Membrane and membrane protein
3b. Is it described in the text whether the accuracy of the chosen model(s) is sufficient to address the question(s) under investigation (e.g. all-atom vs. coarse-grained models, fixed charge vs. polarizable force fields, implicit vs. explicit solvent or membrane, force field and water model, etc.)?		☒	☐	Methods section of the SI and SI Figure 13.
3c. Is the timescale of the event(s) under investigation beyond the brute-force MD simulation timescale in this study that enhanced sampling methods are needed?		☐	☒	Response not needed
	If **YES**, are the parameters and convergence criteria for the enhanced sampling method clearly stated?	☐	☐	Response not needed
	If **NO,** is the evidence provided in the text?	☐	☒	Limitations are described in the SI Figure 13
**4. Code and reproducibility**				

4a. Is a table provided describing the system setup that includes simulation box dimensions, total number of atoms, total number of water molecules, salt concentration, lipid composition (number of molecules and type)?	☐	☒	Response not needed
4b. Is it described in the text what simulation and analysis software and which versions are used?	☒	☐	In the Methods section of the SI
4c. Are other parameters for the system setup described in the text, such as protonation state, type of structural restraints if applied, nonbonded cutoff, thermostat and barostat, etc.?	☒	☐	Described in the Methods section of the SI, and a full record is uploaded to the zenodo server
4d. Are initial coordinate and simulation input files and a coordinate file of the final output provided as supplementary files or in a public repository?	☒	☐	Uploaded to the zenodo server (https://doi.org/10.5281/zenodo.17544057).
4e. Is there custom code or custom force field parameters?	☐	☒	Response not needed
If **YES**, are they provided as supplementary files or in a public repository?	☐	☐	Response not needed

Supplementary references

1 Wassenaar, T. A., Ingolfsson, H. I., Bockmann, R. A., Tieleman, D. P. & Marrink, S. J. Computational Lipidomics with insane: A Versatile Tool for Generating Custom Membranes for Molecular Simulations. *J Chem Theory Comput* **11**, 2144-2155 (2015). https://doi.org/10.1021/acs.jctc.5b00209

2 Wassenaar, T. A., Pluhackova, K., Bockmann, R. A., Marrink, S. J. & Tieleman, D. P. Going Backward: A Flexible Geometric Approach to Reverse Transformation from Coarse Grained to Atomistic Models. *J Chem Theory Comput* **10**, 676-690 (2014). https://doi.org/10.1021/ct400617g

3 Tironi, I. G., Sperb, R., Smith, P. E. & van Gunsteren, W. F. A generalized reaction field method for molecular dynamics simulations. *The Journal of Chemical Physics* **102**, 5451-5459 (1995). https://doi.org/10.1063/1.469273

4 Bussi, G., Donadio, D. & Parrinello, M. Canonical sampling through velocity rescaling. *J Chem Phys* **126**, 014101 (2007). https://doi.org/10.1063/1.2408420

5 Parrinello, M. & Rahman, A. Polymorphic transitions in single crystals: A new molecular dynamics method. *Journal of Applied Physics* **52**, 7182-7190 (1981). https://doi.org/10.1063/1.328693

6 Huang, J. *et al.* CHARMM36m: an improved force field for folded and intrinsically disordered proteins. *Nat Methods* **14**, 71-73 (2017). https://doi.org/10.1038/nmeth.4067

7 Abraham, M. J. *et al.* GROMACS: High performance molecular simulations through multi-level parallelism from laptops to supercomputers. *SoftwareX* **1-2**, 19-25 (2015). https://doi.org/https://doi.org/10.1016/j.softx.2015.06.001

8 Olsson, M. H., Sondergaard, C. R., Rostkowski, M. & Jensen, J. H. PROPKA3: Consistent Treatment of Internal and Surface Residues in Empirical pKa Predictions. *J Chem Theory Comput* **7**, 525-537 (2011). https://doi.org/10.1021/ct100578z

9 Aho, N. *et al.* Scalable Constant pH Molecular Dynamics in GROMACS. *J Chem Theory Comput* **18**, 6148-6160 (2022). https://doi.org/10.1021/acs.jctc.2c00516

10 Kohnke, B., Briand, E., Kutzner, C. & Grubmuller, H. Constant pH Simulation with FMM Electrostatics in GROMACS. (B) GPU Accelerated Hamiltonian Interpolation. *J Chem Theory Comput* **21**, 1787-1804 (2025). https://doi.org/10.1021/acs.jctc.4c01319

11 Briand, E., Kohnke, B., Kutzner, C. & Grubmuller, H. Constant pH Simulation with FMM Electrostatics in GROMACS. (A) Design and Applications. *J Chem Theory Comput* **21**, 1762-1786 (2025). https://doi.org/10.1021/acs.jctc.4c01318

12 Jeschke, G. *et al.* DeerAnalysis2006-A Comprehensive Software Package for Analyzing Pulsed ELDOR Data. *Appl Magn Reson* **30**, 473-498 (2006).

13 Fabregas Ibanez, L., Jeschke, G. & Stoll, S. DeerLab: a comprehensive software package for analyzing dipolar electron paramagnetic resonance spectroscopy data. *Magn Reson (Gott)* **1**, 209-224 (2020). https://doi.org/10.5194/mr-1-209-2020

14 Fabregas-Ibanez, L., Tessmer, M. H., Jeschke, G. & Stoll, S. Dipolar pathways in dipolar EPR spectroscopy. *Phys Chem Chem Phys* **24**, 2504-2520 (2022). https://doi.org/10.1039/d1cp03305k

15 Teucher, M. & Bordignon, E. Improved signal fidelity in 4-pulse DEER with Gaussian pulses. *J Magn Reson* **296**, 103-111 (2018). https://doi.org/10.1016/j.jmr.2018.09.003
