# Supplementary material for: Shared structural mechanisms of alternating access between the secondary peptide transporter SbmA and ABC transporters
Source: Nat Commun. 2026 Apr 15;17:5619. doi: 10.1038/s41467-026-71633-3 (PMC13316013; doi:10.1038/s41467-026-71633-3)
Supplement: Supplementary file 2 — Reporting Summary [file 41467_2026_71633_MOESM2_ESM.pdf]

nature portfolio

Corresponding author(s): Dmitry Ghilarov , Christos Pliotas , Thomas Stockner, Dirk J. Slotboom, Konstantinos Beis

Last updated by author(s): Feb 23, 2026

Reporting Summary

Nature Portfolio wishes to improve the reproducibility of the work that we publish. This form provides structure for consistency and transparency in reporting. For further information on Nature Portfolio policies, see our Editorial Policies and the Editorial Policy Checklist.

Statistics

For all statistical analyses, confirm that the following items are present in the figure legend, table legend, main text, or Methods section.

n/a	Confirmed	
☐	☒	The exact sample size (n) for each experimental group/condition, given as a discrete number and unit of measurement
☒	☐	A statement on whether measurements were taken from distinct samples or whether the same sample was measured repeatedly
☒	☐	The statistical test(s) used AND whether they are one- or two-sided<br>*Only common tests should be described solely by name; describe more complex techniques in the Methods section.*
☒	☐	A description of all covariates tested
☒	☐	A description of any assumptions or corrections, such as tests of normality and adjustment for multiple comparisons
☐	☒	A full description of the statistical parameters including central tendency (e.g. means) or other basic estimates (e.g. regression coefficient) AND variation (e.g. standard deviation) or associated estimates of uncertainty (e.g. confidence intervals)
☒	☐	For null hypothesis testing, the test statistic (e.g. F, t, r) with confidence intervals, effect sizes, degrees of freedom and P value noted<br>*Give P values as exact values whenever suitable.*
☒	☐	For Bayesian analysis, information on the choice of priors and Markov chain Monte Carlo settings
☒	☐	For hierarchical and complex designs, identification of the appropriate level for tests and full reporting of outcomes
☒	☐	Estimates of effect sizes (e.g. Cohen's d, Pearson's r), indicating how they were calculated

Our web collection on statistics for biologists contains articles on many of the points above.

Software and code

Policy information about availability of computer code

Data collection	cryo-EM data collection using EPU (eBIC), BRUKER ELEXSYS E580 pulsed spectrometer operating at Q-band (34 GHZ) with a 150 W TWT Q-band amplifier and a probe head supporting a cylindrical resonator ER 5106QT-2w
Data analysis	cryoSPARC , phenix, COOT, Chimera X, DeerAnalysis202, charmm36 force field , GROMACS version 2019.2

For manuscripts utilizing custom algorithms or software that are central to the research but not yet described in published literature, software must be made available to editors and reviewers. We strongly encourage code deposition in a community repository (e.g. GitHub). See the Nature Portfolio guidelines for submitting code & software for further information.

Data

Policy information about availability of data

All manuscripts must include a data availability statement. This statement should provide the following information, where applicable:

- Accession codes, unique identifiers, or web links for publicly available datasets
- A description of any restrictions on data availability
- For clinical datasets or third party data, please ensure that the statement adheres to our policy

An interactive version of the phylogenetic tree of SbmA and related ABC transporter proteins is available via Microreact: https://microreact.org/project/sbma. Cryo-EM density maps, half maps, and masks have been deposited in the Electron Microsco-py Data Bank (EMDB); SbmA-Fab inward-facing-wide EMD-51036 [https://www.ebi.ac.uk/emdb/EMD-51036], SbmA-Fab inward-facing-narrow EMD-51037 [https://www.ebi.ac.uk/emdb/EMD-51037], SbmA-Sb2 inward-facing-narrow with

2 Sb2 EMD-50994 [https://www.ebi.ac.uk/emdb/EMD-50994], SbmA-Sb2 in-ward-facing-wide with 1 Sb2 EMD-50995 [https://www.ebi.ac.uk/emdb/EMD-50995], SbmA-Sb2 inward-facing-wide with 2 Sb2 EMD-50996 [https://www.ebi.ac.uk/emdb/EMD-50996], SbmA inward-facing-wide EMD-50997 [https://www.ebi.ac.uk/emdb/EMD-509967], SbmA inward-facing-occluded EMD-50998 [https://www.ebi.ac.uk/emdb/EMD-50998]. The atomic coordinates have been deposited in the Protein Data Bank (PDB) under accession codes 9G4E [https://doi.org/10.2210/pdb9G4E/pdb] (SbmA-Fab, inward-facing-wide), 9G4F [https://doi.org/10.2210/pdb9G4F/pdb] (SbmA-Fab, inward-facing-narrow), 9G3D [https://doi.org/10.2210/pdb9G3D/pdb] (SbmA-Sb2, inward-facing-narrow with 2 Sb2), 9G3E [https://doi.org/10.2210/pdb9G3E/pdb] (SbmA-Sb2, inward-facing-wide with 2 Sb2), 9G3F [https://doi.org/10.2210/pdb9G3F/pdb] (SbmA, inward-facing-wide), and 9G3G [https://doi.org/10.2210/pdb9G3G/pdb] (SbmA, inward-facing-occluded). Raw movies have been deposited in the Electron Microscopy Public Im-age Archive (EMPIAR) under accession numbers EMPIAR-12192 [https://doi.org/10.6019/EMPIAR-12192] (SbmA-Fab, inward facing wide and in-ward-facing-narrow), 12888 (SbmA-Sb2), EMPIAR-12889 [https://doi.org/10.6019/EMPIAR-12889] (SbmA, inward-facing-wide) and EMPIAR-12890 [https://doi.org/10.6019/EMPIAR-12890] (inward-facing-occluded). The pre-viously resolved structure of SbmA in the outward-open conformation and the struc-tures of MsbA in the outward- and inward-facing conformation used in this study is available through the PDB under the accession codes 7P34 [https://doi.org/10.2210/pdb7P34/pdb], 8TSO [https://doi.org/10.2210/pdb8TSO/pdb], and 7MEW [https://doi.org/10.2210/pdb7MEW/pdb], respectively. The AlphaFold model of YddA used in this study is available through the AlphaFold Protein Struc-ture Database under the accession code AF-P31826-F1 [https://alphafold.ebi.ac.uk/entry/P31826]. All starting systems used in MD simula-tions, the simulations parameters, trajectory data and analysis plots are available at zenodo with ID 17544057 [https://doi.org/10.5281/zenodo.17544057]).

Research involving human participants, their data, or biological material

Policy information about studies with human participants or human data. See also policy information about sex, gender (identity/presentation), and sexual orientation and race, ethnicity and racism.

Reporting on sex and gender	n/a
Reporting on race, ethnicity, or other socially relevant groupings	n/a
Population characteristics	n/a
Recruitment	n/a
Ethics oversight	n/a

Note that full information on the approval of the study protocol must also be provided in the manuscript.

Field-specific reporting

Please select the one below that is the best fit for your research. If you are not sure, read the appropriate sections before making your selection.

☒ Life sciences ☐ Behavioural & social sciences ☐ Ecological, evolutionary & environmental sciences

For a reference copy of the document with all sections, see nature.com/documents/nr-reporting-summary-flat.pdf

Life sciences study design

All studies must disclose on these points even when the disclosure is negative.

Sample size	Sample sizes are not relevant for structural work.
Data exclusions	no data exclusions
Replication	MD simulations were repeated in replicates. All replicate data are provided in SI.
Randomization	EPR data cannot be randomised as they were from different mutants that otherwise could not be identified.
Blinding	EPR data cannot be blinded as they were from different mutants that otherwise could not be identified.

Reporting for specific materials, systems and methods

We require information from authors about some types of materials, experimental systems and methods used in many studies. Here, indicate whether each material, system or method listed is relevant to your study. If you are not sure if a list item applies to your research, read the appropriate section before selecting a response.

Materials & experimental systems

n/a	Involved in the study
☐	☒ Antibodies
☒	☐ Eukaryotic cell lines
☒	☐ Palaeontology and archaeology
☒	☐ Animals and other organisms
☒	☐ Clinical data
☒	☐ Dual use research of concern
☒	☐ Plants

Methods

n/a	Involved in the study
☒	☐ ChIP-seq
☒	☐ Flow cytometry
☒	☐ MRI-based neuroimaging

Antibodies

Antibodies used	Antibodies were isolated by So Iwata (FabS11-1)
Validation	ELISA and sequencing, cryo-EM structure

Plants

Seed stocks	n/a
Novel plant genotypes	n/a
Authentication	n/a
