# Supplementary material for: Shared structural mechanisms of alternating access between the secondary peptide transporter SbmA and ABC transporters
Source: Nat Commun. 2026 Apr 15;17:5619. doi: 10.1038/s41467-026-71633-3 (PMC13316013; doi:10.1038/s41467-026-71633-3)
Supplement: Supplementary file 3 — Transparent Peer Review File [file 41467_2026_71633_MOESM3_ESM.pdf]

nature portfolio

Peer Review File

Shared structural mechanisms of alternating access between the secondary peptide transporter SbmA and ABC transporters

Corresponding Author: Dr Konstantinos Beis

This file contains all reviewer reports in order by version, followed by all author rebuttals in order by version.

Version 0:

Reviewer comments:

Reviewer #1

(Remarks to the Author)
Beis et al. have presented cryo-EM structures of SbmA in several inward-facing states, primarily focusing on the inward-facing occluded, inward-facing narrow, and inward-facing wide states, expanding upon their previous structure of the outward-open state. Additionally, the authors explore the conformational dynamics of SbmA using DEER spectroscopy and MD simulations. Overall, the manuscript is well organized, and the findings are of interest. However, as the cryo-EM structure and DEER spectroscopy are outside my area of expertise, I am unable to fully evaluate these sections. As a computational biophysicist, I have concentrated on the MD-related aspects of the study in providing my comments. While the manuscript presents valuable insights, I believe it may not yet meet the high standards expected by Nature Communications in its current form.

1. The manuscript describes the construction of seven models, including the outward-open state, the inward-facing wide state, and five protonated models of the outward-open state incorporating glutamate residues. However, the rationale for selecting the five glutamate residues (E193, E203, E269, E276, and E378) for protonation is not adequately explained. Given the pKa predictions shown in Supplementary Fig. 12, the exclusion of E103 (which might be a relevant residue based on the pKa analysis) and the inclusion of E276 and E378, which have relatively low pKa values, are not fully justified. A more detailed explanation of these choices, ideally based on prior experimental data, their structural significance, or potential roles in the transport mechanism, would strengthen the rationale for these decisions.

2. The protonation states of the outward-open and inward-facing wide models are not clearly defined. It remains unclear whether all glutamate residues are unprotonated in both models. This lack of clarity makes it challenging for readers to fully understand the basis of the simulations and their implications. Clearer definitions of the protonation states for these models are essential.

3. To provide a more comprehensive understanding of the role of glutamate protonation in different conformational states, it would be beneficial to also include Glu-protonation models starting from the inward-facing wide state.

4. I recommend summarizing key details of the models (such as initial structures, protonation states, etc.) and simulations (simulation length, force fields used, repeats, etc.) in a table. This would enhance the manuscript's readability and clarity, allowing readers to quickly grasp the essential information related to the computational models and simulations.

5. The manuscript may overinterpret some of the MD simulation data. For example, the statement regarding the different distance distributions upon protonation of various glutamate residues is not robust. The wide distribution observed for the E193-protonated model was primarily driven by one of the three simulations, which raises concerns about the reliability and reproducibility of this observation. Additionally, it remains unclear whether the distance distributions from the MD simulations have reached convergence.

6. Based on the RMSD trajectories shown in Supplementary Fig. 12, it appears that the simulations may not have been well-equilibrated during the first 100 ns. It is important to clarify whether the distance distributions shown in Fig. 5 and Supplementary Fig. 12 were derived from the entire trajectory or from the portion of the trajectory after equilibration. To

ensure the accuracy of the results, calculations should be based solely on equilibrated data.

7. To address the potential issue of non-converged distributions, I recommend employing enhanced sampling techniques such as metadynamics or umbrella sampling. These methods would improve the sampling of the conformational space, leading to more reliable and converged results, and would enhance the robustness of the conclusions drawn from the MD simulations.

8. The calculated distance distributions from DEER spectroscopy do not perfectly match those derived from the cryo-EM structure. Although the manuscript does not explore the source of this discrepancy in detail, it could be partially from the algorithm (here, MMM) used for calculating distances from the cryo-EM structure. To assess the reliability of the distance calculations, it would be beneficial to compare the results with those obtained from other algorithms, such as DEER-PREdict.

9. A potentially valuable addition to the work would be the application of constant pH molecular dynamics (MD) simulations. These simulations would allow for a more dynamic representation of the protonation states, particularly for residues like glutamates, whose protonation state may change across different conformational states. Constant pH MD could provide a more detailed and physically accurate model of the system by capturing protonation transitions during the simulations, which may further illuminate the role of protonation in the conformational dynamics of SbmA. I suggest discussing this approach in the context of future work or as a potential extension of the current study.

10. Other minor points:
1) Figure 5: The figure could benefit from improvements in both its design and the accompanying figure legends. The legends should provide more detailed information to enhance clarity.
2) Line 380: There is a typographical error (“is is”) that should be corrected.
3) Line 909: The term “charmm36” should be rephrased to “CHARMM36m” to ensure accuracy.
4) Line 885: The unit of the force constant (100) should be specified for clarity.

Reviewer #2

(Remarks to the Author)
The study by Ettema et al. reports several Cryo-EM structures of SbmA, a membrane transporter that imports antimicrobial peptides, in occluded and inward-facing states.The work is highly significant, as it bridges the gap between ABC transporters and secondary transporters, providing insights into the evolution and proton-driven transport mechanism of SbmA. The findings are novel and contribute to the broader understanding of membrane transport proteins. However, there are areas where the paper could be improved to strengthen its impact and clarity.

Major concerns:
1) While the paper proposes that protonation of specific glutamate residues drives conformational changes, the exact sequence of protonation events and how they propagate through the glutamate ladder is not fully elucidated. The authors could perform additional MD simulations or experimental studies (e.g., pH-dependent EPR measurements) to map the protonation pathway and validate the role of specific glutamate residues in the transport cycle.
2) The evolutionary link between SbmA and YddA is based on sequence and structural similarity but lacks experimental validation (e.g., functional complementation studies). The authors could perform functional complementation experiments in a YddA-deficient strain to test whether SbmA can rescue the phenotype, providing stronger evidence for their evolutionary hypothesis, if possible.

Minor concerns:
1) Some sections of the paper are overly complex, with long sentences and technical jargon that may hinder readability. Additionally, some figures (e.g., Figure 4) could be better annotated to guide the reader. The authors should simplify the language, break down complex sentences, and improve figure annotations to make the paper more accessible to a broader audience.
2) Some abbreviations (e.g., SLiPT, TMD) are used without explanation when first introduced. Ensure all abbreviations are defined at first use.
3) PDB accession codes for cryo-EM structures should be provided.
4) Some figures (e.g., Figure 2) could benefit from higher resolution and clearer labels to highlight key structural features.

Reviewer #3

(Remarks to the Author)
The paper by Ettema et al. presents an elegant interdisciplinary work which addresses the evolution of SbmA-like transporters. Using bioinformatics analysis the authors found that the SbmA-like transporters have evolved from an ancestor of the type IV ABC transporter YddA, and then they could demonstrate that the conformational transitions of Type IV ABC transporters have also been propagated in SbmA using cryo-EM. The EPR analysis and the MD simulations provides information which aid the proposed pH-dependent mechanism of action. The conclusions are sound, and the work should be published, but there are several issues related to the EPR analysis that should be addressed before publication.
Issues to address (EPR analysis)

1) To facilitate the understanding of the data presented in Table 1, it would be good to highlight in the structures on the first column the pairs of amino acids (connected by a dotted line) for which the three distances are listed in this table. Why the periplasmic pair 349-349 (presented in Fig. 2) was not included in the table? Adding the membrane lines to highlight the transmembrane region on the structures (as in Fig. 2) would also increase the clarity of the representation. In addition, the Ca-Ca distances are given with sub-Angstrom resolution, but they could be better rounded without digit after the comma because sub-Angstrom resolution is not accurate or necessary here.
2) The authors should comment on why DEER/PELDOR data were only obtained in detergent-solubilized samples and not in nanodiscs. The data in nanodiscs could have added additional information on the conformational ensemble providing a direct correlation to the cryo-EM structures, especially because the outward facing conformation could only be populated in nanodiscs at pH 7.5 with 2 Fabs.
3) Fig 2: the authors show surface maps for 4 states in b but in c (there is also a typo, they name c as b in the legend) only 3 views are shown. The inward facing narrow conformation from b should also be presented in c.
4) Fig 4: the simulated distance distribution is misplaced below the experimental data. The legend is very long and could be shortened (e.g. part of the description can go to the M&M). The simulation on the inward-facing narrow shown in Fig. S11 should be added for completeness directly in Fig. 4 (as well as the structural model), to highlight the similarity between the distances in different states. It is otherwise difficult to follow the interpretation: ‘At pH 4.5 the narrow distance distribution is is most consistent with the inward-facing-narrow state (Supplementary Fig. 11) with some contribution from one or more states with structures closer to the inward-occluded state.’
5) Interpretation of CW data from Fig 4: “The EPR spectral lines 388 showed large differences when recorded at different pH values, indicating that both 389 H127R1 and T294R1 become highly rigid at pH 4.5 compared to pH 8 and 6.” Some spectral changes are visible at pH 4.5 (yet the spectrum is not in the rigid limit), but there is also a “rigid” component already present at pH 8 and 6. The spectra should be compared in the SI (amplitude normalized), to better show the differences. Note: The baseline of the spectrum at pH 4.5 in panel a seems not to be properly corrected and the high field peak results non-symmetric. Another concern is that the authors used MTSL, which can get released from the protein upon disulfide bond breakage. The authors should confirm that the very mobile component seen at pH 6 and 8 (main difference to spectra at pH 4.5) is not arising from partially released label. The conclusive statement: ‘Collectively, the CW-EPR and PELDOR data suggests that acidic pH rigidifies both peri- and cytoplasmic gate regions of SbmA and pushes the SbmA conformational ensemble towards the inward-occluded state.’ is not fully corroborated by the cw data and by the PELDOR data in Fig. 4d.
6) It is not clear why the modulation depth of the DEER traces in Figure 4 (especially in panel f: 20%, 10%, 25% for pH 8,6,4.5) varies among the different pH values. The authors should comment on that. If the mobile component in the cw spectra correlates with the lower modulation, it is tempting to suggest that the mobile component is due to label release. Otherwise, another possible explanation should be provided.
7) One set of DEER data at different pH values is shown for two pairs of labels (127 and 294). However, at least a biological repeat should be shown, to corroborate the changes observed.
8) DEER data from Fig. 4: in accordance with the guidelines form the DEER/PELDOR community (Schiemann et al., J. Am. Chem. Soc. 2021, 143, 43, 17875–17890), the authors should show another, user-unbiased, analysis for validation of the distance distributions, e.g. neural network, and present it in SI. In the M&M, the information about the validation of the Tikhonov analysis is also missing (which model for the background, which range of parameters were validated).
9) The assignment of the outward open conformation based on the PELDOR data (Fig. 4d) is ambiguous. There might be still some residual 2+1 signals at the end of the PELDOR traces that could interfere with the determination of the long-distance peaks. The V(t) traces of Fig. 4 clearly show an increase in intensity towards the end, which is then fitted in the F(t) analysis. Indeed, the authors cut the last 600 ns of the traces because of the 2+1 artefact, but it is possibly not sufficient. The primary DEER data before the 600 ns cut must be shown in the SI. To verify if the oscillations at the end of the trace are real or due to residual 2+1 signal, it is necessary to detect traces with shorter length and compare them with the longer traces (without 600 ns cut) to identify is the 2+1 signal moves. Alternatively, softer pump pulses can be used to verify if the oscillations at the end of the trace persist or the software DeerLab can be used to calculate the 2+1 signal. If the 2+1 signal is not ruled out completely, it is not possible to state that: ‘At pH 6, and even more at pH 8, main distance components consistent with the outward open SbmA state are also present, although the PELDOR data suggest that these species may only constitute a minor population within the SbmA ensemble’.

Reviewer #4

(Remarks to the Author)
I co-reviewed this manuscript with one of the reviewers who provided the listed reports. This is part of the Nature Communications initiative to facilitate training in peer review and to provide appropriate recognition for Early Career Researchers who co-review manuscripts.

Version 1:

Reviewer comments:

Reviewer #1

(Remarks to the Author)
The authors indicate that the five glutamate residues (E193, E203, E269, E276, E378) selected for protonation were chosen based on "previous structural and functional work." However, they do not explicitly link this selection to their own experimental evidence—for example, which of these glutamate mutations most severely impaired SbmA’s transport activity, or how the residues’ positions in the transmembrane domain align with the proposed proton translocation path (defined as

the "glutamate ladder" in Supplementary Fig. 10). Additionally, the authors exclude E103 from protonation state testing—even though it exhibits a significant pKa shift due to its location—while claiming it cannot participate in membrane-spanning proton transport; this reasoning is unconvincing, as allosteric effects and dynamic influences cannot be ruled out.

The authors propose a "logical movement" of protons from E203 to E193 to drive conformational change, yet this protonation sequence is inferred only from residue location, rather than being supported by experimental or computational data. While the authors note that inward-facing protonation simulations were conducted, they provide no details on how these simulations either alter or confirm the proposed protonation sequence. As a result, the core mechanism connecting protonation to conformational change remains speculative.

The authors use the full 1 μs trajectory (per simulation repeat; Supplementary Table 1) for their analysis—including initial trajectories where RMSD values are still rising (Supplementary Fig. 12), a period that indicates non-equilibrium states. They fail to provide quantitative evidence demonstrating that the simulations reached equilibrium, which raises questions about whether early non-equilibrated data skews distance distributions and undermines the validity of their conclusions.

The authors justify their rejection of enhanced sampling techniques (e.g., metadynamics, umbrella sampling) by citing "prohibitively large collective variables". But they do not address other alternative advanced methods that could improve conformational sampling.

The authors dismiss CpHMD, claiming it suffers from "significant error in correctly predicting pKa values of buried amino acids." This argument is unpersuasive: the buried glutamates in the glutamate ladder (e.g., E193, E203) are not isolated from solvent (as shown in Supplementary Fig. 10), and modern CpHMD protocols— which the authors neither cite nor discuss—have improved accuracy for such residues. While CpHMD has inherent limitations, it remains a key approach for validating pH-dependent protonation dynamics. Their dismissal thus appears to reflect a simple reluctance to invest additional effort.

Reviewer #2

(Remarks to the Author)
The authors have successfully addressed all the concerns raised during the previous round of revision.

Reviewer #3

(Remarks to the Author)
The revised version of the manuscript has addressed all of the issues we had identified and the clarity of the manuscript has improved. I therefore recommend publication in Nature Comm.

There are few minor remaining issues to be addressed:

- Fig. 4 d is still missing the inward-facing narrow simulation or there is some problem with color rendering which made cyan invisible since the inward-facing wide is plotted blue instead of violet.
- The authors still say in the text "The CW EPR spectral lines 388 showed large differences when recorded at different pH values, indicating that both 389 H127R1 and T294R1 become highly rigid at pH 4.5 compared to pH 8 and 6. " However, the immobile component is already present at pH 8 and 6, 'highly rigid' is an overstatement, because the spectra are not yet in the rigid limit for EPR (rotational motion slower than 30 ns). The sentence can be reformulated addressing the fact that the rotational motion at the chosen sites contains multiple components at all pH values, and that the lowest pH correlates with lower dynamics. This is a more general description of the spectra, which avoid overinterpretation of the CW data.
- Concerning label release, the authors explained in detail the steps taken to ensure that the sample contains the least amount of free label, and indeed the area under the very mobile component is very small, therefore it will not largely influence the observed modulation depth in the DEER traces.
- Fig 5: the authors need to add to the title that these are MD-obtained distributions and also add to the legend the information about the inward-facing conformation they chose to perform the simulations (-wide, I assume from line 415, but in line 409 it is not specified).
- Methods: EPR: the authors described EDFS measurements, which were never shown. EPR Modeling: it is important to say which MMM version (and Matlab) was used since the same libraries might give slightly different outputs in older versions.
- Few typos to be corrected: CW: CW EPR, space missing, kHz not KHz.

Reviewer #4

(Remarks to the Author)
I co-reviewed this manuscript with one of the reviewers who provided the listed reports. This is part of the Nature Communications initiative to facilitate training in peer review and to provide appropriate recognition for Early Career Researchers who co-review manuscripts.

Version 2:

Reviewer comments:

Reviewer #1

(Remarks to the Author)
Thank you to the authors for their efforts in revising the manuscript. I fully recognize and appreciate that this work integrates insights from multiple techniques, and I find the overall study valuable. However, I remain unsatisfied with the quality of the MD simulations—particularly the authors' claim that these simulations are "well-converged." For example, the low-populated peaks in the Y368-Y368' distance distribution are derived entirely from one of the six simulation repeats. I cannot accept this as evidence to support the assertion of well-converged simulations, and I do not believe the revision adequately addressed this core concern. I acknowledge that MD simulations are not the central contribution of this project, and thus urge the authors to avoid overstating their conclusions based on MD.

Regarding CpHMD, I understand that conducting such additional work would be resource-intensive, and I am not insisting on its implementation. Nevertheless, I recommend that the authors incorporate discussions on the potential application of more advanced computational methods to address similar research questions, along with relevant citations. This addition would enhance the manuscript's utility for readers interested in the broader methodological context.

Version 3:

Reviewer comments:

Reviewer #1

(Remarks to the Author)
Thanks for refining the manuscript and refraining from overinterpreting the MD data. I have no further comments.

Open Access This Peer Review File is licensed under a Creative Commons Attribution 4.0 International License, which permits use, sharing, adaptation, distribution and reproduction in any medium or format, as long as you give appropriate credit to the original author(s) and the source, provide a link to the Creative Commons license, and indicate if changes were made.
In cases where reviewers are anonymous, credit should be given to 'Anonymous Referee' and the source.
The images or other third party material in this Peer Review File are included in the article's Creative Commons license, unless indicated otherwise in a credit line to the material. If material is not included in the article's Creative Commons license and your intended use is not permitted by statutory regulation or exceeds the permitted use, you will need to obtain permission directly from the copyright holder.
To view a copy of this license, visit https://creativecommons.org/licenses/by/4.0/

We would like to thank all the Reviewers for their positive feedback and constructive comments. We have addressed all their queries below:

Reviewer 1:

1. The manuscript describes the construction of seven models, including the outward-open state, the inward-facing wide state, and five protonated models of the outward-open state incorporating glutamate residues. However, the rationale for selecting the five glutamate residues (E193, E203, E269, E276, and E378) for protonation is not adequately explained. Given the pKa predictions shown in Supplementary Fig. 12, the exclusion of E103 (which might be a relevant residue based on the pKa analysis) and the inclusion of E276 and E378, which have relatively low pKa values, are not fully justified. A more detailed explanation of these choices, ideally based on prior experimental data, their structural significance, or potential roles in the transport mechanism, would strengthen the rationale for these decisions.

The glutamates were selected based on our previous structural and functional work. We have now listed the residues that form the 'glutamate ladder'. As it is currently unclear, which glutamate residues are protonated, we have tested all glutamate residues within the transportation path. This choice is linked to the experimental data showing that SbmA is a proton gradient driven transporter. E103 is a potentially interesting candidate, but its location precludes any bound proton from being transported through the cell membrane, it can thus not serve as a glutamate that could temporarily accept a transported proton.

Propka based pKa calculations are very useful, but are currently also limited by a large uncertainty. Thus, instead of relying on solely these calculations, we instead opted to investigate all glutamates of the 'glutamate ladder' in the path of substrate and proton translocation.

2. The protonation states of the outward-open and inward-facing wide models are not clearly defined. It remains unclear whether all glutamate residues are unprotonated in both models. This lack of clarity makes it challenging for readers to fully understand the basis of the simulations and their implications. Clearer definitions of the protonation states for these models are essential.

We have refined the definition, to make it clearer. In a nutshell, we always protonated two glutamates, each time one per SbmA protomer. We thus have 6 different protonation states: unprotonated, E193+E193', E203+E203', E269+E269', E276+E276', and E378+E378'). We carried out the same set of protonation state simulations for the outward-facing and the inward-facing conformations, thus in total investigating 12 system settings.

3. To provide a more comprehensive understanding of the role of glutamate protonation in different conformational states, it would be beneficial to also include Glu-protonation models starting from the inward-facing wide state.

We now present simulation data of all protonation states in the outward and the inward-facing conformation. The overall production simulations time of all simulations is 72 µs.

4. I recommend summarizing key details of the models (such as initial structures, protonation states, etc.) and simulations (simulation length, force fields used, repeats, etc.) in a table. This would enhance the manuscript's readability and clarity, allowing readers to quickly grasp the essential information related to the computational models and simulations.

Many thanks for the suggestion. We have now added a table summarizing all simulations that we carried out in Supplementary Table 1.

**SbmA conformation**	**unprotonated**	**E193+E193'**	**E203+E203'**	**E269+E269'**	**E276+E276'**	**E378+E378'**
inward-facing	n=6, (1 µs)	n=6, (1 µs)	n=6, (1 µs)	n=6, (1 µs)	n=6, (1 µs)	n=6, (1 µs)
outward-facing	n=6, (1 µs)	n=6, (1 µs)	n=6, (1 µs)	n=6, (1 µs)	n=6, (1 µs)	n=6, (1 µs)

5. The manuscript may overinterpret some of the MD simulation data. For example, the statement regarding the different distance distributions upon protonation of various glutamate residues is not robust. The wide distribution observed for the E193-protonated model was primarily driven by one of the three simulations, which raises concerns about the reliability and reproducibility of this observation. Additionally, it remains unclear whether the distance distributions from the MD simulations have reached convergence.

We appreciate the concern. We have thus doubled the number of simulations and the length of the simulations. The main conclusion did not change with the extended dataset, but becomes statistically more robust, supporting the notion that the simulations are converged for the respective states. The data suggest that some of the protonated systems could be metastable, while E193 is unstable in the outward-facing state. The mechanistic interpretation of the glutamate ladder is that a proton is most likely passed on from glutamate to glutamate until reaching E193. The moment of proton passing is unclear, but the current results indicate that extensively longer simulation (much longer than the current simulations) for protonated glutamate residues in the middle of the glutamate ladder, thus at intermediate steps of proton passing towards E193, would drive the transport out of the correct distribution, because the proton cannot unbind in a classical force field representation as we would expect by being passed on step by step through the glutamate ladder. Given

this limitation, simulations longer than the current dataset might thus not improve the quality of the data.

6. Based on the RMSD trajectories shown in Supplementary Fig. 12, it appears that the simulations may not have been well-equilibrated during the first 100 ns. It is important to clarify whether the distance distributions shown in Fig. 5 and Supplementary Fig. 12 were derived from the entire trajectory or from the portion of the trajectory after equilibration. To ensure the accuracy of the results, calculations should be based solely on equilibrated data.

Production start after completing system equilibration simulations. The averages are derived from the six simulations of the entire production trajectory of 1 μs each. The systems remain within a state (except for E193), thus, applying statistical thermodynamics arguments, the entire trajectory should be relevant. The initial rise of the RMSD followed by reaching of a plateau value is expected and typical for a dynamic system moving within an energy basin. The value of the RMSD is a collective parameter that primarily depends on the number of residues and the shape of the energy basis of the simulated conformational state. Due to the dynamic nature, each atom moves a bit, adding to the sum of deviations from the starting conformation, until oscillating around the conformational state specific average distance.

7. To address the potential issue of non-converged distributions, I recommend employing enhanced sampling techniques such as metadynamics or umbrella sampling. These methods would improve the sampling of the conformational space, leading to more reliable and converged results, and would enhance the robustness of the conclusions drawn from the MD simulations.

We would like to disagree with the Reviewer. Metadynamics or umbrella sampling are two well established methods, with Metadynamics having seen several different implementations. They have strong merits, but also important limitations. We think that their use is not appropriate for the current system. The general issue for employing these types of enhanced sampling methods is the definition of the collective variables or essential degrees of freedom. The general shortcoming, as for example shown by the Parrinello group (see e.g. 10.1002/wcms.31), is that missing out on essential degrees of freedom in a Metadynamics simulation leads to an apparent energy barrier which in reality does not exist. An analysis of the important collective variables of the equilibrium simulations indicated that a prohibitively large number of degrees of freedom are relevant, which goes beyond the few collective variables that Metadynamics can efficiently handle, also leading to a computational burden. A similar challenge exists for umbrella sampling, which is primarily focused on being applied to only one single dimension. We therefore opted to rely on extensive unbiased simulations to avoid these shortcomings.

8. The calculated distance distributions from DEER spectroscopy do not perfectly match those derived from the cryo-EM structure. Although the manuscript does not explore the source of this discrepancy in detail, it could be partially from the algorithm (here, MMM) used for calculating distances from the cryo-EM structure. To assess the reliability of the distance calculations, it would be beneficial to compare the results with those obtained from other algorithms, such as DEER-PREdict.

We thank the Reviewer for their comment. We would like to highlight that the cryo-EM structures represent high-resolution 'snapshots' of the most stable or dominant conformations derived from the structure solution and refinement process. Therefore, the distances calculated by software used to model DEER/PELDOR distributions (MMM presented here, but similarly MtsslWizard (Hagelueken et al., Appl Magn Reson, 2012), which we also used to cross-check, reflect these static 'snap-shot' structures. Notably, the experimental DEER distributions show an ***ensemble*** of SbmA conformational states present in the protein samples. Although DEER can identify distances captured by the cryo-EM structures (i.e. with similar mean distance distribution distances overall in good agreement), it also detects all other states present in the ensemble, such as lowly populated states, which may have been missed by single particle cryo-EM during particle picking and refinement process. In that respect, DEER provides a more accurate and biologically relevant representation of the complete SbmA ensemble, while it also describes the dynamics SbmA undertakes to fulfil its functional role, even in cases when the particular states are not fully resolved. Importantly, we report significant population equilibrium shifts observed in DEER spectroscopy, which are consistent with the different states determined by cryo-EM, highlighting the valuable complementary nature of these two techniques in capturing both static and dynamic aspects of macromolecular structures.

9. A potentially valuable addition to the work would be the application of constant pH molecular dynamics (MD) simulations. These simulations would allow for a more dynamic representation of the protonation states, particularly for residues like glutamates, whose protonation state may change across different conformational states. Constant pH MD could provide a more detailed and physically accurate model of the system by capturing protonation transitions during the simulations, which may further illuminate the role of protonation in the conformational dynamics of SbmA. I suggest discussing this approach in the context of future work or as a potential extension of the current study.

Constant pH MD simulations in explicit solvent, while having theoretical merits of being a physically more accurate model, come with strong limitations. One key issue is the significant error in correctly predicting pKa values of buried amino acids, for example because of undersolvation, overstabilization, or time scales of the protonation-dependent conformation changes. For SbmA, the most interesting glutamate residues are all deeply buried in the protomer interface. We can thus not safely assume that constant pH MD simulations would improve the predictions. In addition, constant pH simulations are computational very demanding to achieve

convergence, especially if linked to conformational changes induced by protonation/deprotonation, which are slow on the MD timescale.

10. Other minor points:
1) Figure 5: The figure could benefit from improvements in both its design and the accompanying figure legends. The legends should provide more detailed information to enhance clarity.
2) Line 380: There is a typographical error ("is is") that should be corrected.
3) Line 909: The term "charmm36" should be rephrased to "CHARMM36m" to ensure accuracy.
4) Line 885: The unit of the force constant (100) should be specified for clarity.

These points have been corrected

Reviewer 2:

Major concerns:
1) While the paper proposes that protonation of specific glutamate residues drives conformational changes, the exact sequence of protonation events and how they propagate through the glutamate ladder is not fully elucidated. The authors could perform additional MD simulations or experimental studies (e.g., pH-dependent EPR measurements) to map the protonation pathway and validate the role of specific glutamate residues in the transport cycle.

We would like to disagree with the Reviewer. In our Science Advances manuscript (10.1126/sciadv.abj5363), we mutated the glutamate ladder, and all mutants either render the transporter inactive or reduced activity suggesting a very tight interplay of proton binding and movement. The MD and EPR have been performed with different protonation states and pHs, respectively, and provide a likely explanation on the proton movement. Considering the location of the glutamates along the glutamate ladder, mutagenesis, MD and EPR data, the logical movement is from E203 that propagates along to E193 to induce the conformational changes associated with the inward-facing conformation.

2) The evolutionary link between SbmA and YddA is based on sequence and structural similarity but lacks experimental validation (e.g., functional complementation studies). The authors could perform functional complementation experiments in a YddA-deficient strain to test whether SbmA can rescue the phenotype, providing stronger evidence for their evolutionary hypothesis, if possible.

This is a very good suggestion but in our previous work, when we made the *ΔsbmA*, YddA was unable to compensate for the lack of functional SbmA (10.1126/sciadv.abj5363). Similarly, previous studies that had identified SbmA mutants, the YddA did not compensate for loss of functional SbmA. We believe that although SbmA has evolved from YddA, there is no substrate overlap.

Minor concerns:

1）Some sections of the paper are overly complex, with long sentences and technical jargon that may hinder readability. Additionally, some figures (e.g., Figure 4) could be better annotated to guide the reader. The authors should simplify the language, break down complex sentences, and improve figure annotations to make the paper more accessible to a broader audience.

We have modified the manuscript to make it easier for the reader. In relation to Figure 4, we have now made it clearer by colour coding the curves and data based on the different structures. We have also added labels to the structures. We have also added the inward-facing narrow data shown in the previous SI Figure 11 into this figure now (i.e. new Figure 4) as requested by the Reviewer.

2）Some abbreviations (e.g., SLiPT, TMD) are used without explanation when first introduced. Ensure all abbreviations are defined at first use.

3）PDB accession codes for cryo-EM structures should be provided.

4）Some figures (e.g., Figure 2) could benefit from higher resolution and clearer labels to highlight key structural features.

These points have been corrected

Reviewer 3:

1) To facilitate the understanding of the data presented in Table 1, it would be good to highlight in the structures on the first column the pairs of amino acids (connected by a dotted line) for which the three distances are listed in this table. Why the periplasmic pair 349-349 (presented in Fig. 2) was not included in the table? Adding the membrane lines to highlight the transmembrane region on the structures (as in Fig. 2) would also increase the clarity of the representation. In addition, the Ca-Ca distances are given with sub-Angstrom resolution, but they could be better rounded without digit after the comma because sub-Angstrom resolution is not accurate or necessary here.

We thank the Reviewer for their suggestion. We have now included the distances for the periplasmic pair L349-L349', rounded Ca-Ca distances, and added lines to highlight the transmembrane region. Due to the relatively small size of the structures, it is practically difficult to highlight each amino-acid pair connected by a dotted line, which would make it unclear to the reader.

2) The authors should comment on why DEER/PELDOR data were only obtained in detergent-solubilized samples and not in nanodiscs. The data in nanodiscs could have added additional information on the conformational ensemble providing a direct

correlation to the cryo-EM structures, especially because the outward facing conformation could only be populated in nanodiscs at pH 7.5 with 2 Fabs.

We thank the Reviewer for their comment. In our Science Advances paper (10.1126/sciadv.abj5363) we also determined the outward-facing conformation without Fabs, and the structure was nearly identical to the Fabs-bound one, suggesting that the Fabs do not drive this conformation. The inward facing structures in nanodisc and DDM are also very similar, suggesting that the lipid environment does not alter its conformation. EPR measurements in nanodiscs although useful are out of the scope of this particular study. Further, we show that SbmA in DDM samples very similar states of all the ones depicted in the cryo-EM structures we present here and describe their unrestricted dynamic motions in DDM solution. Notably, through our reported PELDOR measurements in DDM we demonstrate SbmA's pH dependence (i.e. the aim of these sets of experiments) and the shifts occurring in its conformational equilibrium upon pH changes, which are robust and clear. In support to PELDOR, RT solution CW EPR data also show a rigidification of the spin labelled sites on both membrane sides (i.e. cyto- and peri-plasmic ends) upon significant pH decrease (i.e. from 6 to 4.5).

3) Fig 2: the authors show surface maps for 4 states in b but in c (there is also a typo, they name c as b in the legend) only 3 views are shown. The inward facing narrow conformation from b should also be presented in c.

We have updated the figure.

4) Fig 4: the simulated distance distribution is misplaced below the experimental data. The legend is very long and could be shortened (e.g. part of the description can go to the M&M). The simulation on the inward-facing narrow shown in Fig. S11 should be added for completeness directly in Fig. 4 (as well as the structural model), to highlight the similarity between the distances in different states. It is otherwise difficult to follow the interpretation: 'At pH 4.5 the narrow distance distribution ismost consistent with the inward-facing-narrow state (Supplementary Fig. 11) with some contribution from one or more states with structures closer to the inward-occluded state.'

Thank you, we have now added the inward-facing narrow MMM modelling and cryo-EM structure to Figure 4 and condensed the legend. We have also included additional analysis using DeerLab for our PELDOR data, now presented in Supplementary Figure 11. The conclusions are consistent with our initial DeerAnalysis (and validation) analysis presented in Figure 4.

5) Interpretation of CW data from Fig 4: "The EPR spectral lines 388 showed large differences when recorded at different pH values, indicating that both 389 H127R1 and T294R1 become highly rigid at pH 4.5 compared to pH 8 and 6." Some spectral changes are visible at pH 4.5 (yet the spectrum is not in the rigid limit), but there is

also a “rigid” component already present at pH 8 and 6. The spectra should be compared in the SI (amplitude normalized), to better show the differences. Note: The baseline of the spectrum at pH 4.5 in panel a seems not to be properly corrected and the high field peak results non-symmetric. Another concern is that the authors used MTSL, which can get released from the protein upon disulfide bond breakage. The authors should confirm that the very mobile component seen at pH 6 and 8 (main difference to spectra at pH 4.5) is not arising from partially released label. The conclusive statement: ‘Collectively, the CW-EPR and PELDOR data suggests that acidic pH rigidifies both peri- and cytoplasmic gate regions of SbmA and pushes the SbmA conformational ensemble towards the inward-occluded state.’ is not fully corroborated by the cw data and by the PELDOR data in Fig. 4d.
6) It is not clear why the modulation depth of the DEER traces in Figure 4 (especially in panel f: 20%, 10%, 25% for pH 8,6,4.5) varies among the different pH values. The authors should comment on that. If the mobile component in the cw spectra correlates with the lower modulation, it is tempting to suggest that the mobile component is due to label release. Otherwise, another possible explanation should be provided.

(points 5+6 are addressed together)

We thank the Reviewer for their comment and appreciate their point that the change in mobility we observed might be attributed to the release of the MTSL. Although this is an extreme case scenario to occur, we recognise this may be a possibility and we have thus now retested this to ensure that these changes are not due to partial release of MTSL and this phenomenon is excluded from happening. First, we have undergone through a rigorous protein sample preparation and multi-step quality assessment here for all protein samples we presented. All samples have been subjected to Size Exclusion Chromatography (SEC) following spin labelling to remove all the MTSL excess, and also immediately after samples have been tested by RT CW EPR spectroscopy and were flash frozen for DEER/PELDOR measurements. If there even was a “partial” removal of MTSL during these 5-10 min between SEC and CW EPR analysis we would expect this should have happened to a greater and a global extent and not among different conditions for the same protein site. . Nevertheless, to test Reviewer’s point, we performed similar measurements at different pHs on SbmA in presence of the antibiotic bleomycin and present below for the Reviewer’s consideration and assessment. These data are not part of this manuscript, but we are sharing them here to provide additional context.

CW EPR data for SbmA H127R1and T294R1 (+Bleomycin) different pH conditions and respective PELDOR data and analysis

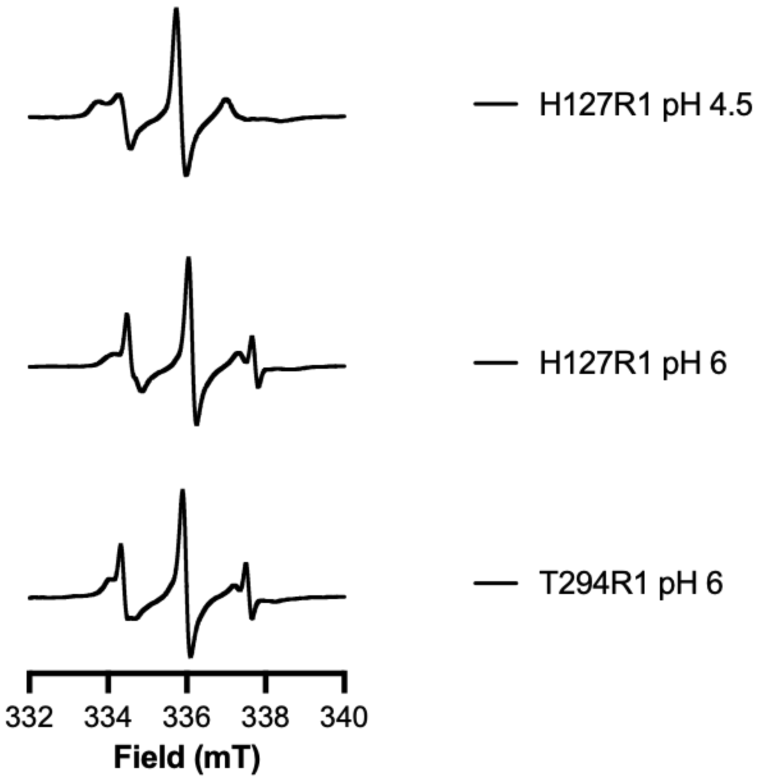

H127R1 pH 4.5 +/- Bleomycin

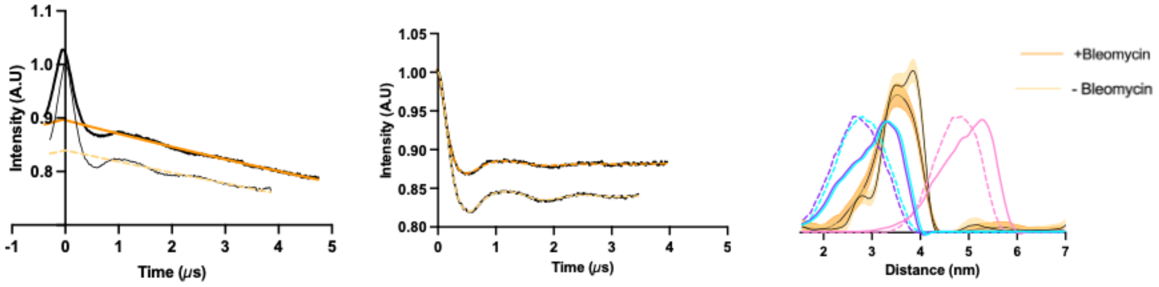

H127R1 pH 6 +/- Bleomycin

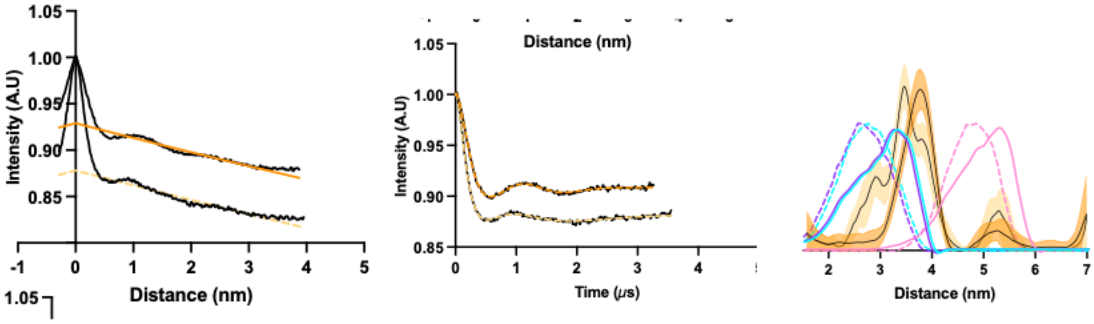

T294R1 pH 6 +/- Bleomycin

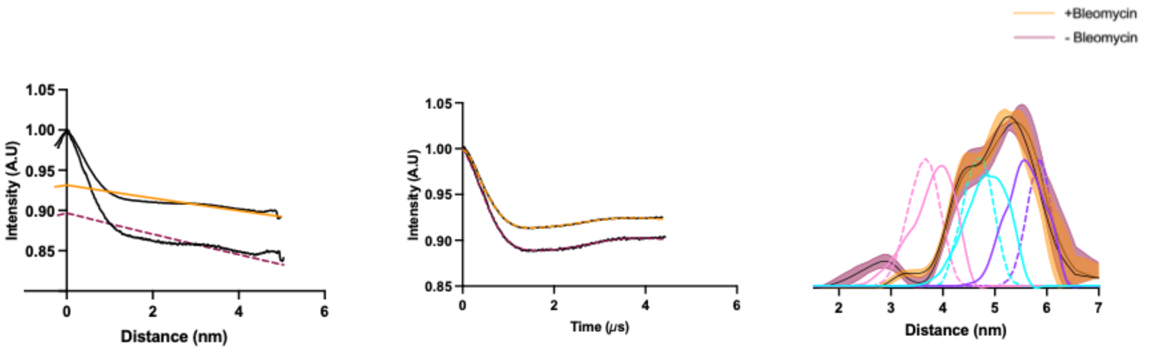

In the presence of Bleomycin the CW EPR spectra are highly similar to the ones we presented for SbmA H127R1 or T294R1 and they originate from the same protein samples (i.e. protein samples were split at equal volumes before the addition of bleomycin)/ Therefore, these particular protein samples must have identical spin labelling efficiency) with the ones presented for the *apo* SbmA state in the original manuscript. However, at several pH values, the modulation depth differs for the +/- bleomycin conditions. This variation in modulation depth does not though follow a specific trend and is consistent with the data the Reviewer refers to in the manuscript. We do not know the specific origin of this particular effect, given that the modulation depth depends on several factors, one of which could be the unexpected “removal” of the highly stable covalent linkage between Cys and MTSSL. If there was a direct link of the modulation depth with the partial removal of MTSSL, we would expect to see a decreasing modulation depth trend with pH, however in our data this is not the case (pH 4.5 > pH 6 < pH 8). The modulation depth is influenced by multiple factors beyond the labelling efficiency, such as the relaxation times of individual spin labelling sites, aggregation or high local concentration, protein complex dissociation, sample homogeneity, the vast majority of which we have assessed through our rigorous biochemical analysis (SEC, SDS Gels et al), implemented immediately prior to PELDOR sample preparation.

We have now updated the CW EPR spectra considering the baseline correction as suggested by the Reviewer in the revised manuscript.

7) One set of DEER data at different pH values is shown for two pairs of labels (127 and 294). However, at least a biological repeat should be shown, to corroborate the changes observed.

We are confident in the high quality of our data and believe that “biological” repeats (although we have implemented several “technical” repeats here) were not necessary in such case. In particular, the DEER data show clear and well-defined dipolar modulations for two independent (and distant) protein sites which has enabled reliable extraction of distance distributions and allowed for direct comparison with *in silico* modelling using newly obtained high resolution SbmA structures in multiple states. The consistency of our data combined with the additional measurements (including technical repeats) on the bleomycin samples,

also provide further support for the reliability. Importantly, (and aligning with the EPR community's recent white paper, Schiemann et al., JACS, 2021) we have included two representative spin labelling sites located on either side of the membrane in order to allow us to draw firm conclusions on the conformational ensemble of SbmA in solution. the dynamics of SbmA. In addition, we recorded and present here PELDOR traces with high SNR, long time windows that yieldreliable distributions (within the expected distances), and with both mutant sites showing strong visual oscillations in their raw time domain spectra.Notably,, all these facts described above are in accordance with (and beyond) the EPR community's recent white paper (Schiemann et al., JACS, 2021)

8) DEER data from Fig. 4: in accordance with the guidelines form the DEER/PELDOR community (Schiemann et al., J. Am. Chem. Soc. 2021, 143, 43, 17875–17890), the authors should show another, user-unbiased, analysis for validation of the distance distributions, e.g. neural network, and present it in SI. In the M&M, the information about the validation of the Tikhonov analysis is also missing (which model for the background, which range of parameters were validated).

We are grateful to the reviewer for their comment. We did indeed used multiple forms of analysis for our data (we corroborated our data processing using CDA and DeerLab, in accordance with Schiemann et al., JACS, 2021). We have now added the DeerLab analysis to the revised manuscript and results are included in theSupplementary Figure 11 The DEERLab analysis accounts for the "2+1" contribution and we have also expanded the materials and methods section to include this new method analysis information and references. Importantly, conclusions of the DeerLab analysis are consistent with our previous analysis of the PELDOR data presented in Figure 4.

9) The assignment of the outward open conformation based on the PELDOR data (Fig. 4d) is ambiguous. There might be still some residual 2+1 signals at the end of the PELDOR traces that could interfere with the determination of the long-distance peaks. The V(t) traces of Fig. 4 clearly show an increase in intensity towards the end, which is then fitted in the F(t) analysis. Indeed, the authors cut the last 600 ns of the traces because of the 2+1 artefact, but it is possibly not sufficient. The primary DEER data before the 600 ns cut must be shown in the SI. To verify if the oscillations at the end of the trace are real or due to residual 2+1 signal, it is necessary to detect traces with shorter length and compare them with the longer traces (without 600 ns cut) to identify is the 2+1 signal moves. Alternatively, softer pump pulses can be used to verify if the oscillations at the end of the trace persist or the software DeerLab can be used to calculate the 2+1 signal. If the 2+1 signal is not ruled out completely, it is not possible to state that: 'At pH 6, and even more at pH 8, main distance components consistent with the outward open SbmA state are also present, although the PELDOR data suggest that these species may only constitute a minor population within the SbmA ensemble'.

We appreciate the reviewer's insightful comment regarding the DEER data analysis. To address the "2+1" effect contribution (T*eucher, M. & Bordignon, E. J. Magn. Reson.* ***296****, 103–111 (2018)*. we have now performed additional data analysis using DeerLab (as also per comment above), and the results remain highly consistent with those presented in Figure 4. We have now included the associated DeerLab data analysis in the Supplementary Figure 11

A major challenge in measuring long distances is the limitation imposed by relaxation times and the experimental setup. While using soft pump pulse could significantly reduce the "2+1" contribution, this approach would come at the expense of modulation depth, which would also be reduced and in turn, this will impact the SNR. An alternative solution is to leverage the capabilities of the Arbitrary Waveform Generator (AWG), now a standard feature in newer Bruker EPR spectrometers, to generate shaped pulses with well-defined excitation bandwidth and minimal side lobes. This approach could help optimize the balance between reducing unwanted contributions and maintaining sufficient modulation depth.

REVIEWER COMMENTS

Reviewer #1

It has taken us a while to digest and evaluate the latest comments from Reviewer #1, about which we have strong concerns. We believe that the request for constant pH simulations is only going to validate our existing experimental data and these are not essential in our opinion and out of the scope of this study. The simulations in our manuscript are part of a bigger picture that emerges from multiple cryo-EM structures, EPR analysis, bioinformatics work and previous mutagenesis. Based on the results from these diverse techniques, which strongly suggest a central role for the glutamate ladder in the mechanism of transport, we designed a focussed set of MD simulations, which explores the role of protonation of each glutamate residue in the ladder. These simulations are well-converged and are supportive of the extensive set of experimental data. In multidisciplinary work the strengths of different methods are combined synergistically, leading to insight that no individual technique is able to obtain, given the current state of the art. We are providing a point-by-point reply to the reviewer below. Performing the additional work will only provide us with limited new insights – if any- and the length they will take will be over >6 months.

To put it in more context why we think this is the case, please see below:

Our well-converged MD simulations in this manuscript are designed to explore individually the role of protonation of each residue of the glutamate ladder. For this, we added protons individually to every glutamate and investigated if their protonation would stabilize the outward-open state or if it would induce a conformational change to the inward-facing state. Fixed protonation state simulations are more efficient in answering this question because they provide a constant driving force. In contrast, the protonation-dynamic allowing constant pH simulations (CpHMD) requires a convergence of the protonation/deprotonation dynamics. While this method is suitable for fast events such as protonation-deprotonation of individual solvent exposed titratable residues (histidine, glutamate and aspartate), the mechanism of SbmA function is rooted in coupling of protonation of the buried glutamates, which are engaged in an extensive interaction network, to large scale conformational changes. Our experimental data show that the switching between outward-facing and inward-facing SbmA is associated with this protonation - conformation coupling. These conformational changes are in the millisecond time regime and this way outside a time regime reachable by CpHMD. We have already experimentally studied the glutamate residues of the glutamate ladder by mutagenesis and functional assays. Please see (DOI: 10.1126/sciadv.abj5363). These experimental data are fully consistent with the results described in this manuscript and corroborate the insights uncovered by MD simulations.

The authors indicate that the five glutamate residues (E193, E203, E269, E276, E378) selected for protonation were chosen based on "previous structural and functional work." However, they do not explicitly link this selection to their own experimental evidence—for example, which of these glutamate mutations most severely impaired SbmA's transport activity, or how the residues'

positions in the transmembrane domain align with the proposed proton translocation path (defined as the "glutamate ladder" in Supplementary Fig. 10). Additionally, the authors exclude E103 from protonation state testing—even though it exhibits a significant pKa shift due to its location—while claiming it cannot participate in membrane-spanning proton transport; this reasoning is unconvincing, as allosteric effects and dynamic influences cannot be ruled out.

In the manuscript we added ‘loss of any of the conserved glutamates rendered the transporter either inactive or severely impaired of transport activity’.

E103 is not part of the glutamate ladder. It forms a hydrogen-bond with the PG lipid and the significant pKa shift might just represent its local environment within the lipid pocket. We do not anticipate allosterism from this residue.

The authors propose a "logical movement" of protons from E203 to E193 to drive conformational change, yet this protonation sequence is inferred only from residue location, rather than being supported by experimental or computational data. While the authors note that inward-facing protonation simulations were conducted, they provide no details on how these simulations either alter or confirm the proposed protonation sequence. As a result, the core mechanism connecting protonation to conformational change remains speculative.

We would like to disagree with the Reviewer. Our previous mutagenesis work identified both residues as essential for transport activity. E203 is the first glutamate of the glutamate ladder and since loss of transport activity by mutagenesis renders the transporter inactive, thus the proposed model. Logically the proton moves along the glutamate ladder as its disruption cannot be bypassed by recruiting the proton by another glutamate along it.

The authors use the full 1 μs trajectory (per simulation repeat; Supplementary Table 1) for their analysis—including initial trajectories where RMSD values are still rising (Supplementary Fig. 12), a period that indicates non-equilibrium states. They fail to provide quantitative evidence demonstrating that the simulations reached equilibrium, which raises questions about whether early non-equilibrated data skews distance distributions and undermines the validity of their conclusions.

Figure 12 shows the typical and expected behavior for a large protein moving at equilibrum. The initial frames are always closer to the start, and that is what Figure 12 also reflect. In response to this comment we removed 200 ns of the production to

start quantifying averages after the memory of the starting structure is lost and have updated the figure.

The authors justify their rejection of enhanced sampling techniques (e.g., metadynamics, umbrella sampling) by citing "prohibitively large collective variables". But they do not address other alternative advanced methods that could improve conformational sampling.

The fundamental issue is that the response of SbmA is in the micro- to milisecond timescale and motions are complex. It would help a lot if these conformation triggering changes would be known with atomic resolution to specifically design such simulations. The current manuscript helps to gain knowledge in this respect. Nevertheless, to study the entire conformational change of uptake of SbmA is very interesting, but outside the scope of the current manuscript. The challenges are similar of all methods: Timescales for SbmA to respond to the conformational changes induced by protonation state changes are very long. If a method would partially induce changes, these data are expected to suffer from convergence issues. It is well documented that for methods requiring the definition of collective variables (such as weighted ensemble path sampling, essential dynamics, flooding, or metadynamics), and leaving out an essential degree of freedom leads to artefacts and in reality non-existing energy barriers. Given the complexity of the SbmA structure, structural difference and the changes occurring during the transition, it is unlikely that these can all be identified or predicted. The cryo-EM structures already show several different changes, the number of independent degrees of freedom is therefore high, leading to a formidable sampling challenge. Untargeted methods like parallel tempering or replica exchange with solute tempering are limited by the large size of the SbmA system (requiring many replicas), the physical relevance of high temperatures, convergence issues because of many local minima in the free energy landscape and inefficient sampling because of a frustrated not very steep energy surface.

The authors dismiss CpHMD, claiming it suffers from "significant error in correctly predicting pKa values of buried amino acids." This argument is unpersuasive: the buried glutamates in the glutamate ladder (e.g., E193, E203) are not isolated from solvent (as shown in Supplementary Fig. 10), and modern CpHMD protocols— which the authors neither cite nor discuss—have improved accuracy for such residues. While CpHMD has inherent limitations, it remains a key approach for validating pH-dependent protonation dynamics. Their dismissal thus appears to reflect a simple reluctance to invest additional effort.

The recent improvement of constant pH simulations methodology and force field modifications (cite 2 paper by Grupmueller and Gruenhof) represent a key improved

of the methods. The methods paper by the Grubmueller group (Biand the al., J. Chem. Theory Comput. 2025, 21, 1762−1786) also shows for the small water soluble test system Hen Egg White Lysozyme that conformation-protonation coupling can very slow for a small loop. The paper suggests that the protonation fraction follows the conformational dynamics, which is the limiting factor for convergence. The methods papers also acknowledge the current limitation of the method, in the case of SbmA protonation-conformation coupling and as well, at current software implementation this method does not allow for protons to be passed between residues, but are independently recruited. From experimental data is known, that SbmA uses the proton motor force to energize substrate uptake, with a time range in the milisecond time regime. We have experimentally shown that the glutamates play a key role in this coupling. The glutamates in SbmA form an extensive network of glutamate residues (E193, E203, E269, E276, E378) in the glutamate ladder, one from each protomer, some of which are buried, may of the interaction include arginine or lysine residues. Changes in the protonation state and side-chain side-chain interactions are linked to very slowly (microsecond to miliseconds) converging local and global conformational changes, which dictates the stability of hydrogen bond and salt bridges between the glutamates of the glutamate ladder.

SbmA needs to efficiently sequester the bound protein to use it for coupling to transport, which precludes fast exchange as needed for constant pH simulation to equilibrate. While the new constant pH simulations methods are promising development in the field of MD simulations, the complexity of this particular problem is thus prohibitive because of the intrinsic limitations imposed by the function of SbmA.

Reviewer #3:
- Fig. 4 d is still missing the inward-facing narrow simulation or there is some problem with color rendering which made cyan invisible since the inward-facing wide is plotted blue instead of violet.

The inward facing narrow and inward facing wide simulations yield identical distributions, therefore it is difficult to visualise and plot in a clearer way. We have made the inward facing narrow blue line dotted so that you may be able to discriminate the purple wide state underneath. We agree, but we cannot think of a better or different way to visualise this and we are open to any suggestions.

- The authors still say in the text “The CW EPR spectral lines 388 showed large differences when recorded at different pH values, indicating that both 389 H127R1 and T294R1 become highly rigid at pH 4.5 compared to pH 8 and 6. “ However, the immobile component is already present at pH 8 and 6, ‘highly rigid’ is an overstatement, because the spectra are not yet in the rigid limit for EPR (rotational motion slower than 30 ns). The sentence can be reformulated addressing the fact that the rotational motion at the chosen sites contains multiple components at all pH values, and that the lowest pH correlates with lower dynamics. This is a more general description of the spectra, which avoid overinterpretation of the CW data.

We thank the Reviewer. We now change the text to:

At all pH conditions, the CW EPR spectra reveal multiple motional components, suggesting varying degrees of spin label mobility. Notably, the spin labels at both H127R1 and T294R1 sites experience reduced rotational mobility at pH 4.5 compared to pH 6 and 8. Collectively, the CW-EPR and PELDOR data suggest that acidic pH conditions decrease the overall dynamics of both the periplasmic and cytoplasmic gate regions of SbmA, shifting the conformational ensemble toward the inward-occluded state.

- Concerning label release, the authors explained in detail the steps taken to ensure that the sample contains the least amount of free label, and indeed the area under the very mobile component is very small, therefore it will not largely influence the observed modulation depth in the DEER traces.

We thank the Reviewer for acknowledging this

- Methods: EPR: the authors described EDFS measurements, which were never shown.

We have removed reference to the EDFS from the methods:

For the Reviewer's reference, please see the EDFS below.

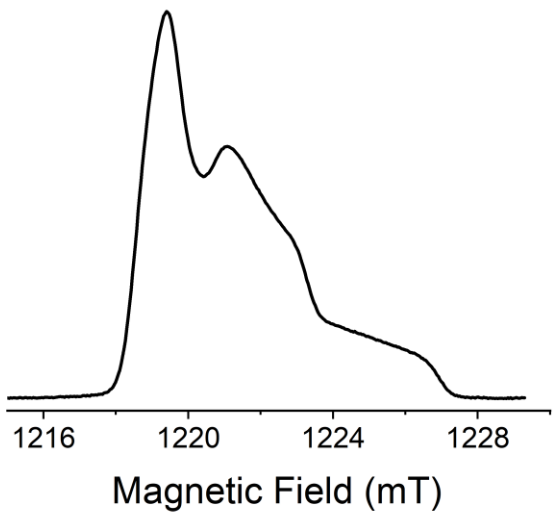

EPR Modeling: it is important to say which MMM version (and Matlab) was used since the same libraries might give slightly different outputs in older versions.

The MMM version used was MMM 2021.1, the Matlab version used was Matlab R2023a.

- Few typos to be corrected: CW: CW EPR, space missing, kHz not KHz.

Thank you, these have been rectified in the manuscript.

Reviewer #1 (Remarks to the Author):

Thank you to the authors for their efforts in revising the manuscript. I fully recognize and appreciate that this work integrates insights from multiple techniques, and I find the overall study valuable. However, I remain unsatisfied with the quality of the MD simulations—particularly the authors' claim that these simulations are "well-converged." For example, the low-populated peaks in the Y368-Y368' distance distribution are derived entirely from one of the six simulation repeats. I cannot accept this as evidence to support the assertion of well-converged simulations, and I do not believe the revision adequately addressed this core concern. I acknowledge that MD simulations are not the central contribution of this project, and thus urge the authors to avoid overstating their conclusions based on MD.

Response: We apologize for using the phrasing "well-converged" in the rebuttal letter. We agree that "well-converged" was an overstatement, as each single trajectory does not cover the full extended of phase space, as indicted by the more or less overlapping distributions of the six independent simulations, while the sum of the six trajectory provides a better description. We emphasize that we had not used the wording "well-converged" in the manuscript text, and now have modified the results and discussion section of the manuscript further to avoid any impression of overinterpretation of the results from the MD simulations.

As suggested, we also moved the MD simulation part to the SI, and now only briefly mention the key MD results in the main text as linked to the experimental structural information.

Regarding CpHMD, I understand that conducting such additional work would be resource-intensive, and I am not insisting on its implementation. Nevertheless, I recommend that the authors incorporate discussions on the potential application of more advanced computational methods to address similar research questions, along with relevant citations. This addition would enhance the manuscript's utility for readers interested in the broader methodological context.

Response: We added a discussion on the estimation of residue protonation, advanced methods (CpHMD) and their limitations to the Supplementary Information Figure 13.
